# Nitrogen-containing andrographolide derivatives with multidrug resistance reversal effects in cancer cells†

**DOI:** 10.1039/d3md00711a

**Published:** 2024-02-26

**Authors:** Joana R. L. Ribeiro, Nikoletta Szemerédi, Bruno M. F. Gonçalves, Gabriella Spengler, Carlos A. M. Afonso, Maria-José U. Ferreira

**Affiliations:** a Research Institute for Medicines (iMed.ULisboa), Faculty of Pharmacy, Universidade de Lisboa Av. Prof. Gama Pinto 1649-003 Lisbon Portugal mjuferreira@ff.ulisboa.pt; b Department of Medical Microbiology, Albert Szent-Györgyi Health Center, Albert Szent-Györgyi Medical School, University of Szeged Semmelweis utca 6 H-6725 Szeged Hungary

## Abstract

Multidrug resistance (MDR) remains a challenging issue in cancer treatment. Aiming at finding anticancer agents to overcome MDR, the triacetyl derivative (2) of the labdane diterpenoid lactone andrographolide (1) underwent the Michael-type addition reaction followed by elimination, yielding twenty-three new derivatives, bearing nitrogen-containing substituents (3–25). Their structures were assigned, mainly, by 1D and 2D NMR experiments. The MDR reversal potential of compounds 1–25 was assessed, by functional and chemosensitivity assays, using resistant human *ABCB1*-gene transfected L5178Y mouse lymphoma cells as a model. Several derivatives exhibited remarkable P-glycoprotein (P-gp) inhibitory ability. Compounds 13 and 20, bearing thiosemicarbazide moieties, were the most active exhibiting a strong MDR reversal effect at 2 μM. Some compounds showed selectivity towards the resistant cells, with compound 5 exhibiting a collateral sensitivity effect associated with significant antiproliferative activity (IC_50_ = 5.47 ± 0.22 μM). Moreover, all selected compounds displayed synergistic interaction with doxorubicin, with compound 3 being the most active. In the ATPase assay, selected compounds exhibited characteristics of P-gp inhibitors.

## Abstract 2

A set of twenty-three new andrographolide derivatives, bearing a nitrogen-containing moiety, is reported. Several derivatives were found to be promising leads for reversing cancer multidrug resistance.

## Graphical abstract

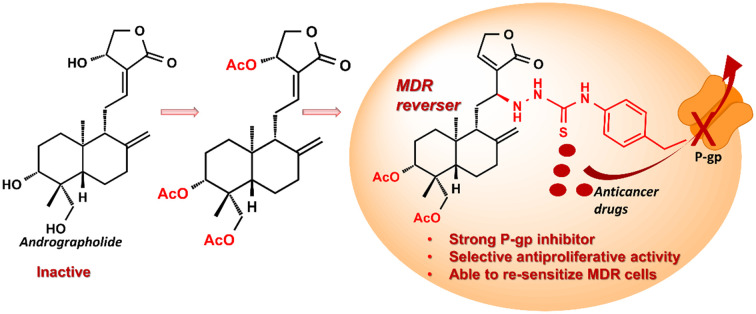

## 1. Introduction

Multidrug resistance (MDR) poses a significant challenge in the realm of chemotherapy, hampering the efficacy of cancer treatment.^1^ This multifactorial phenomenon encompasses various defensive mechanisms such as impaired drug uptake, disrupted DNA damage repair, evasion of drug-induced apoptosis, altered cell cycle checkpoints and arrest, modified drug targets, and enhanced drug efflux mediated by drug transporters. Overexpression of ATP-binding cassette (ABC) transporter proteins, which act as efflux pumps, decreases the intracellular concentration of chemotherapeutic agents, which represents a well-established mechanism contributing to MDR. P-glycoprotein (P-gp/ABCB1), multidrug-resistance associated protein 1 (MRP1), and breast cancer resistance protein (BCRP) are the most relevant drug efflux pumps implicated in multidrug resistance. The inhibition of the efflux activity of these transporters has been considered one of the most important strategies to restore drug sensitivity in MDR cancer cells.^2,3^

The strategy named collateral sensitivity effect (CS) takes advantage of the ability of certain compounds to selectively eliminate MDR cells. MDR cells can be insensitive to many drugs, however, they can display, simultaneously, high sensitivity to other chemotherapeutic agents. Several theories have been proposed to explain this phenomenon, including the potential induction of apoptosis in MDR cells by reactive oxygen species (ROS), expulsion of endogenous survival substrates, disruption of the plasma membrane, and alteration of cellular ATP supply.^4^

Many efforts have been made in the development of multidrug resistance reversers, exploring the two approaches explained above, namely i) the development of drugs with a new mode of action that can inhibit MDR (*e.g.* efflux pumps), and ii) the development of drugs that can re-sensitize MDR tumour cells. However, over the past three decades, the inhibition of P-gp has overwhelmingly dominated research endeavours. While several compounds capable of reversing P-gp-mediated multidrug resistance have been identified, none of them have yet received approval for clinical therapy, primarily due to their high toxicity levels or inadequate *in vivo* efficacy.^5,6^ Consequently, the quest for selective and effective MDR reversal agents that hold clinical utility remains a challenge that requires further investigation.

Natural products, particularly from plants, still represent a prime source of bioactive small molecules as new lead structures for anticancer drug discovery, owing to their ability to modulate a diverse array of molecular mechanisms. In fact, most of the anticancer drugs clinically available are natural products or derivatives of natural products.^7^ Among the myriad natural products, andrographolide (1), a labdane diterpenoid found abundantly in *Andrographis paniculata*, a plant renowned in Southeast Asian traditional herbal medicine, has attracted the attention of researchers due to its broad range of biological activities.^8,9^ It exhibited anticancer properties in various cell lines and *in vivo* tumour models, which have been attributed to diverse mechanisms. Recent investigations have unveiled its potential to reverse drug resistance by acting as a chemosensitizer in multidrug-resistant cancer cell lines through diverse mechanisms. These include the downregulation of the Bcr–Abl oncoprotein in imatinib-resistant chronic myeloid leukemia cells and inhibition of the FLT3 signal pathway in the MV4-11 acute myeloid leukemia cell line, among others.^10–12^

However, the MDR reversing properties of andrographolide are still barely explored and the mechanism of action remains elusive. A study from 2009 caught our attention, as it revealed that andrographolide exerted a dual effect on P-gp ATPase activity: stimulation at lower concentrations (5 μM) and inhibition at higher concentrations (25–100 μM).^13^ In addition, andrographolide was found to be transported by P-gp.^14,15^ Based on these findings, we formulated the hypothesis that this compound could have the ability to modulate the P-gp drug-transport activity in a competitive manner.

Continuing our ongoing efforts to find new MDR-modifying compounds^16–22^ and recognizing that structural modifications of natural scaffolds have proven to be a valuable strategy for enhancing the potency and selectivity of the parent compound, we employed andrographolide as a starting point to synthesize a series of twenty-three new nitrogen-containing analogues. Subsequently, the newly prepared compounds were tested for their antiproliferative properties and P-gp inhibitory potential by the thiazolyl blue tetrazolium bromide (MTT) assay and the rhodamine-123 accumulation assay, respectively. *In vitro* interactions of selected derivatives with the anticancer drug doxorubicin were also studied by a combination assay. To gain insights into their interaction with P-gp, the ATPase assay was also performed to determine whether the compounds are stimulators or inhibitors of P-gp ATPase.

## 2. Results and discussion

### 2.1. Chemistry

**Scheme 1:**
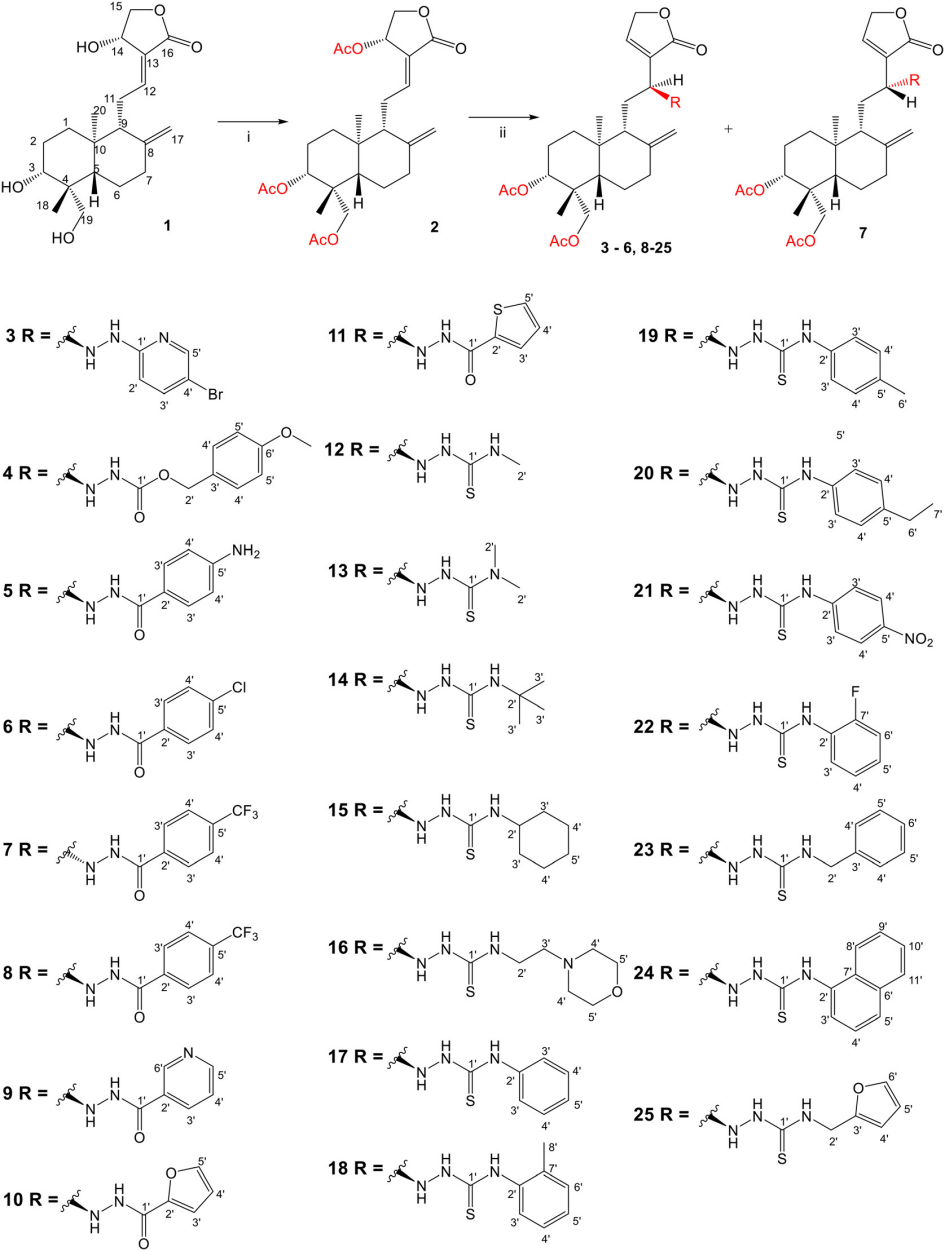
Preparation of triacetyl andrographolide (2) and derivatives 3–25. Reagents and conditions: (i) acetic anhydride (40 equiv.), zinc chloride (0.13 equiv.), room temperature, 2 h; (ii) corresponding hydrazine/hydrazide/thiosemicarbazide (1.2 equiv.) in methanol, room temperature, 18–25 h. The scheme was generated by ChemDraw Professional 15.0. software (PerkinElmer).

Previous studies by our group suggested that the incorporation of nitrogen containing moieties into diterpene structures positively influences the modulation of P-gp efflux function.^23^ In addition, several other studies pointed the presence of nitrogen atoms and aromatic rings as key factors for the MDR reversing activity.^24–26^ Thus, we envisioned that the incorporation of nitrogen and aromatic bearing-features into the andrographolide molecular structure could be an effective strategy for optimizing its MDR reversal properties. As outlined in Scheme 1, our approach started with the protection of the highly reactive hydroxyl groups of andrographolide to prevent the formation of multiple derivatives and as a better leaving group of the C-14 hydroxyl group to facilitate the Michael addition-type reaction, followed by elimination, involving the γ-butyrolactone moiety. This was achieved by reacting andrographolide with acetic anhydride in the presence of ZnCl_2_ to afford the intermediate triacetyl derivative 2. Comparison of the NMR data of compound 2 with those of the starting material revealed the presence of three new carbonyl ester groups (*δ*_C_ 170.9, 170.5, 172.4) as well as three new methyl carbons in the ^13^C NMR spectrum. Also, the ^1^H NMR data showing a paramagnetic shift (Δ*δ*_H_ ≈ + 1.0 ppm) of the geminal proton signals H-19, H-14 and H-3 along with the appearance of three singlets of the methyl protons of the acetyl groups substantiated the compound structure. Afterwards, compound 2 underwent reactions with various aliphatic and aromatic hydrazines, hydrazides, and thiosemicarbazides in methanol, at room temperature, promoting the Michael addition at the C-12 position and the elimination of the C-14 acetyl group to generate a small library of twenty-three new compounds including hydrazine (3), hydrazide (4–11) and thiosemicarbazide (12–25) derivatives (Scheme 1, ii). Most of these reactions predominantly yielded a single epimer at C-12 (12*S*). However, in the case of the reaction involving 4-(trifluoromethyl)-benzhydrazide, both epimers 7 (12*R*) and 8 (12*S*) were generated, yielding 20% and 30%, respectively.

The chemical structures of all these derivatives were assigned based on their NMR data, including comprehensive 2D-NMR experiments such as COSY, NOESY, HMBC and HSQC. When comparing the NMR data with those of the precursor compound 2, besides the additional proton and carbon signals corresponding to the new substituent, the main differences were the absence of the signals corresponding to the vinylic proton H-12 as well as the corresponding carbon signal, which were replaced by new signals at *δ*_H_ ≈ 4.0 and *δ*_C_ ≈ 56.0. Furthermore, the signal corresponding to the methyl protons of the C-14 acetyl group was absent, while a proton signal at *δ*_H_ ≈ 4.80 (H-15), along with the carbon resonances at *δ*_C_ ≈ 132.0 (C-13) and *δ*_C_ ≈ 150.0 (C-14), provided evidence of the new double bond at C-13.

In the case of the hydrazide derivatives 4–11, the ^13^C NMR spectra exhibited a downfield resonance at *δ*_C_ 157.2–167.3 assigned to the carbonyl carbon (–HN–NH–C̲O–R) of the hydrazide moiety. Moreover, the presence of the thiosemicarbazide function in compounds 12–25 was confirmed by a characteristic signal displayed at *δ*_C_ 179.9–185.0, which was assigned to the thiocarbonyl carbon C-1′ (–HN–NH–C̲=S–NH–R). The configuration at C-12 was deduced by NOE correlations observed in the NOESY spectrum (see the ESI†), taking into account *J*_12–11_ coupling values. Thus, in epimer 7, the stereochemistry of this tetrahedral stereocenter was assigned as *R* based on the nuclear Overhauser correlation of H-12 with the β proton at C-9. Moreover, the stereochemistry at C-12 was substantiated by the different *J*_12–11_ coupling values of both epimers 7 (12*R*) (br d, *J* = 8.6 Hz) and 8 (12*S*) (dd, *J* = 9.9, 4.1 Hz), with *J* values found for compound 8 similar to those described in the literature for compounds with *S* configuration at C-12 (dd, *J*_12–11_ = 9.5, 5.5).^27^ A relevant difference was also observed in the H-9 resonance in the ^1^H NMR spectrum. While compound 7 presented a H-9 signal at *δ*_H_ 2.22 ppm, in all other derivatives this signal was observed upfield between *δ*_H_ 1.33 and 1.56 ppm.

### 2.2. Biological activity

#### 2.2.1. Antiproliferative activity

The antiproliferative activity of andrographolide (1) and newly prepared derivatives (2–25) was evaluated using the thiazolyl blue tetrazolium bromide assay. The half-maximal inhibitory concentration (IC_50_) values were determined on sensitive L5178Y mouse T-lymphoma cells (PAR) and the corresponding resistant human *ABCB1*-gene transfected L5178Y subline (MDR) and are summarized in Table 1.

**Table 1:** Antiproliferative effect of compounds 1–25 on sensitive (PAR) and P-gp overexpressing-resistant (MDR) mouse T-lymphoma cells

Compound	IC_50_ (μM) + SD		RR
	PAR	MDR	
1	14.52 ± 1.50	14.95 ± 1.50	1.03
2	13.27 ± 0.73	19.20 ± 1.16	1.45
3	20.61 ± 1.22	12.02 ± 0.71	0.58
4	17.69 ± 1.74	23.42 ± 2.00	1.32
5	12.31 ± 0.89	5.47 ± 0.22	**0.44**
6	14.23 ± 0.22	9.04 ± 0.82	0.64
7	14.23 ± 0.24	15.31 ± 1.58	1.08
8	6.74 ± 0.51	11.77 ± 0.55	1,75
9	12.51 ± 1.26	11.78 ± 0.23	0.94
10	11.05 ± 0.76	15.28 ± 0.40	1.38
11	15.51 ± 1.26	14.95 ± 0.66	0.96
12	44.35 ± 1.19	51.29 ± 3.07	1.16
13	29.14 ± 1.68	40.89 ± 3.35	1.40
14	60.05 ± 2.49	54.18 ± 1.68	0.90
15	75.71 ± 2.65	67.39 ± 2.51	0.89
16	61.48 ± 1.71	58.72 ± 3.65	0.96
17	51.74 ± 0.55	>100	ND
18	56.86 ± 2.18	80.99 ± 0.45	1.42
19	36.07 ± 1.49	47.36 ± 2.05	1.31
20	38.91 ± 0.40	>100	ND
21	16.13 ± 0.70	54.83 ± 3.47	3.40
22	52.61 ± 1.49	70.03 ± 1.55	1.33
23	66.91 ± 1.05	67.60 ± 2.00	1.01
24	52.86 ± 1.53	51.31 ± 2.31	0.97
25	50.14 ± 1.69	71.51 ± 1.87	1.43
DOXO	0.07 ± 0.02	6.82 ± 1.08	95.43

Doxorubicin was employed as a positive control. In general, it could be observed that compounds bearing hydrazine (3), or hydrazide (4–11) moieties displayed higher antiproliferative activities against both sensitive (IC_50_ ranging from 6.74 μM to 20.61 μM) and resistant (IC_50_ ranging from 5.47 μM to 23.42 μM) cell lines than the thiosemicarbazide derivatives, (IC_50_ ranging from 29.14 μM. to 75.71 μM and from 40.89 μM to >100 μM in sensitive and resistant cells, respectively), with exception of compound 21. Compound 8, bearing an aromatic substituent containing an electron withdrawing group at the *para*-position, displayed the lowest IC_50_ value towards the sensitive cell line with (IC_50_ = 6.74 μM). However, a direct relationship between this structural feature and the cytotoxicity towards sensitive cells could not be established, as exemplified by the results of compounds 3 and 6, for instance.

On the other hand, the best antiproliferative activities towards the MDR cells were displayed by the hydrazide derivatives 5 and 6, with IC_50_ values of 5.47 μM and 9.04 μM, respectively.

Considering that some compounds exhibited selectivity against MDR cells, their potential to display the collateral sensitivity effect was assessed by determining the relative resistance ratio (RR = IC_50(resistant)_/IC_50(parental)_). Collateral sensitivity is observed when RR < 0.5, while compounds with RR < 1 exhibit selectivity against resistant cells.^28^ From the analysis of the results (Table 1), it is evident that only compound 5 exhibited potential as a collateral sensitivity agent, as indicated by its RR of 0.44, which is further associated with a significant antiproliferative activity (IC_50_ ≤ 10 μM). Conversely, compounds 3, 6, 14 and 15 demonstrated varying degrees of selectivity towards MDR cells, with RR values ranging from 0.59 to 0.90.

#### 2.2.2. Inhibition of P-glycoprotein efflux activity

Compounds 1–25 were investigated for their ability as P-gp inhibitors on both sensitive and resistant cells, using the standard rhodamine-123 functional assay, by flow cytometry. Rhodamine-123, a known P-gp substrate, was used to evaluate the intracellular retention of this fluorescent dye, which serves as an indicator of P-gp efflux inhibition. Compounds that possess P-gp inhibitory properties enhance the cellular accumulation of rhodamine-123 in the P-gp expressing cells. Based on the IC_50_ values, compounds 1, 2, 4 and 12 to 25 were tested at 2 and 20 μM, while compounds 3 and 5 to 11 at 0.2 and 2 μM. Verapamil, a standard P-gp inhibitor, was employed as a positive control at 20 μM. The fluorescence activity ratio (FAR) values were determined by measuring the cytoplasmic accumulation of rhodamine-123 in sensitive and resistant cancer cells and are presented in Table 2. Compounds displaying FAR values >1 are considered as P-gp inhibitors, and those with FAR values >10 are considered as strong inhibitors.^17^

**Table 2:** Effect of compounds 1–25 on the P-gp mediated rhodamine-123 efflux, in ABCB1-transfected mouse T-lymphoma cells

Compound	R	Conc. (μM)	FAR	Compound	R	Conc. (μM)	FAR
1	Andrographolide	2	0.96	14	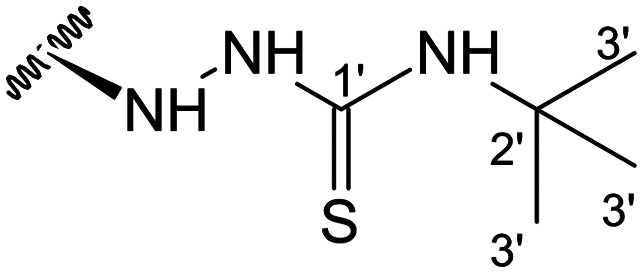	2	1.20
		20	0.82			20	43.52
2	Triacetyl andrographolide	2	1.95	15	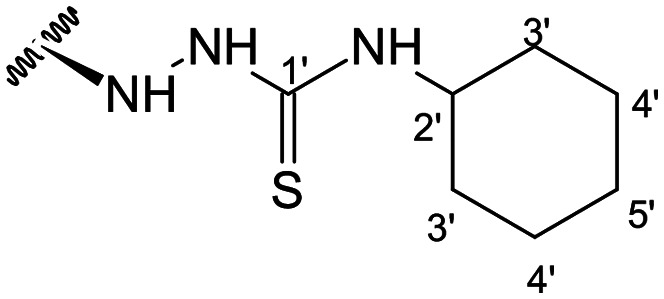	2	18.13
		20	38.65			20	64.94
3	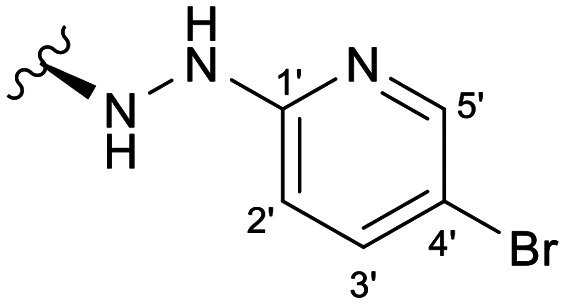	0.2	2.34	16	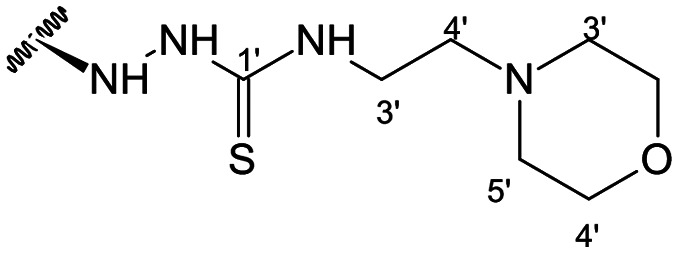	2	1.37
		2	16.12			20	50.23
4	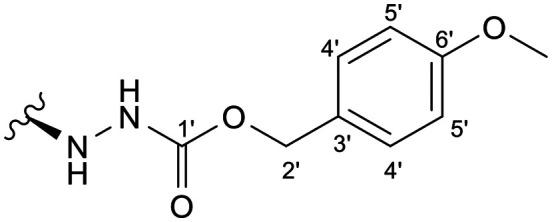	2	1.21	17	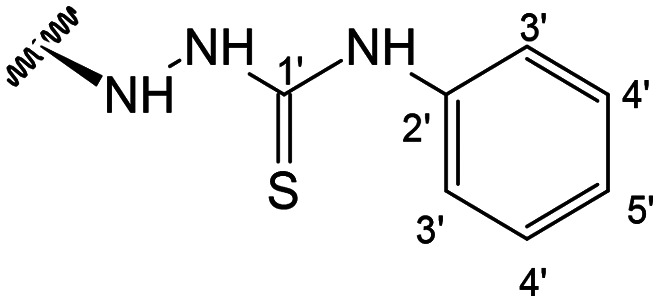	2	7.90
		20	64.99			20	55.07
5	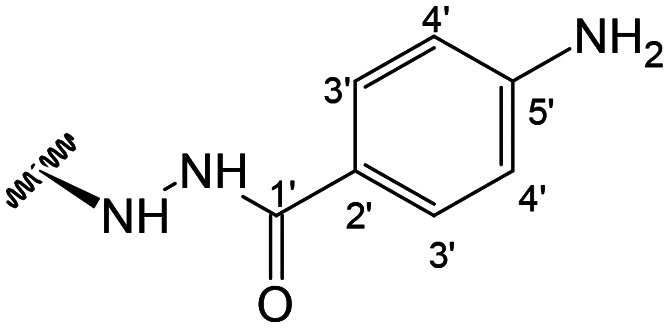	0.2	0.48	18	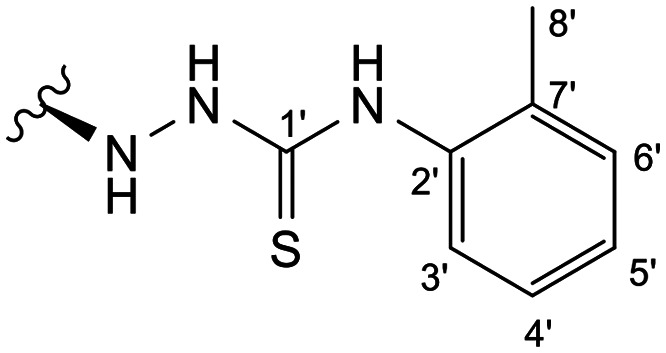	2	1.06
		2	0.48			20	37.12
6	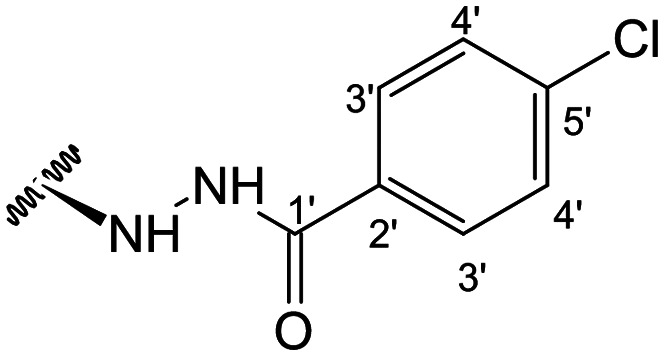	0.2	1.56	19	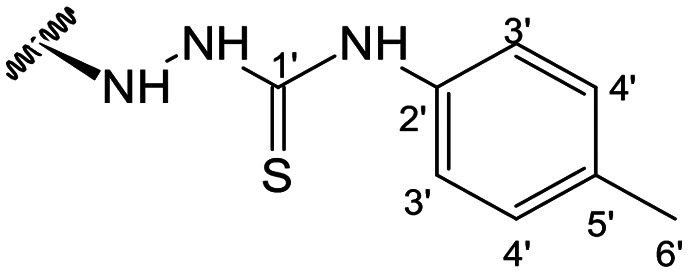	2	18.86
		2	3.36			20	60.26
7	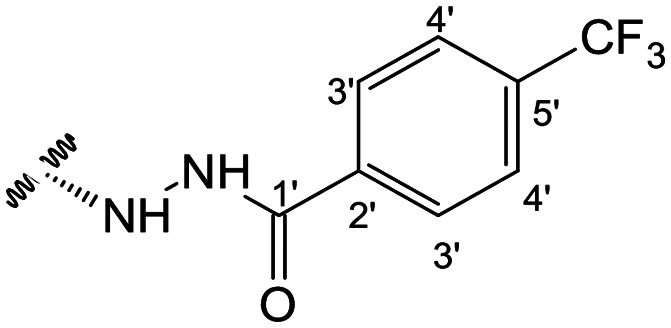	0.2	1.63	20	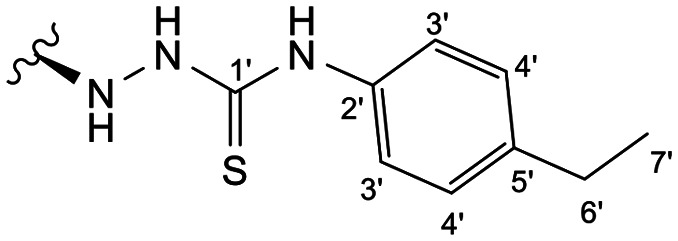	2	61.30
		2	5.19			20	79.48
8	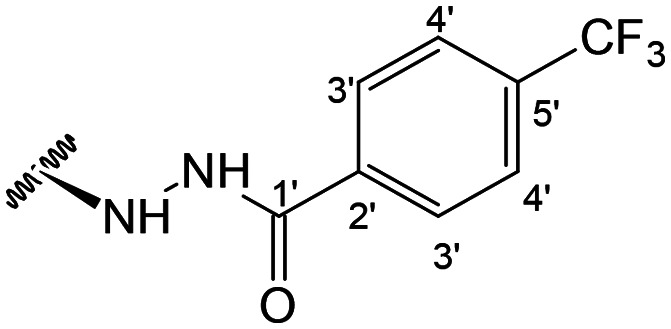	0.2	0.37	21	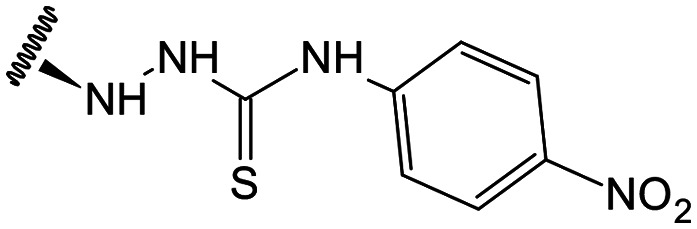	2	27.27
		2	2.27			20	51.95
9	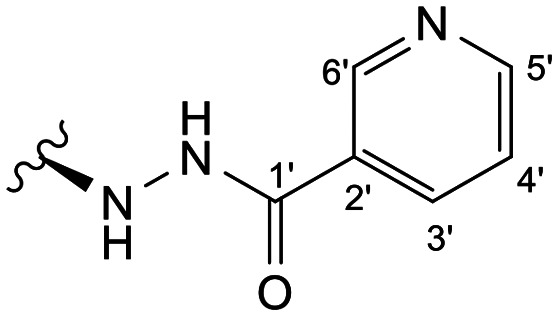	0.2	0.49	22	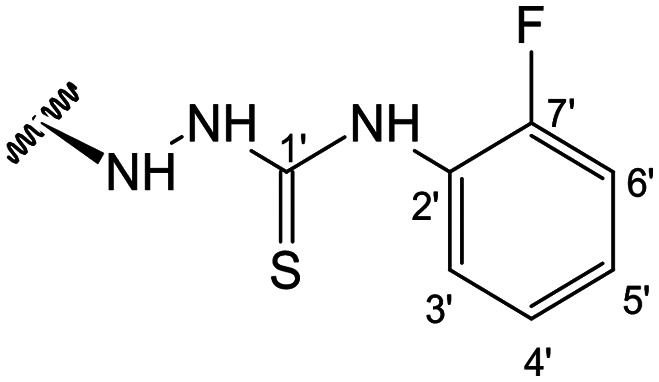	2	1.03
		2	0.32			20	79.33
10	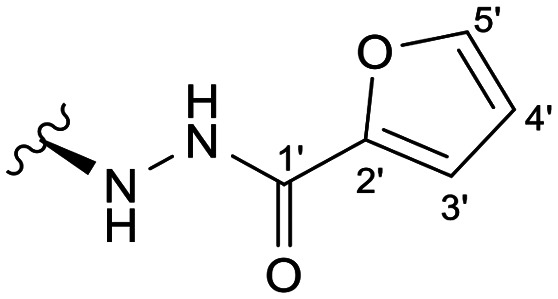	0.2	2.32	23	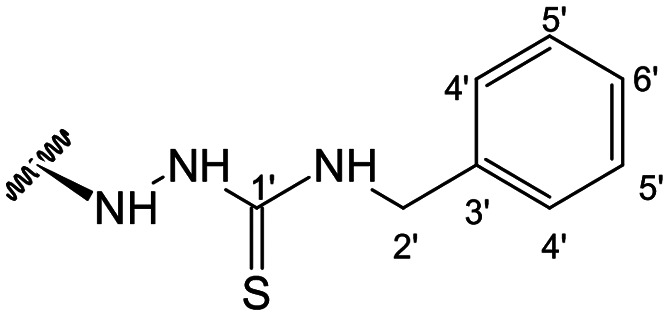	2	1.71
		2	4.28			20	57.14
11	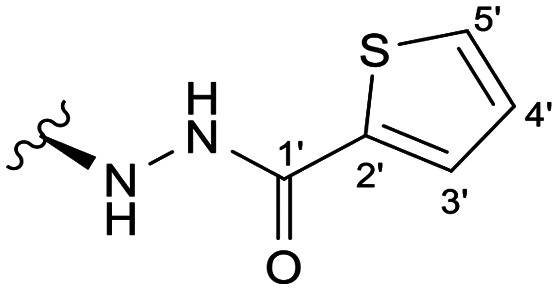	0.2	0.54	24	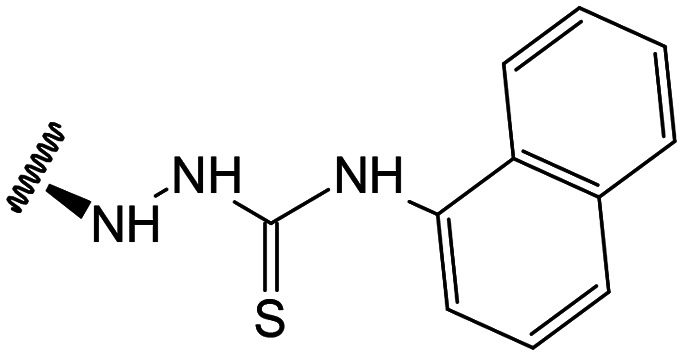	2	14.29
		2	1.89			20	33.46
12	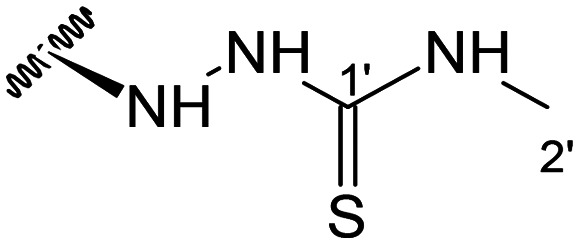	2	0.88	25	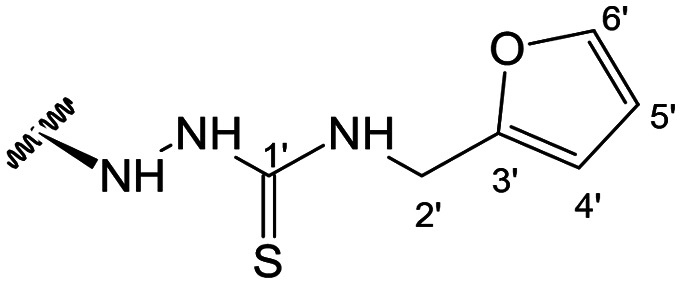	2	1.27
		20	41.85			20	31.12
13	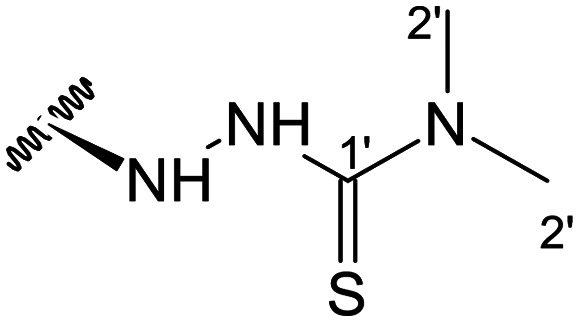	2	58.18	Verapamil		20	5.14
		20	61.30	DMSO		2%	0.96

As demonstrated in Table 2, most of the derivatives showed P-gp modulation properties. The only hydrazine derivative tested, compound 3, proved to be a strong P-gp inhibitor at 2 μM, with a FAR value of 16.12 (3.1-fold greater than that of the positive control verapamil at 10-fold higher concentration).

Among the hydrazide derivatives 4–11, compound 10, bearing a furoic hydrazide moiety, showed a significant FAR value at 0.2 μM, the lowest concentration tested in this set of compounds (FAR = 2.32 at 0.2 μM), whereas compound 4 was a strong P-gp inhibitor at 20 μM (FAR = 64.99; 12.6-fold more active than verapamil).

When comparing FAR values at a concentration of 2 μM, the thiosemicarbazides 13, 15, 19–21 and 24 proved to have strong P-gp inhibitory properties (FAR values ranging between 14.29 and 61.30). At 20 μM all thiosemicarbazides had FAR values significantly higher than verapamil (up to 15.5-fold higher).

This set of derivatives includes compounds with aliphatic, cyclic and aromatic substituents all with P-gp modulatory activities. Thus, these results suggest that the presence of nitrogen bearing moieties (azine (–C–NH–NH–R), azide (–C–NH–NH–CO–R), and thiosemicarbazide (–C–NH–NH–CS–NH–R)) is a critical feature for the activity, as it is a common feature shared by all the compounds exhibiting potential for P-gp inhibition. The absence of activity exhibited by the parental compound (1) (FAR = 0.961 at 2 μM and FAR = 0.815 at 20 μM) aligns well with these findings. It is also noteworthy that among the various motifs attached to the andrographolide scaffold, the introduction of the thiosemicarbazide moiety gave the most significant contribution to the modulatory activity.

#### 2.2.3. Combination assays

**Fig. 1:**
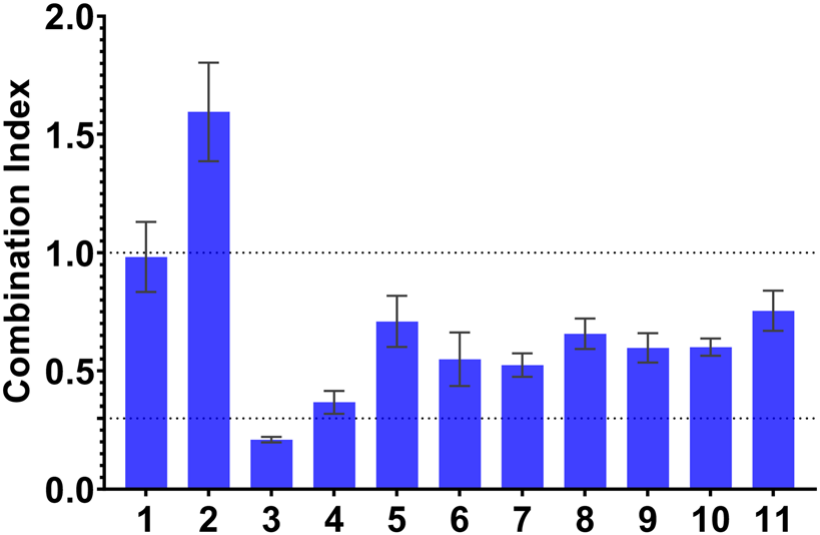
Effect of compounds (1–11) in combination with doxorubicin in L5178Y-MDR cells. Combination index (CI) values are mean ± standard deviation for an inhibitory concentration of 50% (IC_50_). CI < 0.1: very strong synergism; 0.1 < CI < 0.3: strong synergism; 0.3 < CI < 0.7: synergism; 0.7 < CI < 0.9: moderate to slight synergism; 0.9 < CI < 1.1: nearly additive; 1.10 < CI < 1.45: moderate antagonism; 1.45 < CI < 3.30: antagonism.^31,32^

The therapeutic effectiveness of the doxorubicin-based regimens in cancer treatment heavily relies on the expression and function of P-gp, considering doxorubicin's status as a recognized P-gp substrate. Consequently, co-administration of doxorubicin with P-gp efflux modulators could represent a potential strategy to overcome MDR and restore cellular sensitivity to doxorubicin treatment. To investigate the nature of drug interactions between the prepared derivatives and doxorubicin, the checkerboard combination assay was employed. In this assay, L5178Y-MDR mouse lymphoma cells were simultaneously exposed to both selected derivatives (1–11) and the reference drug, in different ratios as described previously.^29,30^ The data obtained were analysed using Calcusyn software to determine the combination index (CI), a concept introduced by Chow and Talalay to quantitively characterize the extent of interaction. A CI value approximately equal to 1 indicates an additive effect, CI < 1 signifies synergism, and CI > 1 refers to antagonism. The obtained CI values are depicted in Fig. 1. As can be observed, all tested compounds, except compound 2, interacted with doxorubicin in a synergistic manner (CI < 1). Compound 3 exhibited the strongest synergistic effect (CI = 0.21).

#### 2.2.4. P-gp ATPase activity

To access the type of interaction between selected compounds (3, 7, 10, 15, 20, 21 and 14) and P-gp, specifically whether they acted as stimulators or inhibitors, a P-gp ATPase activity assay (P-gp Glo™, Promega) was conducted on recombinant human P-gp membranes. This assay enables a direct measurement of effective P-gp-mediated drug transport, by monitoring ATP hydrolysis. The unmetabolized ATP could be quantified using a highly sensitive method based on the firefly luciferin-luciferase assay. Shortly, in the presence of ATP, luciferin is converted into a form that can be catalytically oxidized by luciferase generating oxyluciferin, AMP, and light emission. The intensity of light emitted is directly proportional to the ATP concentration. When P-gp is incubated with ATP, the basal consumption of ATP (basal activity) is reflected by a decrease in the intensity of the signal emitted. Pre-treatment with sodium orthovanadate (vanadate, Na_3_VO_4_), a selective P-gp inhibitor, abolishes P-gp-dependent ATPase consumption. Therefore, the P-gp basal activity (ΔRLU_basal_) is determined by comparing the luminescent signals between Na_3_VO_4_-treated (RLU_Na_3_VO_4__) and untreated (NT) samples (RLU_NT_), while the compound-related P-gp ATPase activity (ΔRLU_TC_) is given by the difference in luminescent signal between Na_3_VO_4_-treated samples (RLU_Na_3_VO_4__) and samples treated (TC) with the selected compounds (RLU_TC_). The activity of compounds can be classified as stimulating, inhibiting or having no effect on P-gp ATPase activity, by comparing with the basal activity (ΔRLU_basal_).

By comparing the compounds' P-gp ATPase activity with the basal activity, the compounds tested can be categorized as inhibitors (ΔRLU_TC_ < ΔRLU_basal_), stimulators/substrates (ΔRLU_TC_ > ΔRLU_basal_), or with no effect on P-gp ATPase activity (ΔRLU_TC_ = ΔRLU_basal_); verapamil (0.5 mM), a known P-gp substrate that stimulates ATPase activity, served as a substrate control (stimulator).^33^

**Fig. 2:**
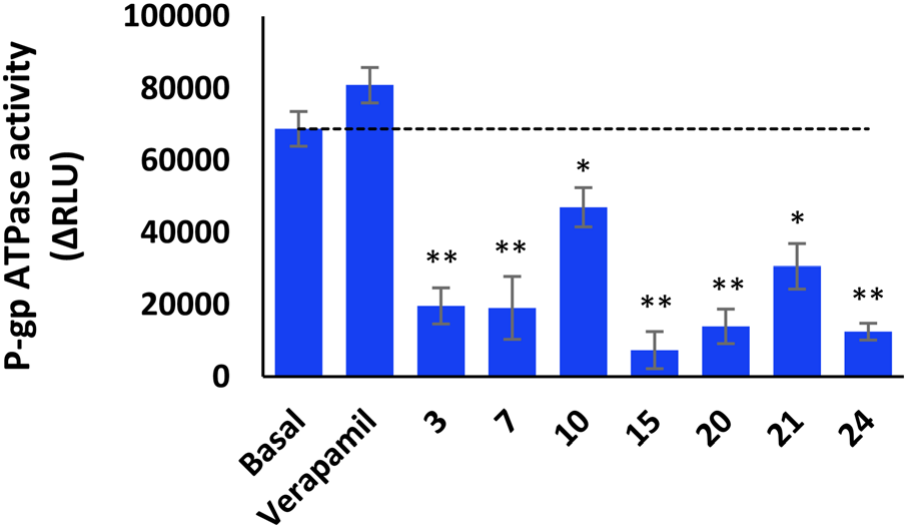
Effect of verapamil and compounds 3, 7, 10, 15, 20, 21, and 24 on P-gp ATPase activity. Verapamil was evaluated at 0.5 mM, and compounds were examined at 25 μM. RLU: relative light unit. ΔRLU_TC_ > ΔRLU_basal_: the compound is a stimulator of P-gp ATPase activity; ΔRLU_TC_ = ΔRLU_basal_: the compound has no effect on P-gp ATPase activity; ΔRLU_TC_ < ΔRLU_basal_: the compound is an inhibitor of P-gp ATPase activity. Results were calculated as mean ± SD of triplicated measurements. Statistical significance was evaluated by unpaired Student's *t*-test (*p < 0.05, **p < 0.01, compared to the basal activity).

As shown in Fig. 2, all the compounds tested (3, 7, 10, 15, 20, 21 and 24) exhibited lower P-gp ATPase activity than the basal activity, indicating that these compounds are P-gp ATPase activity inhibitors.

## 3. Prediction of physicochemical and pharmacokinetic properties of compounds

The SwissADME tool was employed to predict some physicochemical properties of the compounds in order to find correlations with FAR values. The predicted physicochemical properties, which include molecular weight (MW), H-bond acceptors (HBA) and donors (HBD), logarithm of the octanol/water partition coefficient (log *P*) and topological polar surface area (TPSA), are presented in Table S3 (see the ESI†).

It has been found that the appropriate molecular weight range for P-gp inhibitors has been found to vary from 250 to 2000 Da,^34^ an interval in which all derivatives are included. In addition, various classification models for P-gp inhibitors developed over the last decade have identified the compound lipophilicity, expressed as log *P*, as a crucial parameter for distinguishing between P-gp inhibitors and non-inhibitors. Generally, inhibitors have a higher log *P* than the non-inhibitors. It has been suggested that P-gp inhibitors should have a log *P* value of at least 2.92 or higher to facilitate hydrophobic/van der Waals interaction.^35^ All derivatives tested exhibited log *P* values higher than 2.92, with compounds 13, 20, and 21 displaying the best P-gp inhibitory activities at 2 μM, having log *P* values of 3.45, 5,10, and 3.78, respectively. These results support the correlation between lipophilicity and P-gp inhibition. However, when comparing log *P* and FAR values of all the compounds, a poor correlation was found (*r*^2^ value of 0.33; FAR values at 20 μM) (ESI,† Fig. S76). Considering the importance of a favourable pharmacokinetic profile including toxicity (ADMET) in the development of new drugs, the pharmacokinetics of the new derivatives 3–25 were also predicted by means of pKCSM software.^36^ The pharmacokinetic properties of the reference drug verapamil were also determined for comparison purposes and the results are depicted in Table S4 (see the ESI†). As can be observed, compounds 3–25 were found to have poor water solubility (log *S* values ranging from −5.85 to −4.15) similar to that of verapamil (log *S* = −5.23). In addition, these compounds were predicted to have moderate intestinal absorption (64.04–86.66) and reduced ability to cross the blood–brain barrier (BBB), indicating the absence of central nervous system (CNS) side effects.

In terms of their potential toxicity, while concerns regarding the hepatotoxicity may arise for certain compounds, it is important to note that they do not belong to the group of pan-assay interference compounds (PAINS). Furthermore, no Ames toxicity was predicted for these compounds, suggesting a favorable toxicological profile.

## 4. Conclusions

This work was focused on the structural modification of andrographolide (1), as part of ongoing research on the derivatization of natural products as MDR reversers. Thus, by modifying the C-12 position of the andrographolide scaffold, a panel of twenty-three new hydrazine (3), hydrazide (4–11) and thiosemicarbazide (12–25) derivatives was obtained. The antiproliferative activities of all the compounds were evaluated towards both sensitive and P-gp overexpressing-resistant mouse T-lymphoma cells. While the majority of thiosemicarbazides (12–25) revealed reduced cytotoxicity towards both cell lines, some hydrazide derivatives (4–11) exhibited relevant antiproliferative activities. Among these, compound 5, a hydrazide derivative bearing a 4-aminobenzoyl substituent, displayed a collateral sensitivity effect associated with significant antiproliferative activity.

Additionally, several of the twenty-three new compounds demonstrated considerably enhanced P-gp inhibitory efficacy, compared to the parental compound 1. The strongest MDR reversal effect in the transport assay was displayed by compound 20, with a FAR value up to 15 times more effective than the reference inhibitor verapamil. In the drug combination assay, all compounds tested (3–11) showed synergistic interaction with doxorubicin, with compounds 3 and 4 displaying the most pronounced effect.

The ATPase activity assay revealed that the selected compounds (3, 7, 10, 15, 20, 21 and 24) behaved as inhibitors.

Collectively, these findings underscore the value of modifying the andrographolide backbone, particularly through the introduction of thiosemicarbazide moieties, as a promising strategy for identifying new leads in the development of MDR reversal agents. Further investigation into the structure–activity relationships and underlying mechanisms of action is warranted to advance the development of novel MDR reversal agents based on andrographolide derivatives.

## 5. Materials and methods

### 5.1. General experimental procedure

Analytical grade solvents were used. Deuterated chloroform used for NMR analysis was purchased from Merck with a degree of purity higher than 95%. Andrographolide was obtained from a commercial source. Reagent grade chemicals were purchased from Alfa Aesar, Sigma-Aldrich or Merck. Column chromatography was carried out on silica gel (Merck, 230–400 mesh ATM). Thin layer chromatography (TLC) was performed on pre-coated silica gel 60 F_254_ (Merck) and visualized under UV light (*λ* 254 and 366 nm) and/or by exposure to a solution of sulfuric acid–methanol (1 : 1) followed by heating. All the reactions were monitored by TLC. Preparative thin-layer chromatography was performed with silica gel 60 GF_254_ (Merck, 0.040–0.063 mm). NMR spectra (1D and 2D) were recorded on a Bruker Fourier 300 Ultra-Shield using CDCl_3_. Chemical shifts, *δ*, are expressed in ppm (parts per million), and coupling constants, *J*, are expressed in hertz (Hz). Multiplicities are given as: s (singlet), d (duplet), t (triplet), q (quartet), quint (quintuplet) and m (multiplet). Spectra were assigned using appropriate COSY, DEPT, HSQC, and HMBC sequences. The Waters AcquityTM triple quadruple mass spectrometer was used for low-resolution mass spectrometry.

### 5.2. Test compounds

#### 5.2.1. Preparation of 3,14,19-triacetyl andrographolide (2)

To a solution of andrographolide (1, 1.5 g, 4.28 mmol) in acetic anhydride (16.2 mL, 171.2 mmol) was added zinc chloride (76 mg, 0.56 mmol). The mixture was stirred for 2 h at room temperature. After completing the reaction, the excess acetic anhydride was evaporated. The crude reaction was dissolved in dichloromethane : water = 1 : 1. The organic layer was then washed with a saturated solution of sodium bicarbonate, water and brine, dried over anhydrous sodium sulphate, filtered and evaporated. The compound was obtained as a white amorphous powder (quantitative yield). ^1^H-NMR (300 MHz, CDCl_3_) *δ* = 6.99 (1H, td, *J* = 6.8, 1.7 Hz, H-12), 5.91 (1H, dt, *J* = 6.2, 1.7 Hz, H-14), 4.89 (1H, br s, H-17a), 4.60 (1H, dd, *J* = 11.2, 4.3 Hz, H-3), 4.52 (1H, dd, *J* = 11.2, 6.2 Hz, H-15a), 4.51 (1H, br s, H-17b), 4.34 (1H, d, *J* = 11.8 Hz, H-19a), 4.23 (1H, dd, *J* = 11.2, 1.8 Hz, H-15b), 4.11 (1H, d, *J* = 11.8 Hz, H-19b), 2.10 (3H, s, H-14OCOCH_3_), 2.03 (6H, s, H-3OCOCH_3_ and H-19OCOCH_3_), 1.32 (1H, m, H-9), 1.02 (3H, s, H-18), 0.74 (3H, s, H-20) ppm. ^13^C-NMR (75 MHz, CDCl_3_) *δ* = 170.9 (C-19OC̲OCH_3_), 170.5 (C-3OC̲OCH_3_), 170.4 (C-14OC̲OCH_3_), 169.0 (C-16), 150.1 (C-12), 146.5 (C-8), 124.0 (C-13), 108.9 (C-17), 79.6 (C-3), 71.6 (C-15), 67.8 (C-14), 64.7 (C-19), 55.8 (C-5), 55.2 (C-9), 41.3 (C-4), 38.9 (C-10), 37.8 (C-7), 37.0 (C-1), 25.2 (C-11), 24.6 (C-6), 24.2 (C-2), 22.7 (C-18), 21.1 (C-3OCOC̲H_3_ and C-19OCOC̲H_3_), 20.7 (C-14OCOC̲H_3_), 14.5 (C-20) ppm. ESIMS *m*/*z* 477 [M + H]^+^.

#### 5.2.2. Preparation of nitrogen-containing derivatives (3–25)

Compound 2 (0.10–0.27 mmol) was dissolved in methanol (3 mL) and then the appropriate amine derivative (1.2 equiv.) was added. The reaction mixture was stirred for 18–25 h, at room temperature. After evaporating the solvent, the residue was purified by column chromatography (hexane : EtOAc, 10 : 0 to 5 : 5).

##### (12*S*)-5-Bromo-2-hydrazineylpyridine-3,19-diacetoxy-14-deoxy-andrographolide (3)

Obtained from the reaction of compound 2 (100 mg, 0.21 mmol) with 5-bromo-2-hydrazinopyridine (38 mg, 0.25 mmol). The residue was purified by column chromatography to afford compound 3 (90 mg, 71%) as a brown amorphous powder. ^1^H-NMR (300 MHz, CDCl_3_) *δ* = 7.92 (1H, d, *J* = 2.4 Hz, H-5′), 7.52 (1H, dd, *J* = 9.0, 2.4 Hz, H-3′), 7.28 (1H, br s, H-14), 6.88 (1H, d, *J* = 9.0 Hz, H-2′), 4.85 (1H, s, H-17a), 4.81 (2H, br s, H-15), 4.67 (1H, s, H-17b), 4.51 (1H, dd, *J* = 10.6, 5.8 Hz, H-3), 4.30 (1H, d, *J* = 11.8 Hz, H-19a), 4.04 (1H, d, *J* = 11.8 Hz, H-19b), 3.74 (1H, dd, *J* = 10.4, 3.5 Hz, H-12), 2.00 (6H, s, H-3OCOCH_3_ and H-19OCOCH_3_), 1.36 (1H, m, H-9), 0.95 (3H, s, H-18), 0.68 (3H, s, H-20) ppm. ^13^C-NMR (75 MHz, CDCl_3_) *δ* = 173.0 (C-16), 170.9 (C-19OC̲OCH_3_), 170.6 (C-3OC̲OCH_3_), 159.7 (C-1′), 149.9 (C-14), 147.1 (C-5′), 146.7 (C-8), 140.8 (C-3′), 134.0 (C-13), 132.7 (C-4′), 108.8 (C-2′), 107.8 (C-17), 79.7 (C-3), 70.4 (C-15), 64.7 (C-19), 56.3 (C-12), 55.3 (C-5), 52.9 (C-9), 41.3 (C-4), 39.1 (C-10), 38.2 (C-7), 36.7 (C-1), 25.7 (C-11), 24.7 (C-6), 24.2 (C-2), 22.3 (C-18), 21.1 (C-3OCOC̲H_3_), 21.0 (C-19OCOC̲H_3_), 14.6 (C-20) ppm. ESIMS *m*/*z* 604 [M + H]^+^.

##### (12*S*)-4-Methoxybenzyl hydrazinecarboxylate-3,19-diacetoxy-14-deoxy-andrographolide (4)

Obtained from the reaction of compound 2 (100 mg, 0.21 mmol) with 4-methoxybenzyl carbazide (49 mg, 0.25 mmol). The residue was purified by column chromatography to afford compound 4 (65 mg, 51%) as a white amorphous powder. ^1^H-NMR (300 MHz, CDCl_3_) *δ* = 7.24 (2H, d, *J* = 8.5 Hz, H-4′), 7.23* (1H, br s, H-14), 6.84 (2H, d, *J* = 8.5 Hz, H-5′), 5.02 (2H, d, *J* = 3.8 Hz, H-2′), 4.88 (1H, s, H-17a), 4.73** (2H, br s, H-15), 4.68 (1H, s, H-17b), 4.31 (1H, d, *J* = 11.8 Hz, H-19a), 4.26 (1H, dd, *J* = 11.3, 5.1 Hz, H-3), 4.05 (1H, d, *J* = 11.8 Hz, H-19b), 3.88–3.80 (1H, m, H-12), 3.79 (3H, s, H-6′OCH̲_3_), 2.00 (6H, s, H-3OCOCH_3_ and H-19OCOCH_3_), 1.49 (1H, m, H-9), 0.96 (3H, s, H-18), 0.66 (3H, s, H-20) ppm. *Overlapped. **ABX spin system. ^13^C-NMR (75 MHz, CDCl_3_) *δ* = 172.9 (C-16), 170.9 (C-19OC̲OCH_3_), 170.6 (C-3OC̲OCH_3_), 159.7 (C-6′), 157.2 (C-1′), 147.6 (C-14), 147.1 (C-8), 133.9 (C-13), 130.2 (C-4′), 128.1 (C-3′), 113.9 (C-5′), 107.8 (C-17), 79.8 (C-3), 70.2 (C-15), 67.1 (C-2′), 64.7 (C-19), 56.8 (C-12), 55.3 (C-5), 55.2 (C-6′OC̲H_3_) 52.8 (C-9), 41.3 (C-4), 39.2 (C-10), 38.2 (C-7), 36.7 (C-1), 26.1 (C-11), 24.8 (C-6), 24.2 (C-2), 22.6 (C-18), 21.2 (C-3OCOC̲H_3_), 21.1 (C-19OCOC̲H_3_), 14.6 (C-20) ppm. ESIMS *m*/*z* 613 [M + H]^+^.

##### (12*S*)-4-Aminobenzohydrazide-3,19-diacetoxy-14-deoxy-andrographolide (5)

Obtained from the reaction of compound 2 (100 mg, 0.21 mmol) with 4-aminobenzohydrazide (38 mg, 0.25 mmol). The residue was purified by column chromatography to afford compound 5 (77 mg, 65%) as a brown amorphous powder. ^1^H-NMR (300 MHz, CDCl_3_) *δ* = 7.54 (2H, d, *J* = 8.1 Hz, H-3′), 7.29 (1H, br s, H-14), 6.59 (2H, d, *J* = 8.1 Hz, H-4′), 4.87 (1H, s, H-17a), 4.77 (2H, br s, H-15), 4.51 (1H, s, H-17b), 4.50 (1H, dd, *J* = 11.8, 5.4 Hz, H-3), 4.31 (1H, d, *J* = 11.8 Hz, H-19a), 4.03 (1H, d, *J* = 11.8 Hz, H-19b) 3.97 (1H, dd, *J* = 9.9, 4.2 Hz, H-12), 2.00 (6H, s, H-3OCOCH_3_ and H-19OCOCH_3_), 0.94 (3H, s, H-18), 0.65 (3H, s, H-20) ppm. *ABX spin system. ^13^C-NMR (75 MHz, CDCl_3_) *δ* = 173.4 (C-16), 170.9 (C-19OC̲OCH_3_), 170.7 (C-3OC̲OCH_3_), 150.3 (C-5′), 167.3 (C-1′), 148.5 (C-14), 146.9 (C-8), 133.7 (C-13), 128.8 (C-3′), 121.6 (C-2′), 114.1 (C-4′), 108.0 (C-17), 79.9 (C-3), 70.4 (C-15), 64.7 (C-19), 56.8 (C-12), 55.2 (C-5), 52.8 (C-9), 41.2 (C-4), 39.2 (C-10), 38.2 (C-7), 36.6 (C-1), 26.0 (C-11), 24.8 (C-6), 24.2 (C-2), 22.5 (C-18), 21.2 (C-3OCOC̲H_3_), 21.1 (C-19OCOC̲H_3_), 14.6 (C-20) ppm. ESIMS *m*/*z* 568 [M + H]^+^.

##### (12*S*)-4-Chlorobenzohydrazide-3,19-diacetoxy-14-deoxy-andrographolide (6)

Obtained from the reaction of compound 2 (100 mg, 0.21 mmol) with 4-chlorobenzohydrazide (43 mg, 0.25 mmol). The residue was purified by column chromatography to afford compound 6 (35 mg, 28%) as a yellow amorphous powder. ^1^H-NMR (300 MHz, CDCl_3_) *δ* = 7.70 (2H, d, *J* = 8.5 Hz, H-3′), 7.34 (2H, d, *J* = 8.5 Hz, H-4′), 7.32 (1H, br s, H-14), 4.86 (1H, s, H-17a), 4.81* (1H, br s, H-15a), 4.80* (1H, br s, H-15b), 4.71 (1H, s, H-17b), 4.50 (1H, dd, *J* = 10.4, 5.9 Hz, H-3), 4.29 (1H, d, *J* = 11.8 Hz, H-19ª), 4.04 (1H, d, *J* = 11.8 Hz, H-19b), 3.92 (1H, dd, *J* = 9.9, 4.1 Hz, H-12), 2.00 (6H, s, H-3OCOCH_3_ and H-19OCOCH_3_), 1.41 (1H, m, H-9), 0.94 (3H, s, H-18), 0.65 (3H, s, H-20) ppm. *Two overlapped singlets of an ABX spin system. ^13^C-NMR (75 MHz, CDCl_3_) *δ* = 174.0 (C-16), 170.9 (C-19OC̲OCH_3_), 170.6 (C-3OC̲OCH_3_), 166.8 (C-1′), 148.8 (C-14), 146.8 (C-8), 138.1 (C-5′), 133.5 (C-13), 130.1 (C-2′), 128.9 (C-4′), 128.5 (C-3′), 107.9 (C-17), 79.8 (C-3), 70.4 (C-15), 64.7 (C-19), 56.7 (C-12), 55.2 (C-5), 52.8 (C-9), 41.2 (C-4), 39.1 (C-10), 38.2 (C-7), 36.7 (C-1), 25.9 (C-11), 24.7 (C-6), 24.2 (C-2), 22.6 (C-18), 21.2 (C-3OCOC̲H_3_), 21.1 (C-19OCOC̲H_3_), 14.5 (C-20) ppm. ESIMS *m*/*z* 587 [M + H]^+^.

##### (12*R*)-4-(Trifluoromethyl)benzohydrazide-3,19-diacetoxy-14-deoxy-andrographolide (7) and (12*S*)-4-(trifluoromethyl)benzohydrazide-3,19-diacetoxy-14-deoxy-andrographolide (8)

Obtained from the reaction of compound 2 (100 mg, 0.21 mmol) with 4-(trifluoromethyl)-benzhydrazide (51 mg, 0.25 mmol). The residue was purified by column chromatography to afford compound 7 (26 mg, 20%) as a white amorphous powder and compound 8 (39 mg, 30%) as a yellow amorphous powder. Compound 7: ^1^H-NMR (300 MHz, CDCl_3_) *δ* = 7.85 (2H, d, *J* = 8.1 Hz, H-3′), 7.68 (2H, d, *J* = 8.1 Hz, H-4′), 7.37 (1H, br s, H-14), 4.91 (1H, s, H-17a), 4.80* (2H, br s, H-15), 4.64 (1H, s, H-17b), 4.59 (1H, dd, *J* = 10.6, 5.8 Hz, H-3), 4.35 (1H, d, *J* = 11.8 Hz, H-19a), 4.09 (1H, d, *J* = 11.8 Hz, H-19b), 3.85 (1H, br d, *J* = 8.6 Hz, H-12), 2.03 (6H, s, H-3OCOCH_3_ and H-19OCOCH_3_), 2.23 (1H, m, H-9), 1.01 (3H, s, H-18), 0.71 (3H, s, H-20) ppm. *ABX spin system. ^13^C-NMR (75 MHz, CDCl_3_) *δ* = 173.8 (C-16), 170.9 (C-19OC̲OCH_3_), 170.5 (C-3OC̲OCH_3_), 165.3 (C-1′), 147.2 (C-14), 145.9 (C-8), 135.8 (C-2′), 135.7 (C-5′), 133.8 (C-13), 127.4 (C-3′), 125.8 (C-4′), 121.7 (C-6′), 107.7 (C-17), 79.8 (C-3), 70.5 (C-15), 64.8 (C-19), 56.2 (C-12), 55.2 (C-5), 52.4 (C-9), 41.3 (C-4), 39.0 (C-10), 38.2 (C-7), 36.8 (C-1), 26.8 (C-11), 24.8 (C-6), 24.2 (C-2), 22.3 (C-18), 21.2 (C-3OCOC̲H_3_), 21.1 (C-19OCOC̲H_3_), 14.6 (C-20) ppm. ESIMS *m*/*z* 621 (M + H)^+^.

Compound 8: ^1^H-NMR (300 MHz, CDCl_3_) *δ* = 7.89 (2H, d, *J* = 8.1 Hz, H-3′), 7.65 (2H, d, *J* = 8.1 Hz, H-4′), 7.33 (1H, br s, H-14), 4.89 (1H, s, H-17a), 4.88* (1H, dd, *J* = 18.4, 1.4 Hz, H-15a), 4.80* (1H, dd, *J* = 18.4, 1.6 Hz, H-15b), 4.73 (1H, s, H-17b), 4.51 (1H, dd, *J* = 10.6, 5.8 Hz, H-3), 4.30 (1H, d, *J* = 11.8 Hz, H-19a), 4.05 (1H, d, *J* = 11.8 Hz, H-19b), 3.96 (1H, dd, *J* = 9.9, 4.1 Hz, H-12), 2.00 (6H, s, H-3OCOCH_3_ and H-19OCOCH_3_), 1.48 (1H, d, *J* = 9.9 Hz, H-9), 0.95 (3H, s, H-18), 0.66 (3H, s, H-20) ppm. *ABX spin system. ^13^C-NMR (75 MHz, CDCl_3_) *δ* = 173.4 (C-16), 170.9 (C-19OC̲OCH_3_), 170.5 (C-3OC̲OCH_3_), 165.8 (C-1′), 148.9 (C-14), 146.8 (C-8), 135.8 (C-2′), 135.8 (C-5′), 133.7 (C-13), 127.6 (C-3′), 125.7 (C-4′), 121.7 (C-6′), 107.9 (C-17), 79.7 (C-3), 70.5 (C-15), 64.7 (C-19), 56.8 (C-12), 55.3 (C-5), 52.9 (C-9), 41.2 (C-4), 39.1 (C-10), 38.2 (C-7), 36.8 (C-1), 25.8 (C-11), 24.8 (C-6), 24.1 (C-2), 22.3 (C-18), 21.1 (C-3OCOC̲H_3_), 21.0 (C-19OCOC̲H_3_), 14.5 (C-20) ppm. ESIMS *m*/*z* 621 [M + H]^+^.

##### (12*S*)-Nicotinohydrazide-3,19-diacetoxy-14-deoxy-andrographolide (9)

Obtained from the reaction of compound 2 (100 mg, 0.21 mmol) with nicotinic hydrazide (35 mg, 0.25 mmol). The residue was purified by column chromatography to afford compound 9 (30 mg, 25%) as a white amorphous powder. ^1^H-NMR (300 MHz, CDCl_3_) *δ* = 8.98 (1H, s, H-6′), 8.71 (1H, d, *J* = 4.7 Hz, H-5′), 8.13 (H, dt, *J* = 8.0, 1.9 Hz, H-3′), 7.40 (H, dd, *J* = 8.0, 4.7 Hz, H-4′), 7.38 (1H, br s, H-14), 4.93 (1H, s, H-17a), 4.86* (2H, br s, H-15), 4.77 (1H, s, H-17b), 4.56 (1H, dd, *J* = 10.9, 5.5 Hz, H-3), 4.34 (1H, d, *J* = 11.8 Hz, H-19a), 4.09 (1H, d, *J* = 11.8 Hz, H-19b), 3.98 (1H, dd, *J* = 9.6, 4.3 Hz, H-12), 2.04 (6H, s, H-3OCOCH_3_ and H-19OCOCH_3_), 1.56 (1H, d, *J* = 9.9 Hz, H-9), 0.99 (3H, s, H-18), 0.72 (3H, s, H-20) ppm. *ABX spin system. ^13^C-NMR (75 MHz, CDCl_3_) *δ* = 173.4 (C-16), 170.9 (C-19OC̲OCH_3_), 170.5 (C-3OC̲OCH_3_), 165.3 (C-1′), 152.4 (C-5′), 148.2 (C-14), 147.9 (C-6′), 147.0 (C-8), 135.3 (C-3′), 134.0 (C-13), 128.6 (C-2′), 123.7 (C-4′), 107.5 (C-17), 79.8 (C-3), 70.5 (C-15), 64.7 (C-19), 56.8 (C-12), 55.3 (C-5), 52.9 (C-9), 41.3 (C-4), 39.2 (C-10), 38.2 (C-7), 36.8 (C-1), 26.1 (C-11), 24.8 (C-6), 24.2 (C-2), 22.5 (C-18), 21.2 (C-3OCOC̲H_3_), 21.1 (C-19OCOC̲H_3_), 14.6 (C-20) ppm. ESIMS *m*/*z* 554 [M + H]^+^.

##### (12*S*)-Furan-2-carbohydrazide-3,19-diacetoxy-14-deoxy-andrographolide (10)

Obtained from the reaction of compound 2 (100 mg, 0.21 mmol) with 2-furoic hydrazide (32 mg, 0.25 mmol). The residue was purified by column chromatography to afford compound 10 (64 mg, 56%) as a yellow amorphous powder. ^1^H-NMR (300 MHz, CDCl_3_) *δ* = 7.40 (1H, d, *J* = 1.8 Hz, H-5′), 7.32 (1H, br s, H-14), 7.08 (1H, d, *J* = 3.5 Hz, H-3′), 6.46 (1H, dd, *J* = 3.5, 1.8 Hz, H-4′), 4.89 (1H, s, H-17a), 4.82* (2H, br s, H-15), 4.73 (1H, s, H-17b), 4.52 (1H, dd, *J* = 11.1, 5.3 Hz, H-3), 4.31 (1H, d, *J* = 11.8 Hz, H-19a), 4.05 (1H, d, *J* = 11.8 Hz, H-19b), 3.94–3.87 (1H, m, H-12), 2.00 (6H, s, H-3OCOCH_3_ and H-19OCOCH_3_), 1.54 (1H, d, *J* = 10.7 Hz, H-9), 0.95 (3H, s, H-18), 0.68 (3H, s, H-20) ppm. *ABX spin system. ^13^C-NMR (75 MHz, CDCl_3_) *δ* = 173.1 (C-16), 170.9 (C-19OC̲OCH_3_), 170.6 (C-3OC̲OCH_3_), 158.2 (C-1′), 147.8 (C-14), 147.1 (C-2′), 146.4 (C-8), 144.4 (C-5′), 133.9 (C-13), 115.0 (C-3′), 112.1 (C-4′), 107.9 (C-17), 79.8 (C-3), 70.3 (C-15), 64.7 (C-19), 57.1 (C-12), 55.2 (C-5), 52.9 (C-9), 41.3 (C-4), 39.2 (C-10), 38.2 (C-7), 36.7 (C-1), 26.2 (C-11), 24.8 (C-6), 24.2 (C-2), 22.3 (C-18), 21.2 (C-3OCOC̲H_3_), 21.1 (C-19OCOC̲H_3_), 14.6 (C-20) ppm. ESIMS *m*/*z* 543 [M + H]^+^.

##### (12*S*)-Thiophene-2-carbohydrazide-3,19-diacetoxy-14-deoxy-andrographolide (11)

Obtained from the reaction of compound 2 (100 mg, 0.21 mmol) with thiophene-2-carbohydrazide (36 mg, 0.25 mmol). The residue was purified by column chromatography to afford compound 11 (58 mg, 50%) as a yellow amorphous powder. ^1^H-NMR (300 MHz, CDCl_3_) *δ* = 7.58 (1H, d, *J* = 3.8 Hz, H-5′), 7.47 (1H, d, *J* = 5.0 Hz, H-3′), 7.34 (1H, br s, H-14), 7.03 (1H, br s, H-4′), 4.89 (1H, s, H-17a), 4.82* (2H, br s, H-15), 4.74 (1H, s, H-17b), 4.52 (1H, dd, *J* = 10.8, 5.5 Hz, H-3), 4.31 (1H, d, *J* = 11.8 Hz, H-19a), 4.05 (1H, d, *J* = 11.8 Hz, H-19b), 4.00–3.93 (1H, m, H-12), 2.00 (6H, s, H-3OCOCH_3_ and H-19OCOCH_3_), 1.47 (1H, m, H-9), 0.96 (3H, s, H-18), 0.67 (3H, s, H-20) ppm. *ABX spin system. ^13^C-NMR (75 MHz, CDCl_3_) *δ* = 173.2 (C-16), 170.9 (C-19OC̲OCH_3_), 170.6 (C-3OC̲OCH_3_), 162.0 (C-1′), 148.9 (C-14), 146.8 (C-8), 136.4 (C-2′), 133.4 (C-13), 130.5 (C-3′), 128.6 (C-5′), 127.8 (C-4′), 108.0 (C-17), 79.8 (C-3), 70.4 (C-15), 64.7 (C-19), 56.9 (C-12), 55.3 (C-5), 52.8 (C-9), 41.2 (C-4), 39.1 (C-10), 38.2 (C-7), 36.7 (C-1), 25.8 (C-11), 24.7 (C-6), 24.2 (C-2), 22.6 (C-18), 21.2 (C-3OCOC̲H_3_), 21.1 (C-19OCOC̲H_3_), 14.6 (C-20) ppm. ESIMS *m*/*z* 559 [M + H]^+^.

##### (12*S*)-*N*-Methylhydrazinecarbothioamide-3,19-diacetoxy-14-deoxy-andrographolide (12)

Obtained from the reaction of compound 2 (50 mg, 0.10 mmol) with 2-methyl-3-thiosemicarbazide (13 mg, 0.13 mmol). The residue was purified by column chromatography to afford compound 12 (22 mg, 40%) as a white amorphous powder. ^1^H-NMR (300 MHz, CDCl_3_) *δ* = 7.36 (1H, t, *J* = 1.7 Hz, H-14), 4.95 (1H, s, H-17a), 4.88* (1H, s, H-15a), 4.87* (1H, s, H-15b), 4.59 (1H, s, H-17b), 4.54 (1H, dd, *J* = 11.1, 5.3 Hz, H-3), 4.32 (1H, d, *J* = 11.8 Hz, H-19a), 4.07 (1H, d, *J* = 11.8 Hz, H-19b), 4.04 (1H, m, H-12), 3.49 (3H, s, H-2′), 2.02 (6H, s, H-3OCOCH_3_ and H-19OCOCH_3_), 1.38 (1H, d, *J* = 11.1 Hz, H-9), 0.98 (3H, s, H-18), 0.71 (3H, s, H-20) ppm. *Two overlapped singlets of an ABX spin system. ^13^C-NMR (75 MHz, CDCl_3_) *δ* = 183.9 (C-1′), 172.9 (C-16), 170.9 (C-19OC̲OCH_3_), 170.6 (C-3OC̲OCH_3_), 150.5 (C-14), 146.9 (C-8), 132.3 (C-13), 107.4 (C-17), 80.2 (C-3), 70.4 (C-15), 64.6 (C-19), 55.3 (C-5), 53.8 (C-12), 52.9 (C-9), 41.2 (C-4), 39.1 (C-10), 38.6 (C-2′), 38.3 (C-7), 36.8 (C-1), 24.9 (C-11), 24.8 (C-6), 24.1 (C-2), 22.3 (C-18), 21.1 (C-3OCOC̲H_3_), 21.0 (C-19OCOC̲H_3_), 14.6 (C-20) ppm. ESIMS *m*/*z* 522 [M + H]^+^.

##### (12*S*)-*N*,*N*-Dimethylhydrazinecarbothioamide-3,19-diacetoxy-14-deoxy-andrographolide (13)

Obtained from the reaction of compound 2 (100 mg, 0.21 mmol) with 4,4-dimethyl-3-thiosemicarbazide (30 mg, 0.25 mmol). The residue was purified by column chromatography to afford compound 13 (72 mg, 64%) as a dark green amorphous powder. ^1^H-NMR (300 MHz, CDCl_3_) *δ* = 7.36 (1H, br s, H-14), 4.90 (1H, s, H-17a), 4.87 (1H, s, H-17b), 4.84* (2H, br s, H-15), 4.53 (1H, dd, *J* = 11.0, 5.6 Hz, H-3), 4.33 (1H, d, *J* = 11.7 Hz, H-19a), 4.06 (1H, d, *J* = 11.7 Hz, H-19b), 3.97 (1H, dd, *J* = 10.0, 4.2 Hz, H-12), 3.19 (6H, s, H-2′), 2.01 (6H, s, H-3OCOCH_3_ and H-19OCOCH_3_), 1.50 (1H, m, H-9), 0.97 (3H, s, H-18), 0.71 (3H, s, H-20) ppm. *ABX spin system. ^13^C-NMR (75 MHz, CDCl_3_) *δ* = 182.9 (C-1′), 173.2 (C-16), 170.9 (C-19OC̲OCH_3_), 170.6 (C-3OC̲OCH_3_), 149.0 (C-14), 146.4 (C-8), 134.0 (C-13), 108.4 (C-17), 79.8 (C-3), 70.2 (C-15), 64.7 (C-19), 56.5 (C-12), 55.3 (C-5), 52.7 (C-9), 41.3 (C-4), 40.5 (C-2′), 39.1 (C-10), 38.3 (C-7), 36.8 (C-1), 25.7 (C-11), 24.8 (C-6), 24.2 (C-2), 22.4 (C-18), 21.2 (C-3OCOC̲H_3_), 21.1 (C-19OCOC̲H_3_), 14.7 (C-20) ppm. ESIMS *m*/*z* 536 [M + H]^+^.

##### (12*S*)-*N*-(*tert*-Butyl)hydrazinecarbothioamide-3,19-diacetoxy-14-deoxy-andrographolide (14)

Obtained from the reaction of compound 2 (112 mg, 0.24 mmol) with 4-*tert*-butyl-3-thiosemicarbazide (42 mg, 0.28 mmol). The residue was purified by column chromatography to afford compound 14 (103 mg, 77%) as a white amorphous powder. ^1^H-NMR (300 MHz, CDCl_3_) *δ* = 7.33 (1H, br s, H-14), 4.87 (1H, s, H-17a), 4.81* (2H, br s, H-15), 4.59 (1H, s, H-17b), 4.49 (1H, m, H-3), 4.27 (1H, d, *J* = 11.7 Hz, H-19a), 4.02 (1H, d, *J* = 11.7 Hz, H-19b), 3.75 (1H, br d, *J* = 9.3 Hz, H-12), 1.98 (6H, s, H-3OCOCH_3_ and H-19OCOCH_3_), 1.44 (9H, s, H-3′), 1.32 (1H, d, *J* = 11.8 Hz, H-9), 0.92 (3H, s, H-18), 0.66 (3H, s, H-20) ppm. *ABX spins system. ^13^C-NMR (75 MHz, CDCl_3_) *δ* = 180.1 (C-1′), 172.6 (C-16), 170.9 (C-19OC̲OCH_3_), 170.6 (C-3OC̲OCH_3_), 150.8 (C-14), 146.6 (C-8), 131.2 (C-13), 107.7 (C-17), 79.6 (C-3), 70.4 (C-15), 64.6 (C-19), 56.6 (C-12), 55.2 (C-5), 53.0 (C-2′), 52.9 (C-9), 41.2 (C-4), 39.1 (C-10), 38.2 (C-7), 36.7 (C-1), 28.9 (C-3′), 25.1 (C-11), 24.7 (C-6), 24.1 (C-2), 22.6 (C-18), 21.1 (C-3OCOC̲H_3_), 21.0 (C-19OCOC̲H_3_), 14.6 (C-20) ppm. ESIMS *m*/*z* 564 [M + H]^+^.

##### (12*S*)-*N*-Cyclohexylhydrazinecarbothioamide-3,19-diacetoxy-14-deoxy-andrographolide (15)

Obtained from the reaction of compound 2 (100 mg, 0.21 mmol) with 4-cyclohexyl-3-thiosemicarbazide (44 mg, 0.25 mmol). The residue was purified by column chromatography to afford compound 15 (96 mg, 77%) as a white amorphous powder. ^1^H-NMR (300 MHz, CDCl_3_) *δ* = 7.35 (1H, br s, H-14), 4.92 (1H, s, H-17a), 4.87* (2H, br s, H-15), 4.62 (1H, s, H-17b), 4.54 (1H, dd, *J* = 11.0, 5.4 Hz, H-3), 4.32 (1H, d, *J* = 11.8 Hz, H-19a), 4.07 (1H, d, *J* = 11.8 Hz, H-19b), 3.77–3.70 (1H, m, H-12), 2.02 (6H, s, H-3OCOCH_3_ and H-19OCOCH_3_), 1.94 (2H, m, H-3′a), 1.72 (2H, m, H-4′a), 1.58 (2H, m, H-5′a), 1.35 (1H, m, H-2′), 1.34 (3H, m, H-4′b and H-9), 1.19 (2H, m, H-3′b), 1.18 (2H, m, H-5′b), 0.97 (3H, s, H-18), 0.71 (3H, s, H-20) ppm. *ABX spin system. ^13^C-NMR (75 MHz, CDCl_3_) *δ* = 180.4 (C-1′), 172.7 (C-16), 171.0 (C-19OC̲OCH_3_), 170.6 (C-3OC̲OCH_3_), 150.5 (C-14), 146.6 (C-8), 131.5 (C-13), 107.9 (C-17), 79.7 (C-3), 70.6 (C-15), 64.8 (C-19), 56.7 (C-12), 55.5 (C-5), 53.1 (C-9), 52.6 (C-2′), 41.4 (C-4), 39.3 (C-10), 38.4 (C-7), 37.0 (C-1), 32.7 (C-3′), 25.6 (C-5′), 25.4 (C-11), 25.0 (C-4′), 24.9 (C-6), 24.3 (C-2), 22.8 (C-18), 21.2 (C-3OCOC̲H_3_), 21.1 (C-19OCOC̲H_3_), 14.8 (C-20) ppm. ESIMS *m*/*z* 590 [M + H]^+^.

##### (12*S*)-*N*-(2-Morpholinoethyl)hydrazinecarbothioamide-3,19-diacetoxy-14-deoxy-andrographolide (16)

Obtained from the reaction of compound 2 (100 mg, 0.21 mmol) with 4-[2-(4-morphylinyl)ethyl]-3-thiosemicarbazide (51 mg, 0.25 mmol). The residue was purified by column chromatography to afford compound 16 (32 mg, 25%) as a white amorphous powder. ^1^H-NMR (300 MHz, CDCl_3_) *δ* = 7.32 (1H, br s, H-14), 4.86 (1H, s, H-17a), 4.86* (2H, br s, H-15), 4.61 (1H, s, H-17b), 4.51 (1H, m, H-3), 4.28 (1H, d, *J* = 11.7 Hz, H-19a), 4.04 (1H, d, *J* = 11.7 Hz, H-19b), 3.75–3.57 (3H, m, H-12 and H-2′), 3.67 (4H, t, *J* = 6.2 Hz, H-5′), 2.52 (2H, t, *J* = 6.2 Hz, H-4′), 2.48–2.41 (4H, m, H-3′), 1.99 (6H, s, H-3OCOCH_3_ and H-19OCOCH_3_), 1.33 (1H, d, *J* = 11.3 Hz, H-9), 0.94 (3H, s, H-18), 0.67 (3H, s, H-20) ppm. *ABX spin system. ^13^C-NMR (75 MHz, CDCl_3_) *δ* = 180.4 (C-1′), 172.7 (C-16), 170.8 (C-19OC̲OCH_3_), 170.5 (C-3OC̲OCH_3_), 150.1 (C-14), 146.7 (C-8), 131.5 (C-13), 107.7 (C-17), 79.6 (C-3), 70.6 (C-15), 67.0 (C-5′), 64.6 (C-19), 56.7 (C-3′), 56.2 (C-12), 53.3 (C-4′), 55.2 (C-5), 52.8 (C-9), 41.2 (C-4), 40.3 (C-2′), 39.1 (C-10), 38.2 (C-7), 36.7 (C-1), 25.5 (C-11), 24.7 (C-6), 24.1 (C-2), 22.6 (C-18), 21.1 (C-3OCOC̲H_3_), 21.0 (C-19OCOC̲H_3_), 14.6 (C-20) ppm. ESIMS *m*/*z* 621 [M + H]^+^.

##### (12*S*)-*N*-Phenylhydrazinecarbothioamide-3,19-diacetoxy-14-deoxy-andrographolide (17)

Obtained from the reaction of compound 2 (130 mg, 0.27 mmol) with 4-phenylthiosemicarbazide (55 mg, 0.33 mmol). The residue was purified by column chromatography to afford compound 17 (118 mg, 74%) as a dark purple amorphous powder. ^1^H-NMR (300 MHz, CDCl_3_) *δ* = 7.62 (2H, d, *J* = 7.6 Hz, H-3′), 7.38 (1H, s, H-14), 7.35 (2H, t, *J* = 7.9 Hz, H-4′), 7.21 (1H, m, H-5′), 4.95 (1H, s, H-17a), 4.88* (2H, br s, H-15), 4.69 (1H, s, H-17b), 4.56 (1H, dd, *J* = 11.1, 5.3 Hz, H-3), 4.33 (1H, d, *J* = 11.8 Hz, H-19a), 4.09 (1H, d, *J* = 11.8 Hz, H-19b), 3.88 (1H, br d, *J* = 8.7 Hz, H-12), 2.03 (6H, s, H-3OCOCH_3_ and H-19OCOCH_3_), 1.42 (1H, d, *J* = 10.8 Hz, H-9), 0.99 (3H, s, H-18), 0.73 (3H, s, H-20) ppm. *ABX spin system. ^13^C-NMR (75 MHz, CDCl_3_) *δ* = 180.3 (C-1′), 172.6 (C-16), 170.9 (C-19OC̲OCH_3_), 170.6 (C-3OC̲OCH_3_), 150.5 (C-14), 146.6 (C-8), 137.8 (C-2′), 131.3 (C-13), 128.7 (C-4′), 125.8 (C-5′), 123.7 (C-3′), 107.9 (C-17), 79.6 (C-3), 70.6 (C-15), 64.6 (C-19), 56.8 (C-12), 55.3 (C-5), 53.1 (C-9), 41.3 (C-4), 39.2 (C-10), 38.3 (C-7), 36.8 (C-1), 25.3 (C-11), 24.7 (C-6), 24.2 (C-2), 22.6 (C-18), 21.1 (C-3OCOC̲H_3_), 21.0 (C-19OCOC̲H_3_), 14.6 (C-20) ppm. ESIMS *m*/*z* 584 [M + H]^+^.

##### (12*S*)-*N*-(*o*-Tolyl)hydrazinecarbothioamide-3,19-diacetoxy-14-deoxy-andrographolide (18)

Obtained from the reaction of compound 2 (100 mg, 0.21 mmol) with 4-(2-methylphenyl)-3-thiosemicarbazide (46 mg, 0.25 mmol). The residue was purified by column chromatography to afford compound 18 (51 mg, 41%) as a yellow amorphous powder. ^1^H-NMR (300 MHz, CDCl_3_) *δ* = 7.50 (1H, m, H-6′), 7.36 (1H, br s, H-14), 7.20 (3H, m, H-5′, H-4′ and H-3′), 4.91 (1H, s, H-17a), 4.84* (2H, br s, H-15), 4.67 (1H, s, H-17b), 4.55 (1H, dd, *J* = 10.9, 5.4 Hz, H-3), 4.32 (1H, d, *J* = 11.8 Hz, H-19a), 4.07 (1H, d, *J* = 11.8 Hz, H-19b), 3.93–3.83 (1H, m, H-12), 2.25 (3H, s, H-8′), 2.02 (6H, s, H-3OCOCH_3_ and H-19OCOCH_3_), 1.40 (1H, d, *J* = 11.0 Hz, H-9), 0.96 (3H, s, H-18), 0.70 (3H, s, H-20) ppm. *ABX spin system. ^13^C-NMR (75 MHz, CDCl_3_) *δ* = 181.3 (C-1′), 172.7 (C-16), 170.9 (C-19OC̲OCH_3_), 170.6 (C-3OC̲OCH_3_), 150.3 (C-14), 146.5 (C-8), 136.4 (C-7′), 133.9 (C-2′), 131.5 (C-13), 130.6 (C-4′), 127.2 (C-6′), 126.4 (C-5′ and C-3′), 107.9 (C-17), 79.6 (C-3), 70.6 (C-15), 64.7 (C-19), 56.4 (C-12), 55.3 (C-5), 52.9 (C-9), 41.3 (C-4), 39.2 (C-10), 38.3 (C-7), 36.8 (C-1), 25.5 (C-11), 24.8 (C-6), 24.2 (C-2), 22.6 (C-18), 21.1 (C-3OCOC̲H_3_ and C-19OCOC̲H_3_), 18.0 (C-6′OC̲H_3_), 14.6 (C-20) ppm. ESIMS *m*/*z* 598 [M + H]^+^.

##### (12*S*)-*N*-(*p*-Tolyl)hydrazinecarbothioamide-3,19-diacetoxy-14-deoxy-andrographolide (19)

Obtained from the reaction of compound 2 (100 mg, 0.21 mmol) with 4-(4-methylphenyl)-3-thiosemicarbazide (46 mg, 0.25 mmol). The residue was purified by column chromatography to afford compound 19 (110 mg, 88%) as a brown amorphous powder. ^1^H-NMR (300 MHz, CDCl_3_) *δ* = 7.42 (2H, d, *J* = 8.0 Hz, H-3′), 7.35 (1H, br s, H-14), 7.12 (2H, d, *J* = 8.0 Hz, H-4′), 4.91 (1H, s, H-17a), 4.82* (2H, br s, H-15), 4.67 (1H, s, H-17b), 4.54 (1H, dd, *J* = 11.0, 5.4 Hz, H-3), 4.31 (1H, d, *J* = 11.8 Hz, H-19a), 4.06 (1H, d, *J* = 11.8 Hz, H-19b), 3.85 (1H, br d, *J* = 8.1 Hz, H-12), 2.31 (3H, s, H-6′), 2.00 (6H, s, H-3OCOCH_3_ and H-19OCOCH_3_), 1.40 (1H, d, *J* = 10.7 Hz, H-9), 0.96 (3H, s, H-18), 0.70 (3H, s, H-20) ppm. *ABX spin system. ^13^C-NMR (75 MHz, CDCl_3_) *δ* = 180.3 (C-1′), 172.8 (C-16), 170.9 (C-19OC̲OCH_3_), 170.6 (C-3OC̲OCH_3_), 150.6 (C-14), 146.5 (C-8), 135.5 (C-2′), 135.3 (C-5′), 131.4 (C-13), 129.2 (C-4′), 124.0 (C-3′), 107.0 (C-17), 79.7 (C-3), 70.6 (C-15), 64.7 (C-19), 56.5 (C-12), 55.2 (C-5), 53.0 (C-9), 41.3 (C-4), 39.2 (C-10), 38.2 (C-7), 36.8 (C-1), 25.4 (C-11), 24.8 (C-6), 24.2 (C-2), 22.3 (C-18), 21.2 (C-3OCOC̲H_3_), 21.1 (C-19OCOC̲H_3_), 21.0 (C-6′), 14.6 (C-20) ppm. ESIMS *m*/*z* 598 [M + H]^+^.

##### (12*S*)-*N*-(4-Ethylphenyl)hydrazinecarbothioamide-3,19-diacetoxy-14-deoxy-andrographolide (20)

Obtained from the reaction of compound 2 (100 mg, 0.21 mmol) with 4-(4-ethylphenyl)-3-thiosemicarbazide (49 mg, 0.25 mmol). The residue was purified by column chromatography to afford compound 20 (112 mg, 88%) as a brown amorphous powder. ^1^H-NMR (300 MHz, CDCl_3_) *δ* = 7.45 (2H, d, *J* = 8.0 Hz, H-3′), 7.36 (1H, br s, H-14), 7.14 (2H, d, *J* = 8.0 Hz, H-4′), 4.90 (1H, s, H-17a), 4.82* (2H, br s, H-15), 4.67 (1H, s, H-17b), 4.54 (1H, dd, *J* = 11.0, 5.3 Hz, H-3), 4.31 (1H, d, *J* = 11.7 Hz, H-19a), 4.05 (1H, d, *J* = 11.7 Hz, H-19b), 3.81 (1H, br d, *J* = 9.7 Hz, H-12), 2.60 (2H, q, *J* = 7.6 Hz, H-6′), 2.00 (6H, s, H-3OCOCH_3_ and H-19OCOCH_3_), 1.39 (1H, d, *J* = 10.7 Hz, H-9), 1.20 (3H, t, *J* = 7.6 Hz, H-7′), 0.96 (3H, s, H-18), 0.69 (3H, s, H-20) ppm. *ABX spin system. ^13^C-NMR (75 MHz, CDCl_3_) *δ* = 180.2 (C-1′), 172.9 (C-16), 170.9 (C-19OC̲OCH_3_), 170.6 (C-3OC̲OCH_3_), 150.6 (C-14), 146.5 (C-8), 141.8 (C-2′), 135.5 (C-5′), 131.4 (C-13), 128.0 (C-4′), 123.9 (C-3′), 107.0 (C-17), 79.7 (C-3), 70.6 (C-15), 64.7 (C-19), 56.5 (C-12), 55.2 (C-5), 52.9 (C-9), 41.3 (C-4), 39.2 (C-10), 38.2 (C-7), 36.7 (C-1), 28.4 (C-6′), 25.3 (C-11), 24.8 (C-6), 24.2 (C-2), 22.3 (C-18), 21.2 (C-3OCOC̲H_3_), 21.1 (C-19OCOC̲H_3_), 15.2 (C-7′), 14.6 (C-20) ppm. ESIMS *m*/*z* 612 [M + H]^+^.

##### (12*S*)-*N*-(4-Nitrophenyl)hydrazinecarbothioamide-3,19-diacetoxy-14-deoxy-andrographolide (21)

Obtained from the reaction of compound 2 (100 mg, 0.21 mmol) with 4-(4-nitrophenyl)-3-thiosemicarbazide (53 mg, 0.25 mmol). The residue was purified by column chromatography to afford compound 21 (107 mg, 81%) as an orange amorphous powder. ^1^H-NMR (300 MHz, CDCl_3_) *δ* = 8.21 (2H, d, *J* = 9.2 Hz, H-4′), 8.04 (2H, d, *J* = 9.2 Hz, H-3′), 7.42 (1H, br s, H-14), 4.96 (1H, s, H-17a), 4.90* (2H, br s, H-15), 4.68 (1H, s, H-17b), 4.56 (1H, dd, *J* = 11.1, 5.2 Hz, H-3), 4.32 (1H, d, *J* = 11.8 Hz, H-19a), 4.09 (1H, d, *J* = 11.8 Hz, H-19b), 3.94–3.85 (1H, m, H-12), 2.03 (6H, s, H-3OCOCH_3_ and H-19OCOCH_3_), 1.44 (1H, d, *J* = 11.3 Hz, H-9), 0.99 (3H, s, H-18), 0.74 (3H, s, H-20) ppm. *ABX spin system. ^13^C-NMR (75 MHz, CDCl_3_) *δ* = 179.9 (C-1′), 172.8 (C-16), 170.9 (C-19OC̲OCH_3_), 170.6 (C-3OC̲OCH_3_), 150.7 (C-14), 146.6 (C-8), 144.2 (C-2′), 143.9 (C-5′), 131.5 (C-13), 124.7 (C-3′), 121.8 (C-4′), 107.9 (C-17), 79.6 (C-3), 70.7 (C-15), 64.6 (C-19), 56.9 (C-12), 55.4 (C-5), 53.1 (C-9), 41.3 (C-4), 39.3 (C-10), 38.3 (C-7), 36.9 (C-1), 25.2 (C-11), 24.8 (C-6), 24.2 (C-2), 22.6 (C-18), 21.2 (C-3OCOC̲H_3_), 21.1 (C-19OCOC̲H_3_), 14.7 (C-20) ppm. ESIMS *m*/*z* 629 [M + H]^+^.

##### (12*S*)-*N*-(2-Fluorophenyl)hydrazinecarbothioamide-3,19-diacetoxy-14-deoxy-andrographolide (22)

Obtained from the reaction of compound 2 (120.0 mg, 0.25 mmol) with 4-(2-fluorophenyl)-3-thiosemicarbazide (56 mg, 0.30 mmol). The residue was purified by column chromatography to afford compound 22 (121 mg, 80%) as a yellow amorphous powder. ^1^H-NMR (300 MHz, CDCl_3_) *δ* = 8.27 (1H, m, H-3′), 7.39 (1H, br s, H-14), 7.17–7.05 (2H, m, H-4′ and H-5′), 4.92 (1H, s, H-17a), 4.88* (2H, br s, H-15), 4.69 (1H, s, H-17b), 4.55 (1H, dd, *J* = 10.7, 5.2 Hz, H-3), 4.31 (1H, d, *J* = 11.8 Hz, H-19a), 4.07 (1H, d, *J* = 11.8 Hz, H-19b), 3.91–3.80 (1H, m, H-12), 2.01 (6H, s, H-3OCOCH_3_ and H-19OCOCH_3_), 1.41 (1H, d, *J* = 10.5 Hz, H-9), 0.97 (3H, s, H-18), 0.71 (3H, s, H-20) ppm. *ABX spin system. ^13^C-NMR (75 MHz, CDCl_3_) *δ* = 180.5 (C-1′), 172.8 (C-16), 170.9 (C-19OC̲OCH_3_), 170.7 (C-3OC̲OCH_3_), 156.7 (C-6′), 150.3 (C-14), 146.6 (C-8), 131.5 (C-13), 126.4 (C-4′), 126.2 (C-2′), 125.8 (C-3′), 123.9 (C-5′), 107.9 (C-17), 79.7 (C-3), 70.8 (C-15), 64.7 (C-19), 56.5 (C-12), 55.3 (C-5), 53.0 (C-9), 41.3 (C-4), 39.1 (C-10), 38.2 (C-7), 36.8 (C-1), 25.6 (C-11), 24.8 (C-6), 24.2 (C-2), 22.3 (C-18), 21.2 (C-3OCOC̲H_3_), 21.1 (C-19OCOC̲H_3_), 14.6 (C-20) ppm. ESIMS *m*/*z* 602 [M + H]^+^.

##### (12*S*)-*N*-Benzylhydrazinecarbothioamide-3,19-diacetoxy-14-deoxy-andrographolide (23)

Obtained from the reaction of compound 2 (100 mg, 0.21 mmol) with 4-benzyl-3-thiosemicarbazide (46 mg, 0.25 mmol). The residue was purified by column chromatography to afford compound 23 (67 mg, 54%) as a light brown amorphous powder. ^1^H-NMR (300 MHz, CDCl_3_) *δ* = 7.37–7.23 (6H, m, H-14 and H-4′ to H-6′), 4.91–4.68 (3H, m, H-17a and H-2′), 4.82 (2H, br s, H-15), 4.53 (1H, s, H-17b), 4.57–4.48 (1H, m, H-3), 4.30 (1H, d, *J* = 11.8 Hz, H-19a), 4.05 (1H, d, *J* = 11.8 Hz, H-19b), 3.77–3.67 (1H, m, H-12), 2.00 (6H, s, H-3OCOCH_3_ and H-19OCOCH_3_), 1.33 (1H, d, *J* = 10.8 Hz, H-9), 0.96 (3H, s, H-18), 0.65 (3H, s, H-20) ppm. *ABX spin system. ^13^C-NMR (75 MHz, CDCl_3_) *δ* = 181.9 (C-1′), 172.7 (C-16), 170.9 (C-19OC̲OCH_3_), 170.6 (C-3OC̲OCH_3_), 150.2 (C-14), 146.5 (C-8), 137.8 (C-3′), 131.5 (C-13), 128.7 (C-5′), 127.8 (C-4′), 127.6 (C-6′), 107.8 (C-17), 79.6 (C-3), 70.6 (C-15), 64.7 (C-19), 56.6 (C-12), 55.2 (C-5), 53.0 (C-9), 47.8 (C-2′), 41.2 (C-4), 39.1 (C-10), 38.2 (C-7), 36.7 (C-1), 25.4 (C-11), 24.7 (C-6), 24.1 (C-2), 22.4 (C-18), 21.2 (C-3OCOC̲H_3_), 21.1 (C-19OCOC̲H_3_), 14.6 (C-20) ppm. ESIMS *m*/*z* 598 [M + H]^+^.

##### (12*S*)-*N*-(Naphthalen-1-yl)hydrazinecarbothioamide-3,19-diacetoxy-14-deoxy-andrographolide (24)

Obtained from the reaction of compound 2 (100 mg, 0.21 mmol) with 4-(1-naftil)-3-thiosemicarbazide (55 mg, 0.25 mmol). The residue was purified by column chromatography to afford compound 24 (108 mg, 81%) as a dark brown amorphous powder. ^1^H-NMR (300 MHz, CDCl_3_) *δ* = 8.00–7.70 (4H, m, H-3′, H-5′, H-10′ and H-11′), 7.56–7.45 (3H, m, H-4′, H-8′ and H-9′), 7.37 (1H, br s, H-14), 4.94 (1H, s, H-17a), 4.83* (2H, br s, H-15), 4.73 (1H, s, H-17b), 4.56 (1H, dd, *J* = 11.0, 5.2 Hz, H-3), 4.32 (1H, d, *J* = 11.8 Hz, H-19a), 4.07 (1H, d, *J* = 11.8 Hz, H-19b), 3.96 (1H, br s, H-12), 2.03 (6H, s, H-3OCOCH_3_ and H-19OCOCH_3_), 1.44 (1H, d, *J* = 10.0 Hz, H-9), 0.97 (3H, s, H-18), 0.71 (3H, s, H-20) ppm. *ABX spin system. ^13^C-NMR (75 MHz, CDCl_3_) *δ* = 185.0 (C-1′), 172.8 (C-16), 170.9 (C-19OC̲OCH_3_), 170.6 (C-3OC̲OCH_3_), 150.1 (C-14), 146.6 (C-8), 134.2 (C-2′), 133.7 (C-6′), 131.5 (C-13), 129.8 (C-7′), 128.6 (C-11′), 127.5 (C-10′), 126.6 (C-9′), 126.3 (C-4′ and C-8′), 125.4 (C-5′), 122.0 (C-3′), 108.0 (C-17), 79.7 (C-3), 70.6 (C-15), 64.7 (C-19), 56.7 (C-12), 55.3 (C-5), 53.0 (C-9), 41.3 (C-4), 39.2 (C-10), 38.3 (C-7), 36.6 (C-1), 25.7 (C-11), 24.8 (C-6), 24.2 (C-2), 22.4 (C-18), 21.2 (C-3OCOC̲H_3_), 21.1 (C-19OCOC̲H_3_), 14.6 (C-20) ppm. ESIMS *m*/*z* 634 [M + H]^+^.

##### (12*S*)-*N*-(Furan-2-ylmethyl)hydrazinecarbothioamide-3,19-diacetoxy-14-deoxy-andrographolide (25)

Obtained from the reaction of compound 2 (100 mg, 0.21 mmol) with 4-(2-furfuryl)-3-thiosemicarbazide (43 mg, 0.25 mmol). The residue was purified by column chromatography to afford compound 25 (88 mg, 71%) as a brown amorphous powder. ^1^H-NMR (300 MHz, CDCl_3_) *δ* = 7.35 (1H, dd, *J* = 1.9, 0.9 Hz, H-6′), 7.31 (1H, t, *J* = 1.7 Hz, H-14), 6.31 (1H, d, *J* = 1.9 Hz, H-4′), 6.28 (1H, d, *J* = 0.9 Hz, H-5′), 4.84 (1H, s, H-17a), 4.83* (2H, br s, H-15), 4.56 (1H, s, H-17b), 4.52 (1H, dd, *J* = 10.9, 5.2 Hz, H-3), 4.30 (1H, d, *J* = 11.8 Hz, H-19a), 4.05 (1H, d, *J* = 11.8 Hz, H-19b), 3.78–3.67 (1H, m, H-12), 2.00 (6H, s, H-3OCOCH_3_ and H-19OCOCH_3_), 1.34 (1H, d, *J* = 11.2 Hz, H-9), 0.95 (3H, s, H-18), 0.66 (3H, s, H-20) ppm. *ABX spin system. ^13^C-NMR (75 MHz, CDCl_3_) *δ* = 181.7 (C-1′), 172.9 (C-16), 170.9 (C-19OC̲OCH_3_), 170.6 (C-3OC̲OCH_3_), 150.7 (C-3′), 150.2 (C-14), 146.7 (C-8), 142.6 (C-6′), 131.5 (C-13), 110.7 (C-5′), 108.3 (C-4′), 107.8 (C-17), 79.6 (C-3), 70.6 (C-15), 64.7 (C-19), 56.5 (C-12), 55.3 (C-5), 53.0 (C-9), 41.2 (C-4), 40.8 (C-2′), 39.1 (C-10), 38.2 (C-7), 36.8 (C-1), 25.4 (C-11), 24.7 (C-6), 24.1 (C-2 and C-18), 21.2 (C-3OCOC̲H_3_), 21.0 (C-19OCOC̲H_3_), 14.6 (C-20) ppm. ESIMS *m*/*z* 588 [M + H]^+^.

### 5.3. Cell lines

L5178Y mouse T-lymphoma cells (PAR) (ECACC cat. no. 87111908, U.S. FDA, Silver Spring, MD, U.S.) were transfected with pHa MDR1/A retrovirus, as previously described by Pastan *et al.*^37^ The ABCB1-expressing cell line (MDR) was selected by culturing the infected cells with 60 ng mL^−1^ of colchicine (Sigma-Aldrich Chemie GmbH, Steinheim, Germany). Both cell lines were cultured in McCoy's 5A supplemented with 10% heat-inactivated horse serum, 100 U L^−1^l-glutamine, and 100 mg L^−1^ penicillin–streptomycin mixture, all obtained from Sigma-Aldrich. Cell lines were kept at 37 °C and the maintenance of cultures was performed every 2–3 days.

### 5.4. Antiproliferative assay

The antiproliferative effect of andrographolide (1) and its derivatives (2–24) on parental (PAR) and multidrug-resistant (MDR) mouse T-lymphoma cells was assessed by the thiazolyl blue tetrazolium bromide assay. In 96-well flat-bottomed microtiter plates, 100 μL of medium was pipetted to each well, except for the medium control wells, where 200 μL of medium was pipetted. The compounds were diluted by 2-fold serial dilution (starting with 100 μM) in 100 μL of medium. Then, the cells were prepared in the concentration of 6 × 10^3^ cells in 100 μL of medium, except for the cell control. Doxorubicin was used as a positive control and was also diluted by 2-fold serial dilution (starting with 8.6 μM). After 72 h incubation at 37 °C, 20 μL of MTT (thiazolyl blue tetrazolium bromide; Sigma-Aldrich Chemie GmbH, Steinheim) solution of 5 mg mL^−1^ in phosphate-buffered saline (PBS) was added to each well and incubated for another 4 h, after which 100 μL of 10% SDS (sodium dodecyl sulfate, Sigma) solution was added to each well. Following an overnight incubation period at 37 °C, the optical density (OD) at 540/630 nm was measured with a Multiscan EX ELISA reader (Thermo Labsystems, Cheshire, WA, USA). All experiments were performed in triplicate. The results were expressed as the mean IC_50_ ± SD, and the IC_50_ values were obtained by best-fitting the dose-dependent inhibition curves in GraphPad Prism 5 software.^29^ The percentage of inhibition of cell growth was determined according to the following equation:
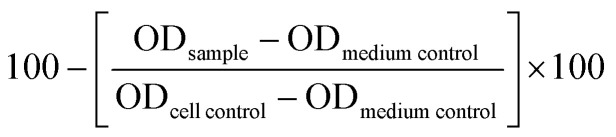


### 5.5. Rhodamine-123 accumulation assay

PAR and MDR mouse T-lymphoma cells were initially adjusted to a density of 2 × 10^6^ cells per mL, resuspended in a serum-free McCoy's 5A medium, and distributed in Eppendorf centrifuge tubes (500 μL aliquots). The andrographolide (1) and its derivatives (2–25) were added at 0.2, 2 and 20 μM, verapamil (positive control, EGIS Pharmaceuticals PLC, Budapest, Hungary) at 20 μM, and DMSO (solvent control) at 2%. After 10 min incubation at room temperature, 10 μL of rhodamine-123 (5.2 μM final concentration) was added and further incubated for 20 min at 37 °C. The samples were washed twice, resuspended in 1 mL of PBS and analyzed by flow cytometry (CyFlow® Space instrument, Partec GmbH, Münster, Germany). Histograms were evaluated regarding mean fluorescence intensity (FL-1), standard deviation and both forward scatter (FSC) and side scatter (SSC) parameters. The fluorescence activity ratio (FAR) was calculated as the quotient between FL-1 of the treated/untreated resistant cell line (L5178Y-MDR) and treated/untreated sensitive cell line (L5178Y-PAR), according to the following equation:
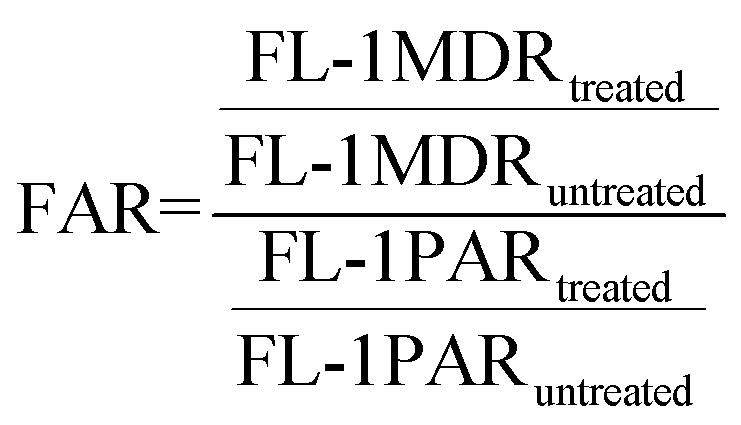


### 5.6. Drug combination assay

Doxorubicin (2 mg mL^−1^, Teva Pharmaceuticals, Budapest, Hungary) was serially diluted horizontally in 100 μL as previously described, starting with 8.6 μM. The resistance modifier was subsequently diluted vertically in 50 μL; the starting concentration was determined based on the IC_50_. After resuspending the cells in culture medium, they were distributed into each well in 50 μL containing 6 × 10^3^ cells, except for the medium control wells, to a final volume of 200 μL per well. The checkerboard plates were incubated for 72 h at 37 °C in a CO_2_ incubator, and at the end of the incubation period, the cell growth was determined by the MTT staining method, as described earlier. Drug interactions were assessed using the Calcusyn software. Each dose–response curve (for individual agents as well as combinations) was fitted to a linear model using the median effect equation to obtain the median effect value (thus corresponding to the IC_50_) and slope (*m*). Goodness-of-fit was assessed using the linear correlation coefficient, *r*, and only data from analysis with *r* > 0.90 were considered. The extent of interaction between test compounds was expressed using the combination index (CI). A CI value close to 1 indicates additivity; CI < 1 defines synergism and CI > 1 is related to antagonism.

### 5.7. P-gp ATPase activity assay

According to the manufacturer's instructions, the Pgp-Glo™ Assay Systems Promega kit was used to measure the P-glycoprotein ATPase activity.^33^ Pgp-Glo™ assay buffer was mixed with 20 μL of recombinant human P-gp membranes (1.25 mg mL^−1^), which express high amounts of human P-gp, for 5 min at 37 °C. Compounds were tested at 25 μM. Verapamil was used as a substrate control (0.5 mM), sodium orthovanadate was used as an inhibitor control (25 M), and DMSO was used as a solvent control. Adding 10 μL of 25 mM MgATP started the process, which was then given 40 minutes of incubation at 37 °C. After stopping the ATPase reaction, the samples were incubated at room temperature for 20 min with 50 μL of ATP Detection Reagent. A CLARIOstar Plus plate reader (BMG Labtech, Ortenberg, Germany) was used to measure the luciferase-generated luminescent signal, at 580 nm, which corresponds to the remaining unmetabolized ATP, and is expressed as relative light units (RLU). The difference between the luminescent signals from the Na_3_VO_4_-treated sample (RLU_Na_3_VO_4__) and untreated (NT) sample (RLU_NT_) gives the basal P-gp ATPase activity (ΔRLU_basal_). Based on the difference in luminescence measured between the Na_3_VO_4_-treated sample and the test compound-treated samples (RLU_TC_), the relative ATPase activity of the test compounds (ΔRLU_TC_) was determined according to the following equations:ΔRLU_TC_ = RLU_Na_3_VO_4__ − RLU_TC_ΔRLU_TC_ > ΔRLU_basal_: the compound is stimulator of P-gp ATPase activity;ΔRLU_TC_ = ΔRLU_basal_: the compound has no effect on P-gp ATPase activity;ΔRLU_TC_ < ΔRLU_basal_: the compound is inhibitor of P-gp ATPase activity.

### 5.8. Physiochemical and pharmacokinetic properties

After generation of SMILES notation, physiochemical and pharmacokinetic parameters were predicted for all derivatives using SwissADME^31,38^ and pkCSM^32,39^ online software.

## Conflicts of interest

There are no conflicts to declare.

## Supplementary Material

MD-015-D3MD00711A-s001

## References

[cit1] Sajid A., Rahman H., Ambudkar S. V. A. (2023). Nat. Rev. Cancer.

[cit2] Kartal-Yandim M., Adan-Gokbulut A., Baran Y. (2016). Crit. Rev. Biotechnol..

[cit3] Bugde P., Biswas R., Merien F., Lu J., Liu D. X., Chen M., Zhou S., Li Y. (2017). Expert Opin. Ther. Targets.

[cit4] Pluchino K. M., Hall M. D., Goldsborough A. S., Callaghan R., Gottesman M. M. (2012). Drug Resistance Updates.

[cit5] Stefan S. M., Wiese M. (2019). Med. Res. Rev..

[cit6] Kumar A., Jaitak V. (2019). Eur. J. Med. Chem..

[cit7] Newman D. J., Cragg G. M. (2020). J. Nat. Prod..

[cit8] Dai Y., Chen S. R., Chai L., Zhao J., Wang Y., Wang Y. (2019). Crit. Rev. Food Sci. Nutr..

[cit9] Burgos R. A., Alarcón P., Quiroga J., Manosalva C., Hancke J. (2020). Molecules.

[cit10] Wang W., Guo W., Li L., Fu Z., Liu W., Gao J., Shu Y., Xu Q., Sun Y., Gu Y. (2016). Biochem. Pharmacol..

[cit11] Liao H. C., Chou Y. J., Lin C. C., Liu S. H., Oswita A., Huang Y. L., Wang Y. L., Syu J. L., Sun C. M., Leu C. M., Lin C. H., Fu S. L. (2019). Biochem. Pharmacol..

[cit12] Chen X., Zhang J., Yuan L., Lay Y., Wong Y. K., Lim T. K., Ong C. S., Lin Q., Wang J., Hua Z. (2017). Molecules.

[cit13] Najar I. A., Sachin B. S., Sharma S. C., Satti N. K., Suri K. A., Johri R. K. (2010). Phytother. Res..

[cit14] Ye L., Wang T., Tang L., Liu W., Yang Z., Zhou J., Zheng Z., Cai Z., Hu M., Liu1 Z. (2011). J. Pharm. Sci..

[cit15] Yang T., Sheng H. H., Feng N. P., Wei H., Wang Z. T., Wang C. H. (2013). J. Pharm. Sci..

[cit16] Reis M. A., Ahmed O. B., Spengler G., Molnar J., Lage H., Ferreira M. J. U. (2016). Phytomedicine.

[cit17] Paterna A., Kincses A., Spengler G., Mulhovo S., Molnár J., Ferreira M. J. U. (2017). Eur. J. Med. Chem..

[cit18] Cardoso D. S. P., Kincses A., Nové M., Spengler G., Mulhovo S., Aires-de-Sousa J., Dos Santos D. J. V. A., Ferreira M. J. U. (2021). Eur. J. Med. Chem..

[cit19] Cardoso D. S. P., Szemerédi N., Spengler G., Mulhovo S., Santos D. J. V. A. Dos, Ferreira M. J. U. (2021). Pharmaceuticals.

[cit20] Raimundo L., Paterna A., Calheiros J., Ribeiro J., Cardoso D. S. P., Piga I., Neto S. J., Hegan D., Glazer P. M., Indraccolo S., Mulhovo S., Costa J. L., Ferreira M. J. U., Saraiva L. (2021). Br. J. Pharmacol..

[cit21] Reis M. A., Matos A. M., Duarte N., Ahmed O. B., Ferreira R. J., Lage H., Ferreira M. J. U. (2020). Front. Pharmacol..

[cit22] Sancha S. A. R., Szemerédi N., Spengler G., Ferreira M. J. U. (2023). Int. J. Mol. Sci..

[cit23] Ferreira R. J., Kincses A., Gajdács M., Spengler G., Dos Santos D. J. V. A., Molnár J., Ferreira M. J. U. (2018). J. Nat. Prod..

[cit24] Gonçalves B. M. F., Cardoso D. S. P., Ferreira M. J. U. (2020). Molecules.

[cit25] Yin H., Dong J., Cai Y., Shi X., Wang H., Liu G., Tang Y., Liu J., Ma L. (2019). Eur. J. Med. Chem..

[cit26] Seelig A., Landwojtowicz E. (2000). Eur. J. Pharm. Sci..

[cit27] Kasemsuk S., Sirion U., Suksen K., Piyachaturawat P., Suksamrarn A., Saeeng R. (2013). Arch. Pharmacal Res..

[cit28] Hall M. D., Handley M. D., Gottesman M. M. (2009). Trends Pharmacol. Sci..

[cit29] Chou T. C. (2006). Pharmacol. Rev..

[cit30] Chou T. C. (2010). Cancer Res..

[cit31] SwissADME, https://www.swissadme.ch/index.php, (accessed 23 June 2023)

[cit32] Pires D. E. V., Blundell T. L., Ascher D. B. (2015). J. Med. Chem..

[cit33] Dongping M., Cali J. J. (2007). Promega Notes.

[cit34] Wiese M., Pajeva I. (2001). Curr. Med. Chem..

[cit35] McDevitt C. A., Callaghan R. (2007). Pharmacol. Ther..

[cit36] Vistoli G. and Pedretti A., in Comprehensive Medicinal Chemistry II, ed. D. B. Taylor and D. J. Triggle, Elsevier Ltd, 2007, vol. 5, pp. 577–602

[cit37] Pastan I., Gottesman M. M., Ueda K., Lovelace E., Rutherford A. V., Willingham M. C. (1988). Proc. Natl. Acad. Sci. U. S. A..

[cit38] Daina A., Michielin O., Zoete V. (2017). Sci. Rep..

[cit39] PkCSM-Pharmacokinetics, https://biosig.lab.uq.edu.au/pkcsm/, (accessed 23 June 2023)

