# Supplementary material for: Nitrogen-containing andrographolide derivatives with multidrug resistance reversal effects in cancer cells
Source: RSC Med Chem. 2024 Feb 26;15(4):1348–61. doi: 10.1039/d3md00711a (PMC11042158; doi:10.1039/d3md00711a)
Supplement: MD-015-D3MD00711A-s001 [file MD-015-D3MD00711A-s001.pdf]

Electronic Supplementary Material (ESI) for RSC Medicinal Chemistry.
This journal is © The Royal Society of Chemistry 2024

Electronic Supplementary Information

Nitrogen-containing andrographolide derivatives with multidrug resistance reversal effects in cancer cells

Joana Ribeiro[a], Nikoletta Szemerédi[b], Bruno M. F. Goncalves[a], Gabriella Spengler[b], Carlos A. M. Afonso[a], Maria-José U. Ferreira[a,*]

[a] Research Institute for Medicines (iMed.ULisboa), Faculty of Pharmacy, Universidade de Lisboa, Av. Prof. Gama Pinto, 1649-003 Lisbon, Portugal
[b] Department of Medical Microbiology, Albert Szent-Györgyi Health Center, Faculty of Medicine, University of Szeged, Semmelweis utca 6, 6725 Szeged, Hungary

*Corresponding Author: Maria-José Ferreira; E-mail: mjuferreira@ff.ulisboa.pt

Table of contents

1. NMR data of parental compound 1 S3

2. Representative ^{1}H and ^{13}C NMR spectra S5

3. Rhodamine-123 accumulation assay (compounds 1 – 25) S15

4. Flow cytometry data S18

5. Combination chemotherapy results S73

6. Physicochemical properties S74

1. NMR data of parental compound 1

White amorphous powder; ^{1}H-NMR (300 MHz, DMSO-d6) δ = 6.62 (1H, *td*, J = 6.8, 1.7 Hz, H-12), 5.70 (1H, *d*, J = 6.1 Hz, H-14-OH), 5.04 (1H, *d*, J = 4.9 Hz, H-3-OH), 4.91 (1H, *br t*, J = 6.1 Hz, H-14), 4.81 (1H, *br s*, H-17a), 4.63 (1H, *br s*, H-17b), 4.39 (1H, *dd*, J = 9.9, 6.1 Hz, H-15a), 4.12 (1H, *dd*, J = 7.5, 2.9 Hz, H-19-OH), 4.03 (1H, *dd*, J = 9.9, 2.1 Hz, H-15b), 3.84 (1H, *dd*, J = 11.0, 2.9 Hz, H-19a), 3.29-3.19 (2H, *m*, H-3 and H-19b), 2.46 (2H, *m*, H-11), 2.32 (2H, *m*, H-7), 1.95 (2H, *m*, H-2), 1.74 (2H, *m*, H-6), 1.63 (2H, *m*, H-1), 1.30 (1H, *m*, H-9), 1.20 (1H, *m*, H-5), 1.08 (3H, *s*, H-18), 0.66 (3H, *s*, H-20) ppm. ^{13}C-NMR (75 MHz, $CDCl_3$) δ = 169.9 (C-16), 147.6 (C-12), 146.3 (C-8), 129.0 (C-13), 108.2 (C-17), 78.4 (C-3), 74.3 (C-15), 64.5 (C-14), 62.6 (C-19), 55.5 (C-9), 54.4 (C-5), 42.3 (C-4), 38.6 (C-10), 37.5 (C-7), 36.5 (C-1), 27.9 (C-2), 23.9 (C-6 and C-11), 23.0 (C-18), 14.7 (C-20) ppm. ESI-MS (positive mode) m/z (rel. Int) 351 [M + H]$^+$. These data are in agreement with the literature.[1,2]

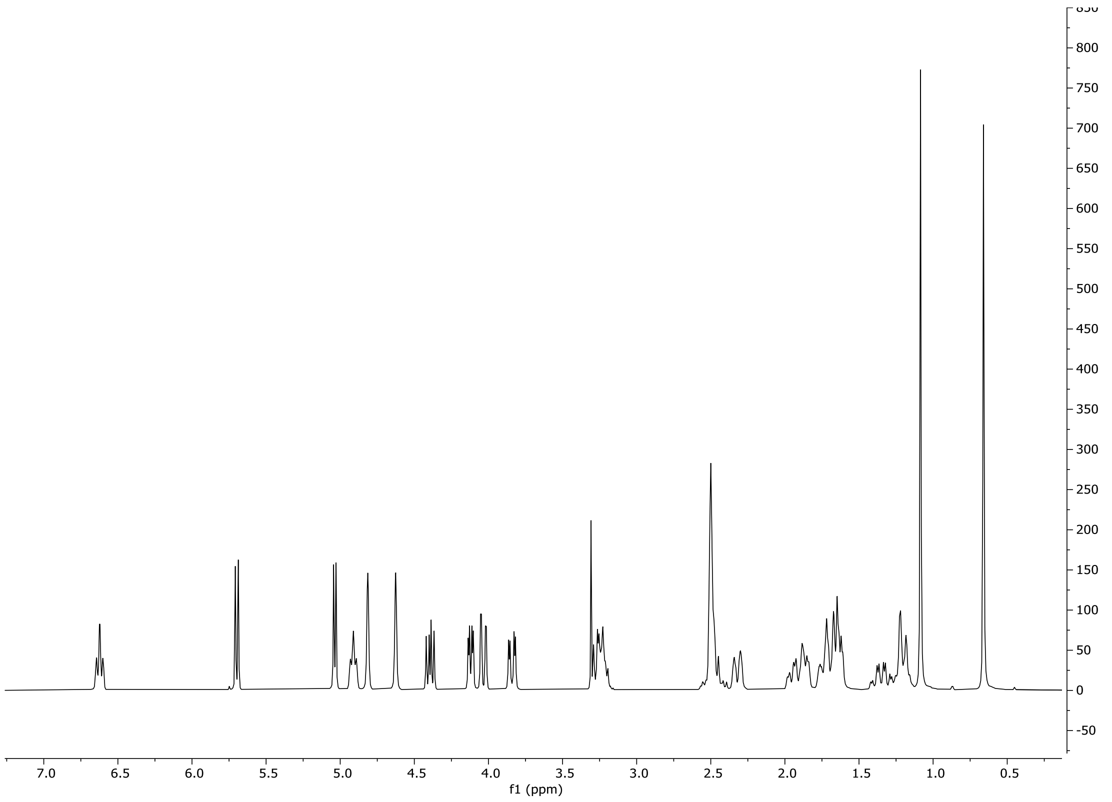

Figure S1: ^{1}H-NMR spectrum of compound **1** (300 MHz, DMSO-d6)

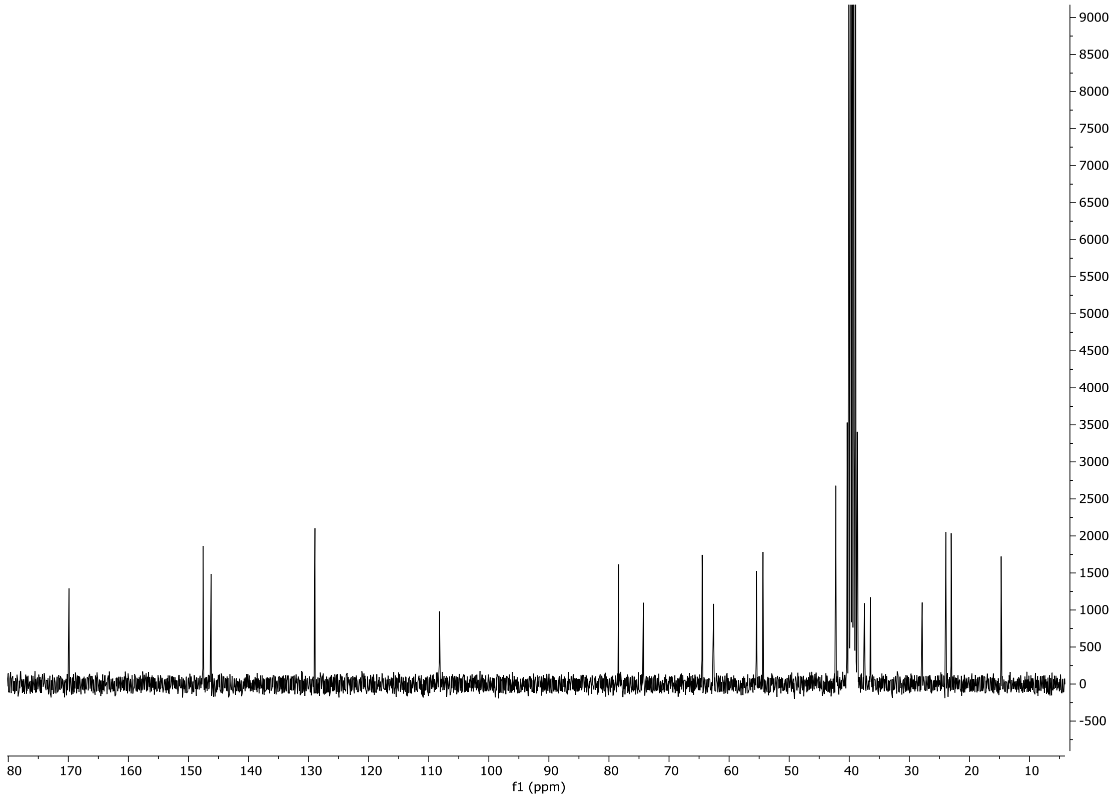

Figure S2: ^{13}C-NMR spectrum of compound **1** (75 MHz, DMSO-d6)

2. Representative 1H and 13C NMR spectra

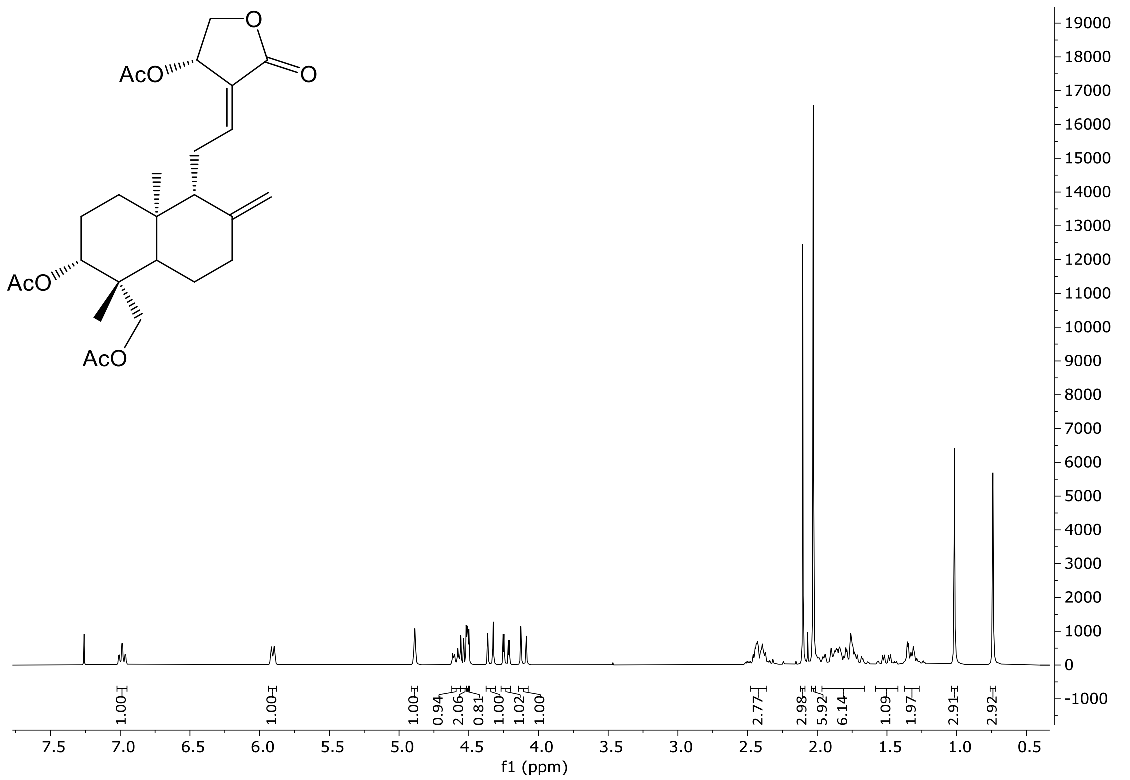

Figure S3: ^{1}H-NMR spectrum of compound **2** (300 MHz, $CDCl_3$)

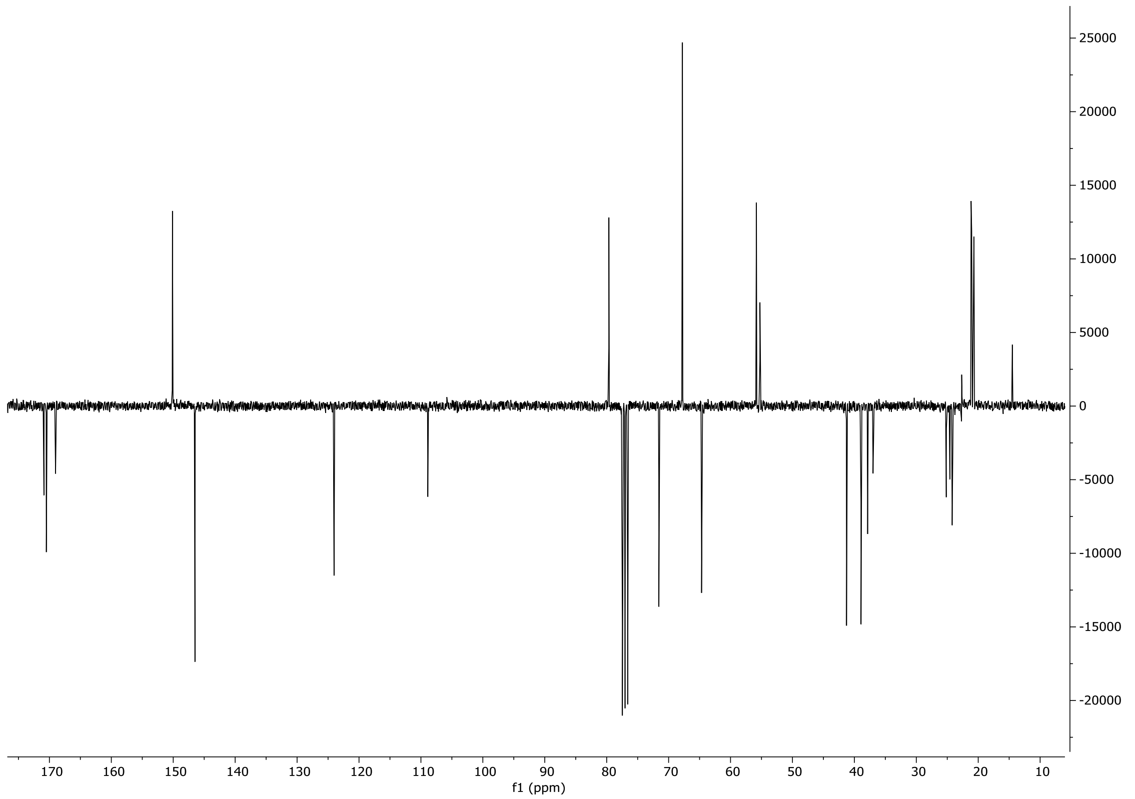

Figure S4: ^{13}C-APT NMR spectrum of compound **2** (75 MHz, $CDCl_3$)

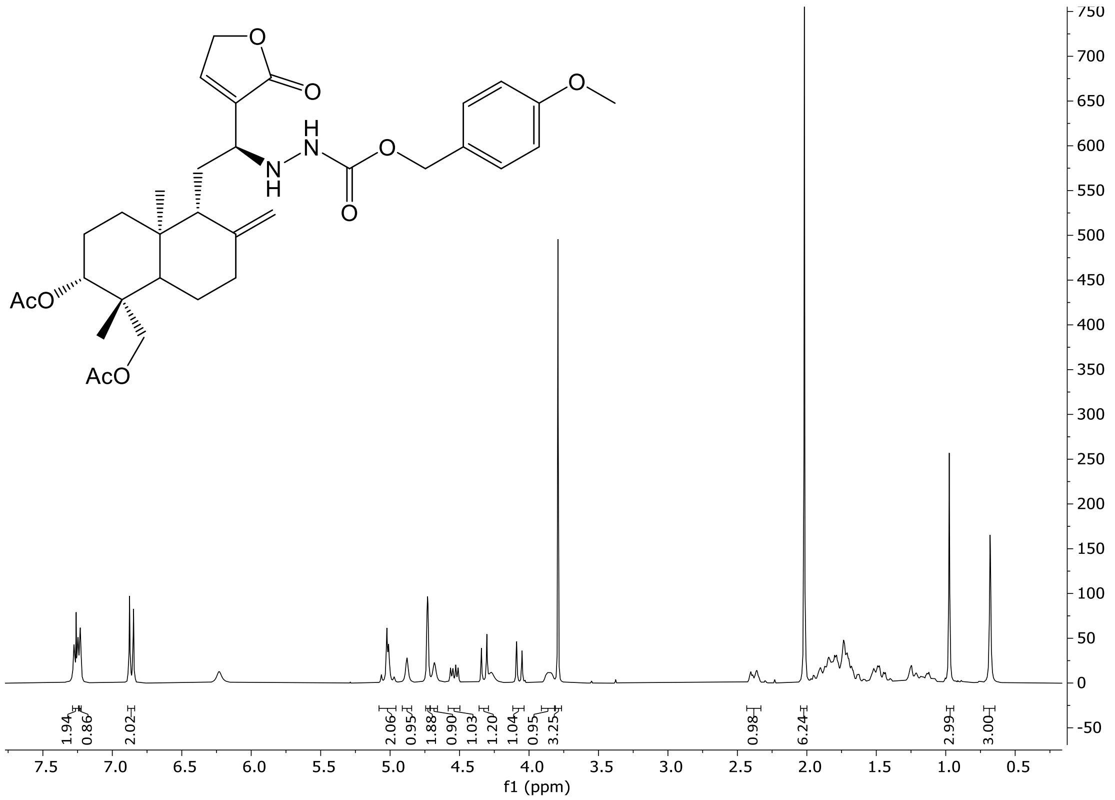

Figure S5: ^{1}H-NMR spectrum of compound **4** (300 MHz, $CDCl_3$)

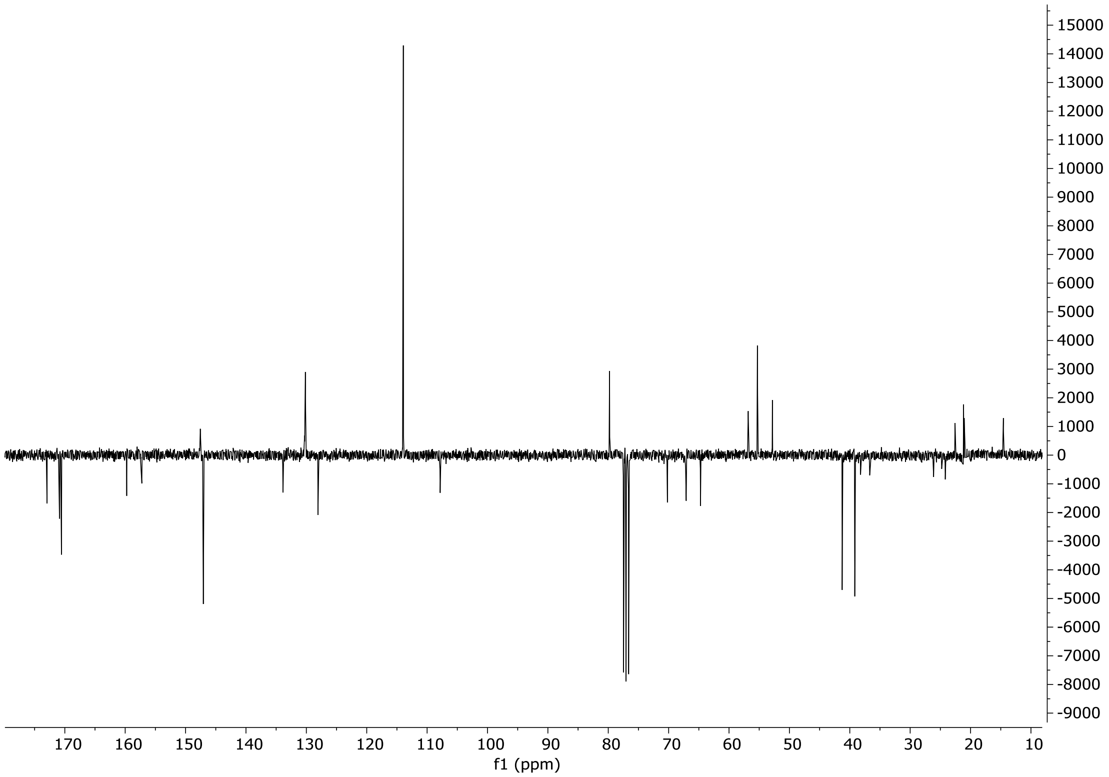

Figure S6: ^{13}C-APT NMR spectrum of compound **4** (75 MHz, $CDCl_3$)

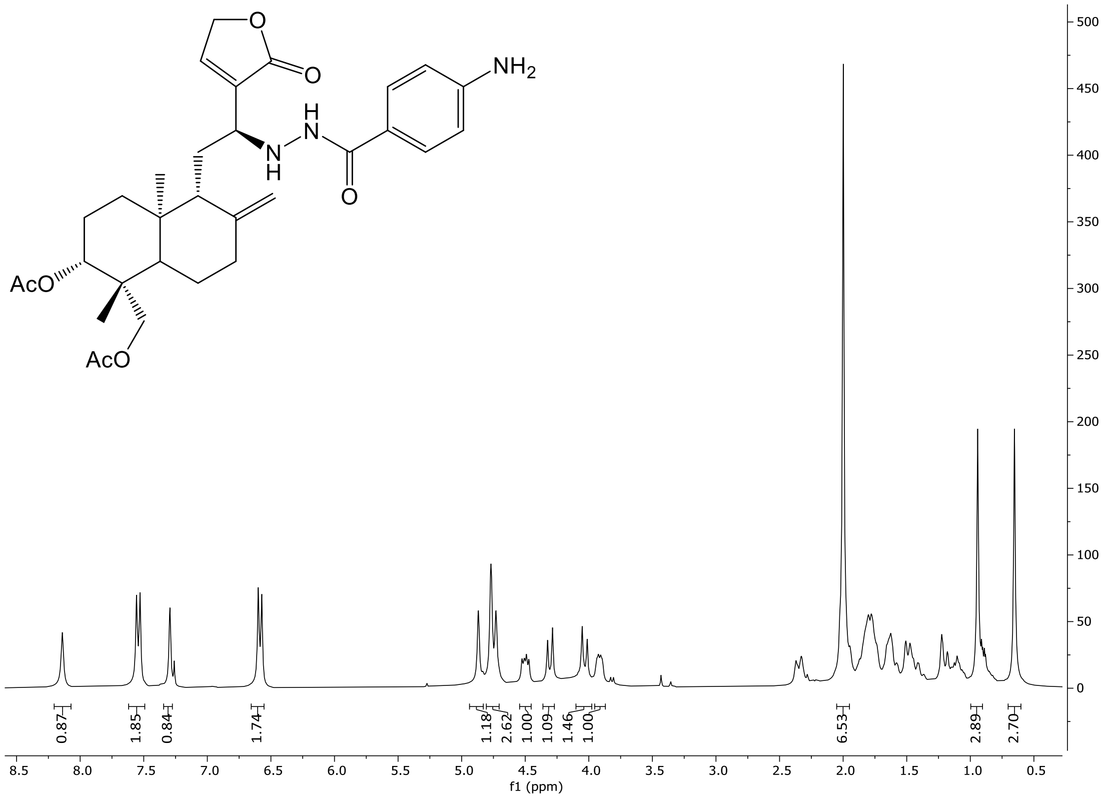

Figure S7: ^{1}H-NMR spectrum of compound **5** (300 MHz, $CDCl_3$)

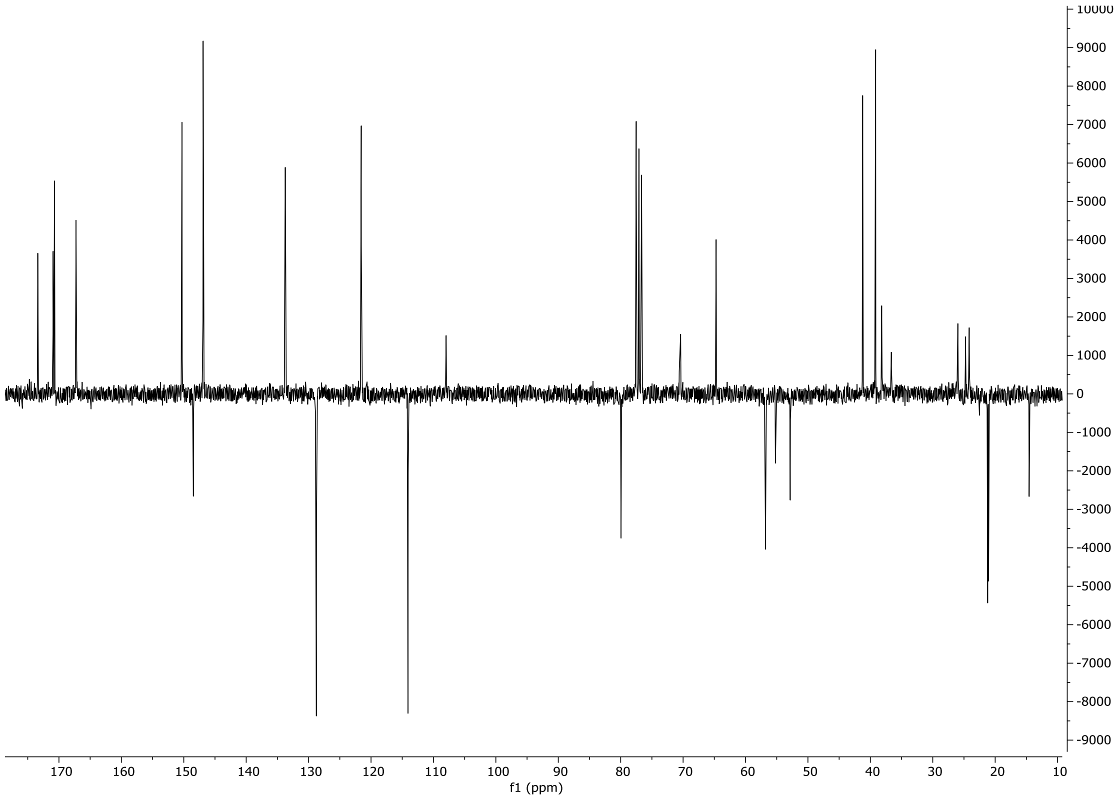

Figure S8: ^{13}C-APT NMR spectrum of compound **5** (75 MHz, $CDCl_3$)

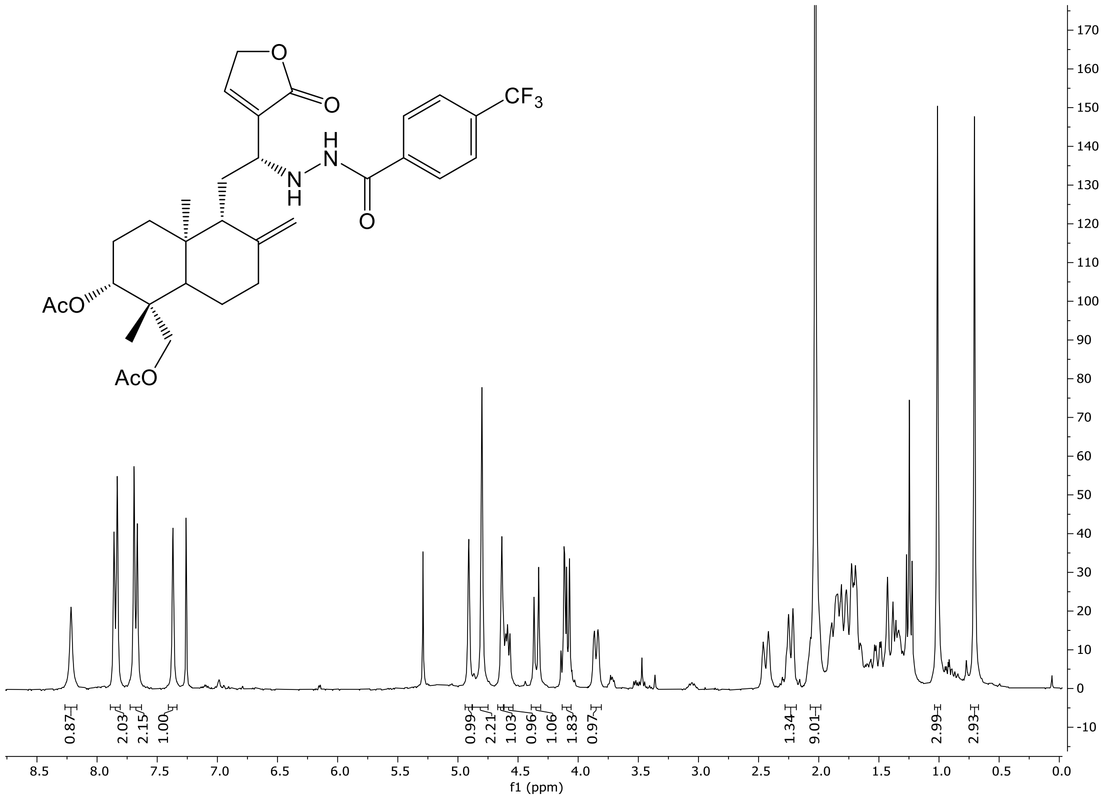

Figure S9: ^{1}H-NMR spectrum of compound **7** (300 MHz, $CDCl_3$)

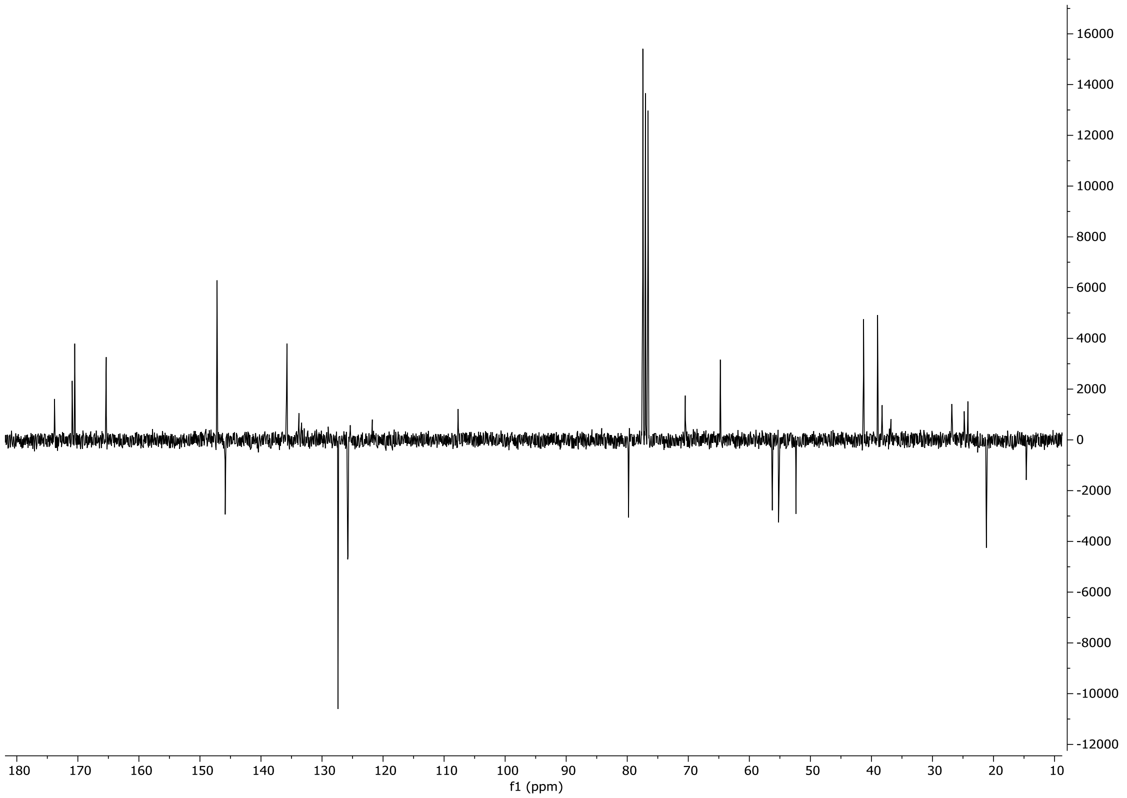

Figure S10: ^{13}C-APT NMR spectrum of compound **7** (75 MHz, $CDCl_3$)

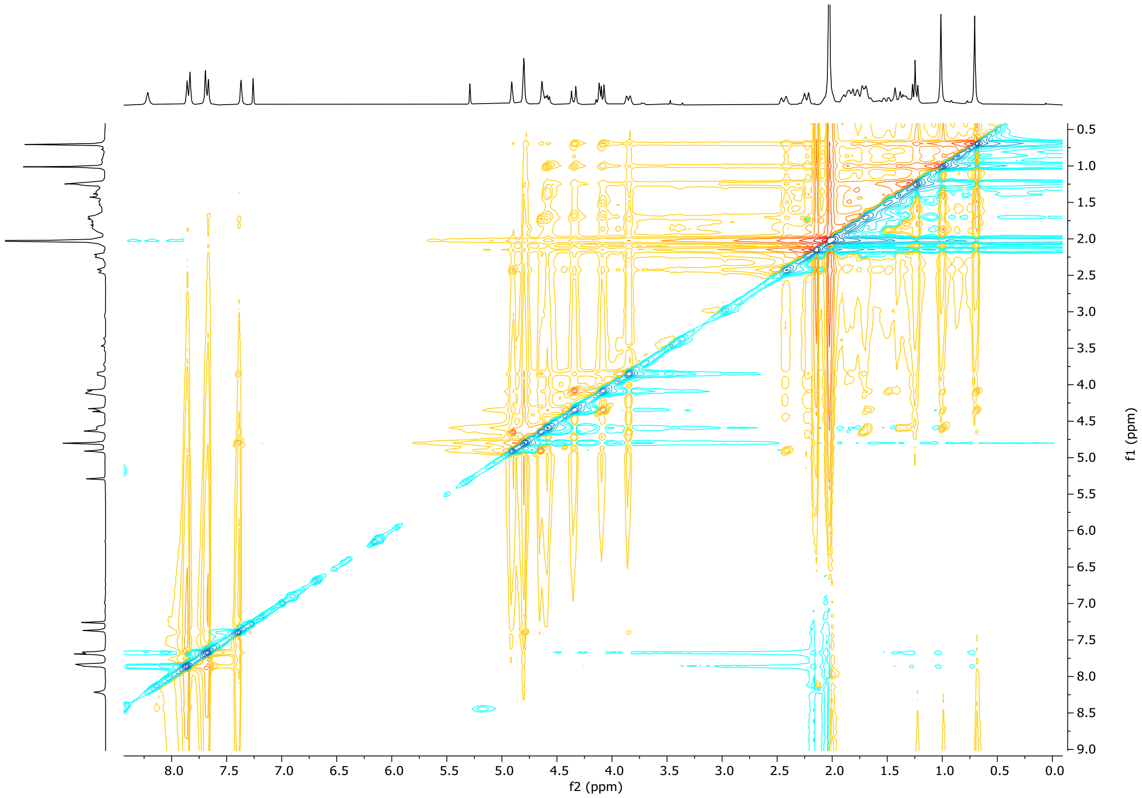

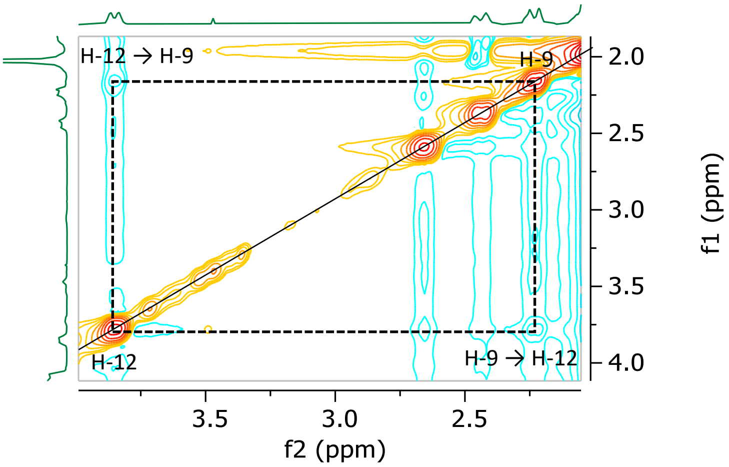

Figure S11: NOESY spectrum and expansion of compound **7** (300 MHz, $CDCl_3$)

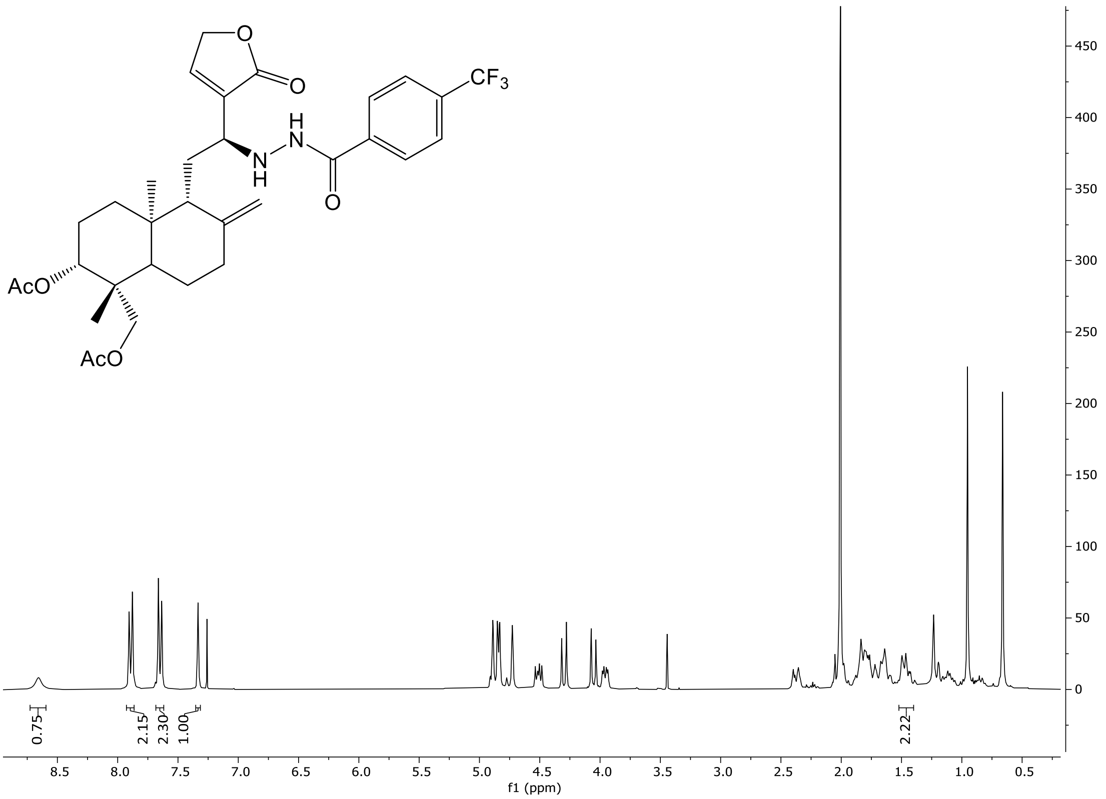

Figure S12: ^{1}H-NMR spectrum of compound **8** (300 MHz, $CDCl_3$)

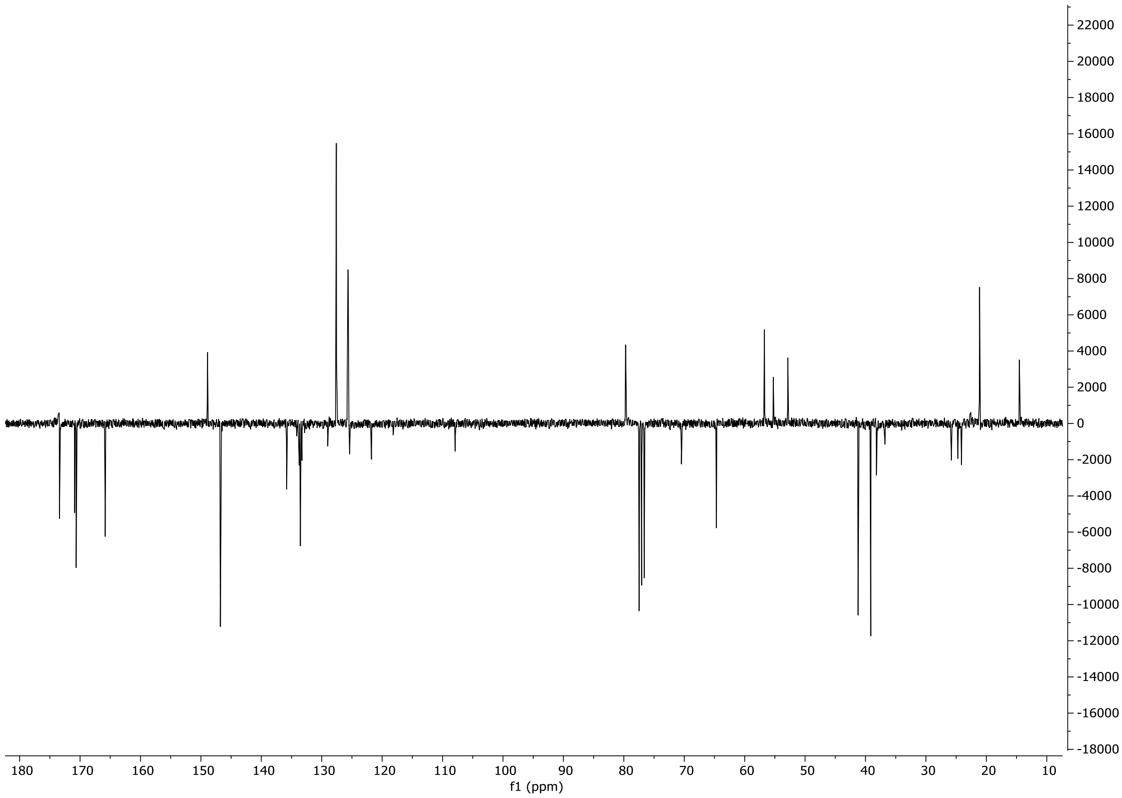

Figure S13: ^{13}C-APT NMR spectrum of compound **8** (75 MHz, $CDCl_3$)

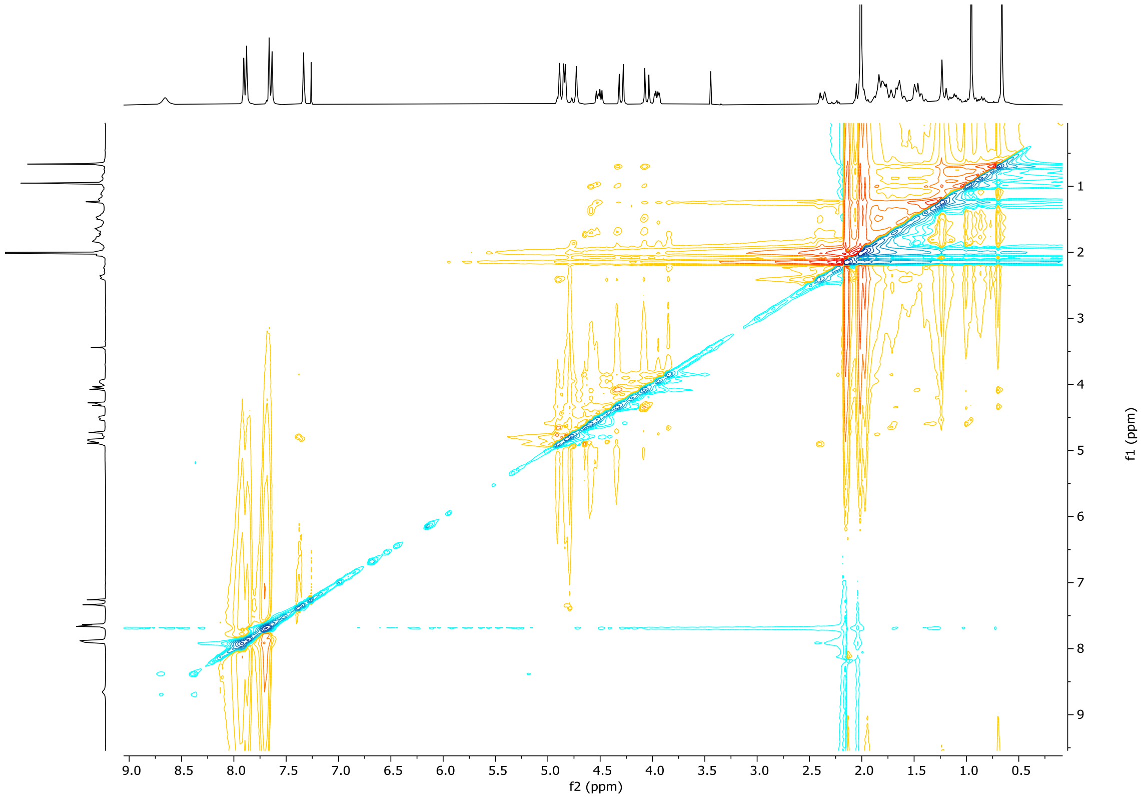

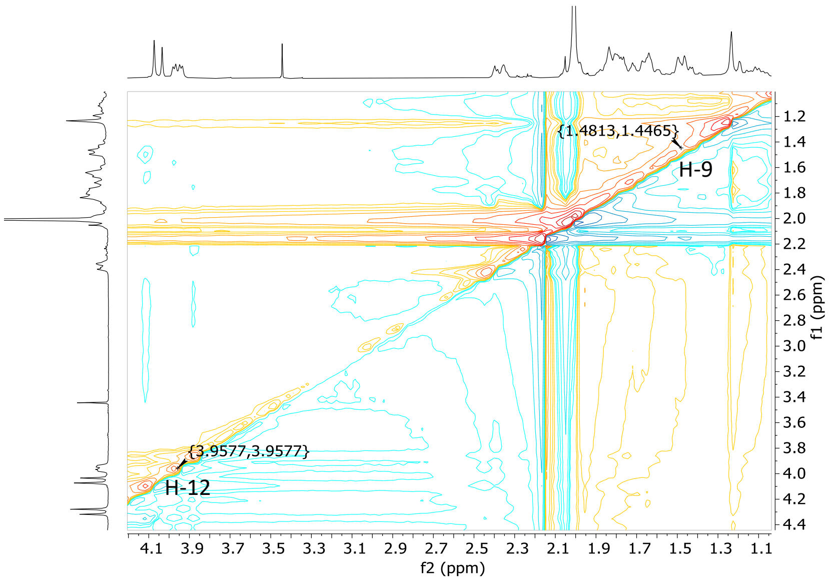

Figure S14: NOESY spectrum and expansion of compound **8** (500 MHz, $CDCl_3$)

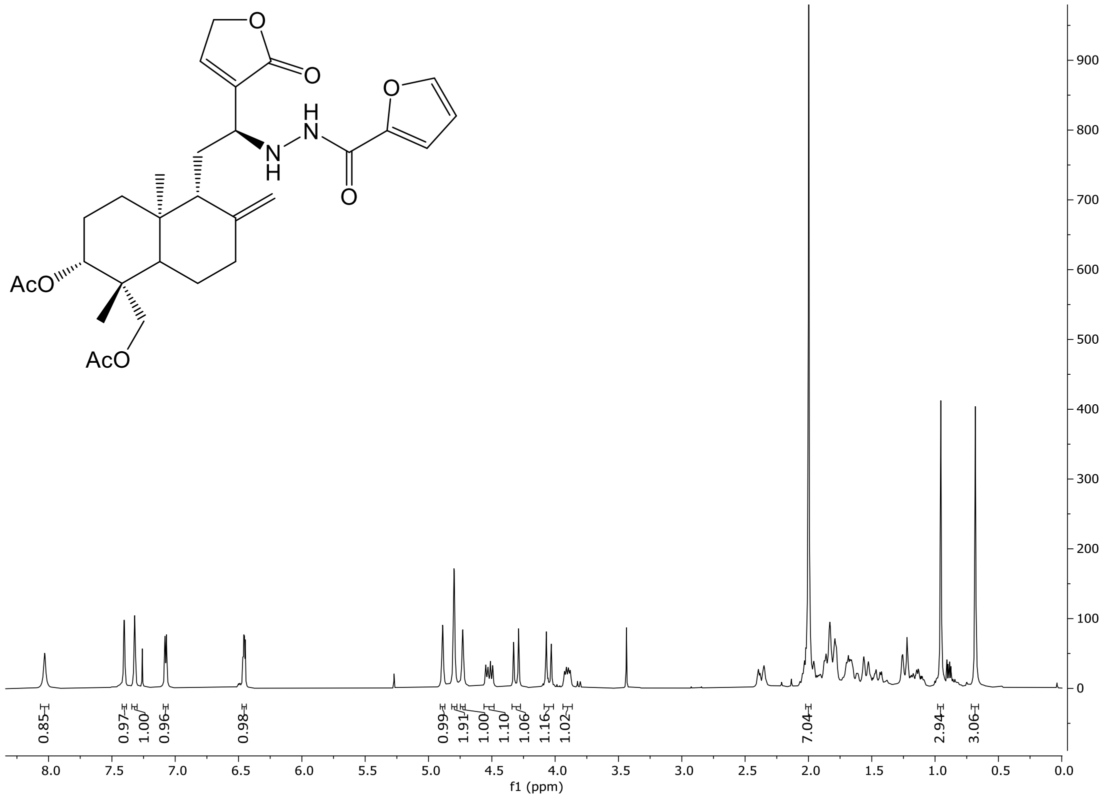

Figure S15: ^{1}H-NMR spectrum of compound **10** (300 MHz, $CDCl_3$)

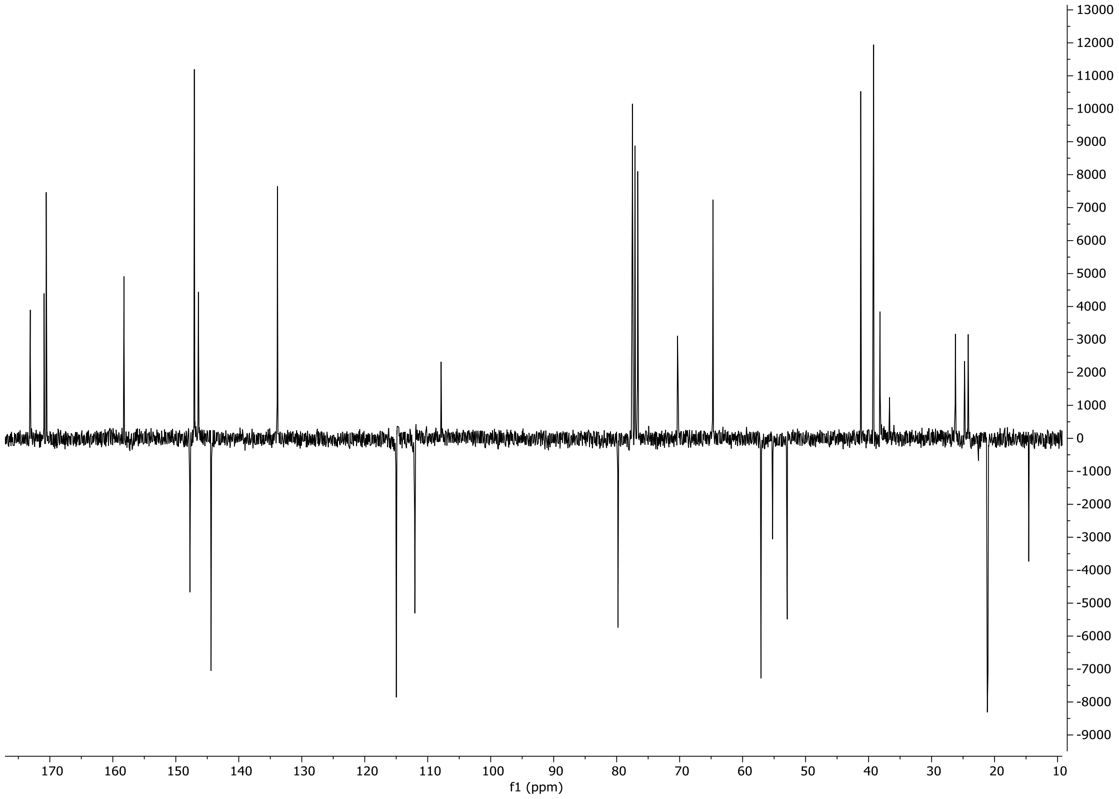

Figure S16: ^{13}C-APT NMR spectrum of compound **10** (75 MHz, $CDCl_3$)

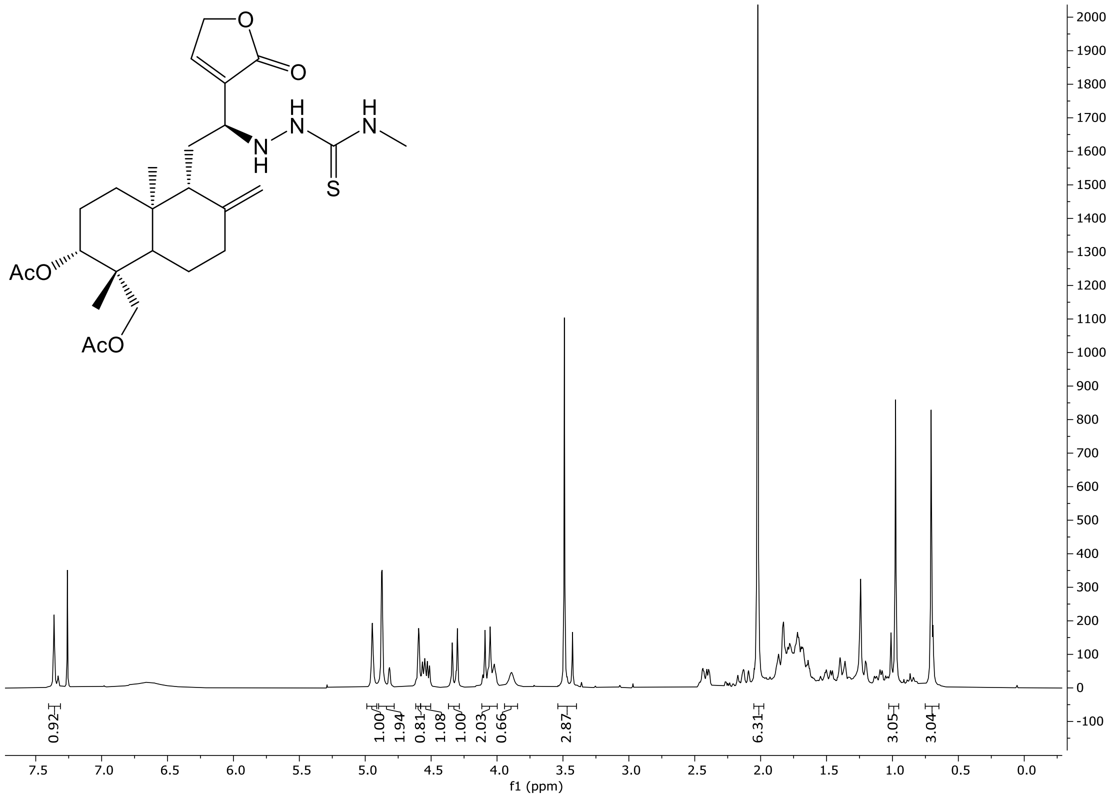

Figure S17: ^{1}H-NMR spectrum of compound **12** (300 MHz, $CDCl_3$)

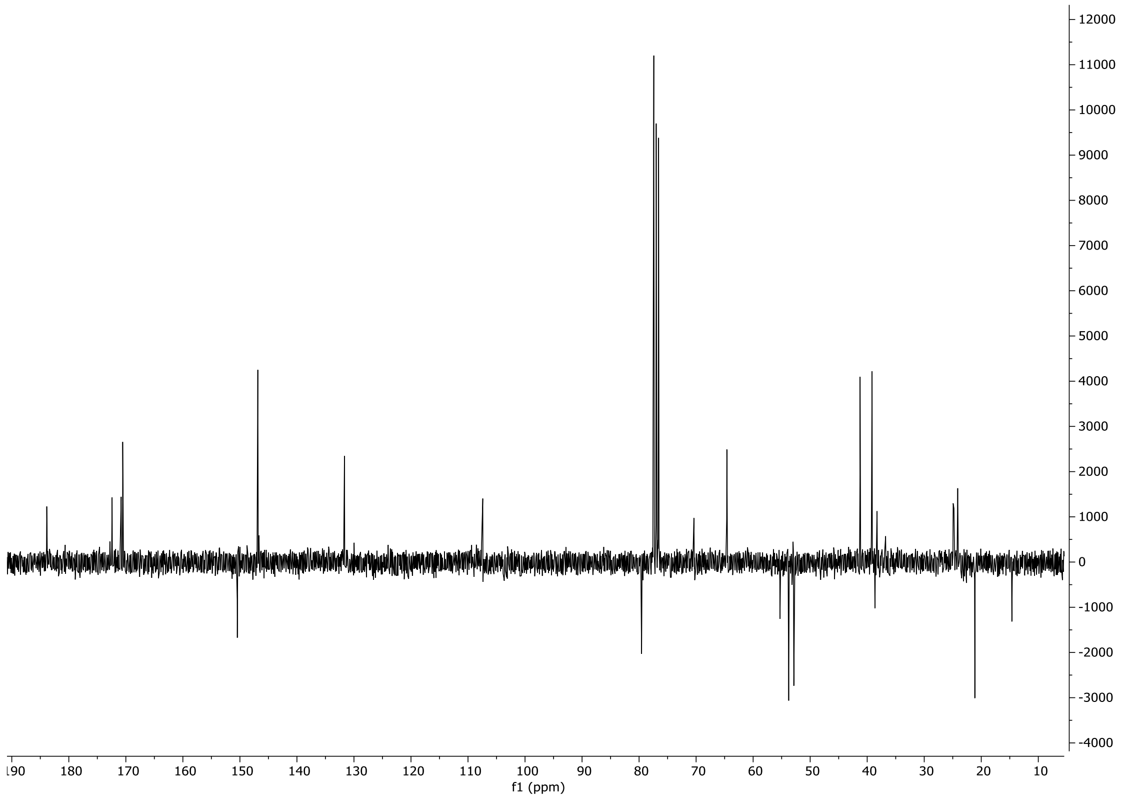

Figure S18: ^{13}C-APT NMR spectrum of compound **12** (75 MHz, $CDCl_3$)

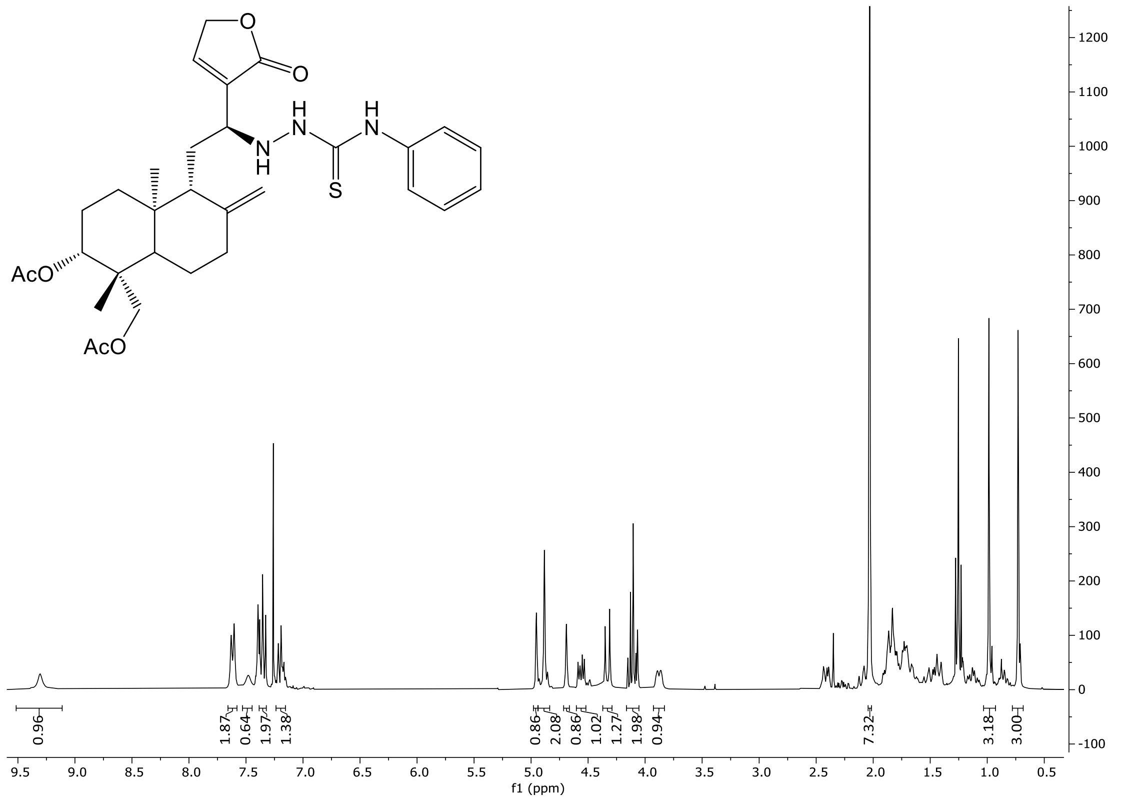

Figure S19: ^{1}H-NMR spectrum of compound **17** (300 MHz, $CDCl_3$)

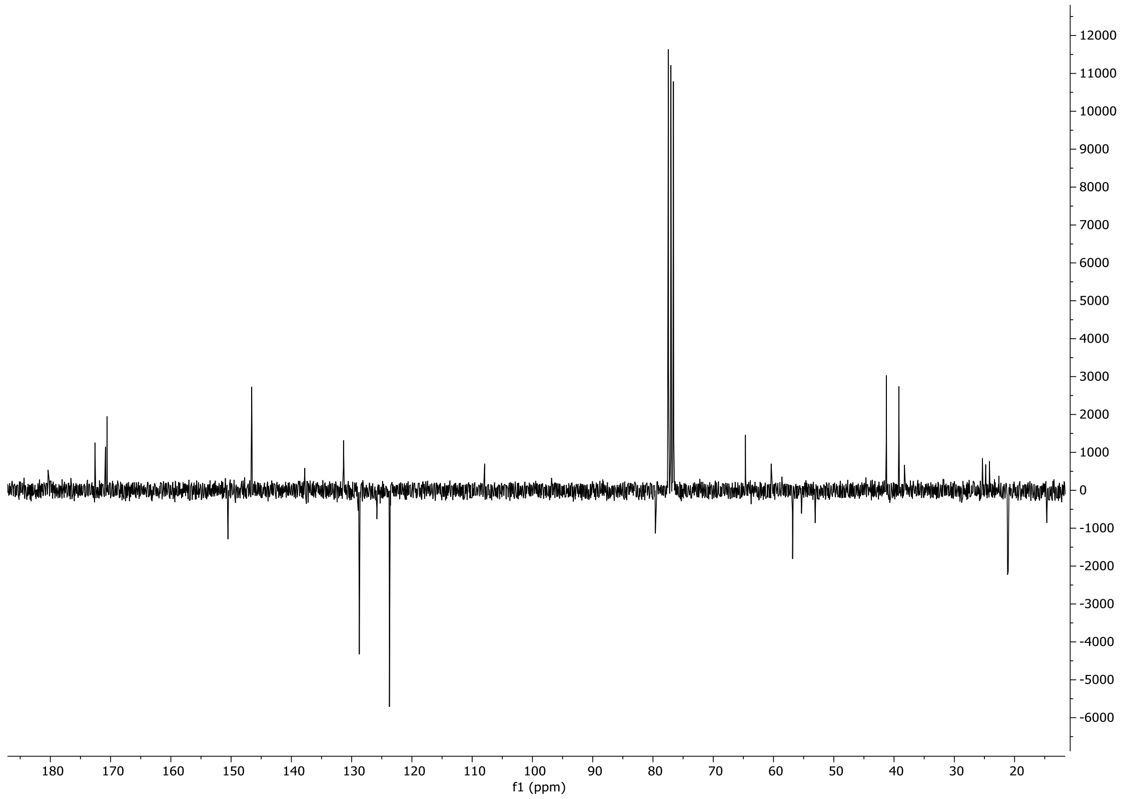

Figure S20: ^{13}C-APT NMR spectrum of compound **17** (75 MHz, $CDCl_3$)

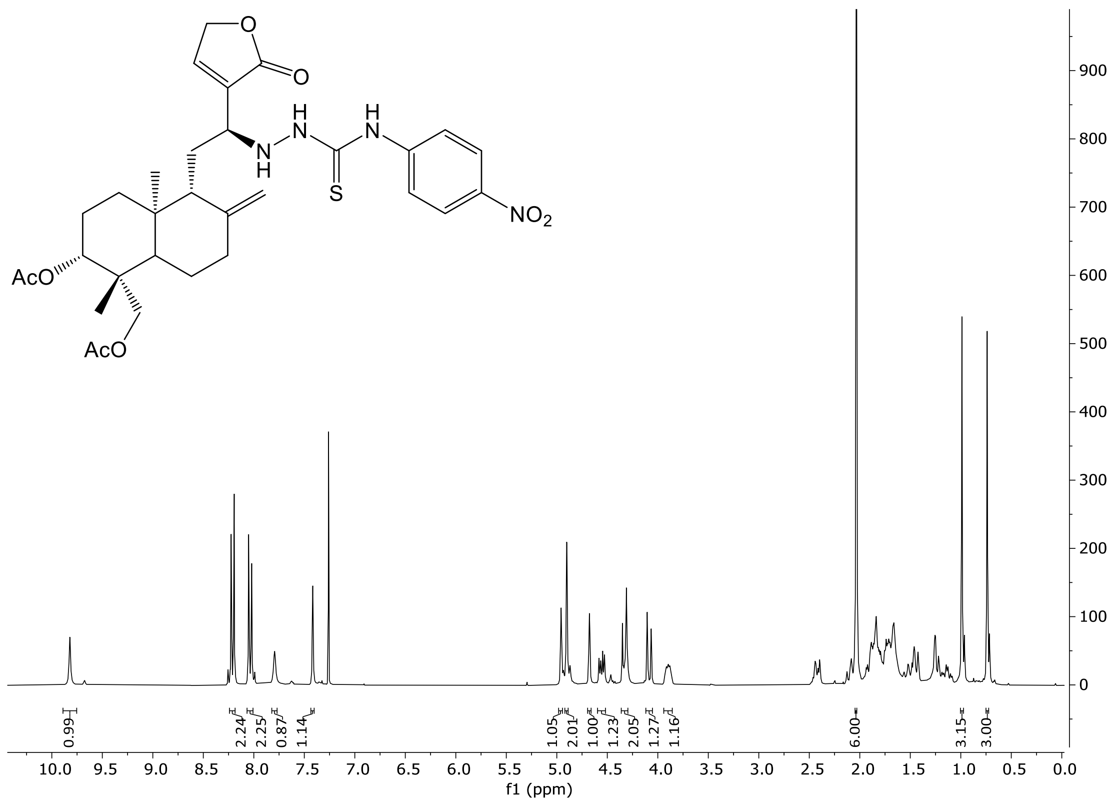

Figure S21: ^{1}H-NMR spectrum of compound **21** (300 MHz, $CDCl_3$)

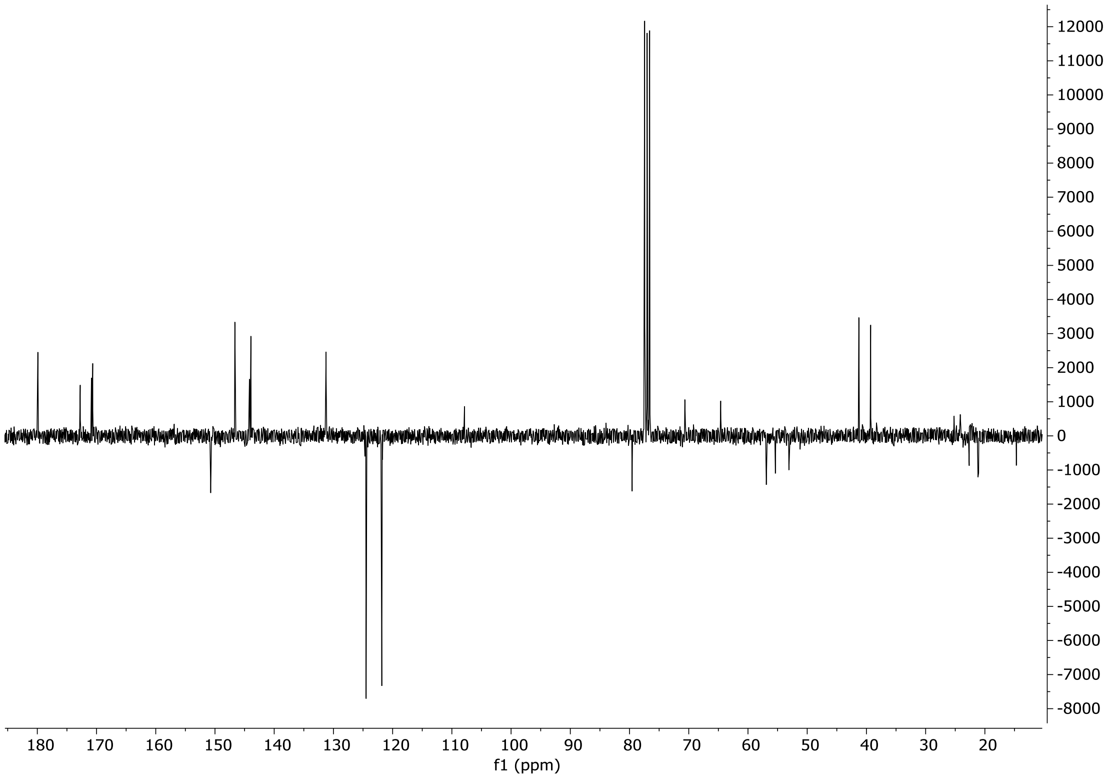

Figure S22: ^{13}C-APT NMR spectrum of compound **21** (75 MHz, $CDCl_3$)

3. Rhodamine-123 accumulation assay (compounds 1-25)

Table S1: P-pg inhibitory activity of compounds 1 – 25 on MDR-transfected mouse T-lymphoma cells.

Compound	R	Conc. (µM)	FAR[a]	FSC[b]	SSC[c]	FL-1[d]
PAR	-	-	-	2362	941	76.70
MDR	-	-	-	2444	1063	2.13
1	Andrographolide	2	0.96	2343	1326	1.32
		20	0.82	2242	1355	1.12
2	Triacetyl Andrographolide	2	1.95	1979	1395	2.68
		20	38.65	2367	1279	53.10
3	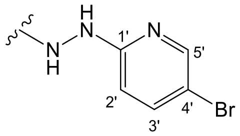	0.2	2.34	2461	1124	16.00
		2	16.12	2413	1162	110.00
4	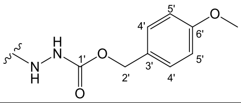	2	1.21	2307	1037	1.66
		20	64.99	2307	1037	68.10
5	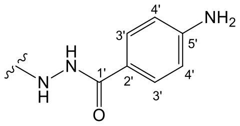	0.2	0.48	2501	1131	3.30
		2	0.48	2432	1116	3.28
6	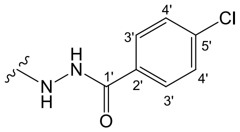	0.2	1.56	2469	1131	10.60
		2	3.36	2431	1144	22.90
7	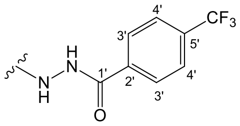	0.2	1.63	2469	1129	11.10
		2	5.19	2433	1148	35.40
8	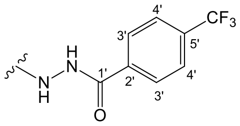	0.2	0.37	2463	1126	2.54
		2	2.27	2413	1210	15.50
9	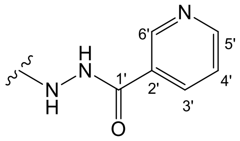	0.2	0.49	2472	1124	3.36
		2	0.32	2426	1191	2.21
10	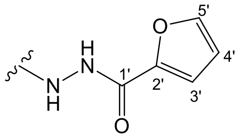	0.2	2.32	2503	1092	15.80
		2	4.28	2506	1248	29.20
11		0.2	0.54	2500	1106	3.67

	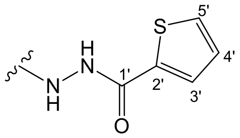	2	1.89	2448	1179	12.90

Table S1: Continuation

Compound	R	Conc. (µM)	FAR[a]	FSC[b]	SSC[c]	FL-1[d]
12	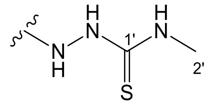	2	0.88	2299	1013	1.21
		20	41.85	2271	1012	57.50
13	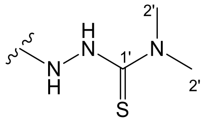	2	58.18	2142	1275	112.00
		20	61.30	2092	1296	118.00
14	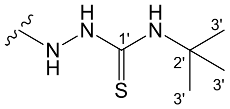	2	1.20	2289	1002	1.65
		20	43.52	2192	984	59.80
15	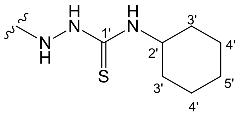	2	18.13	2225	1138	34.90
		20	64.94	2155	1217	125.0
16	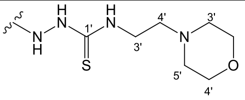	2	1.37	2216	1128	2.63
		20	50.23	2177	1163	96.70
17	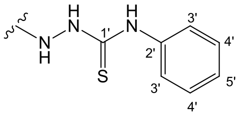	2	7.90	2226	1202	15.20
		20	55.07	2154	1144	106.00
18	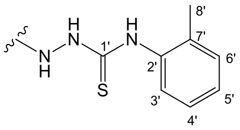	2	1.06	2302	1026	1.46
		20	37.12	2247	1037	51.50
19	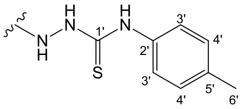	2	18.86	2154	1264	36.30
		20	60.26	2067	1357	116.00
20	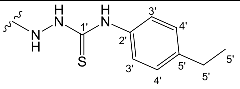	2	61.30	2160	1271	118.00
		20	79.48	2107	1300	153.00
21	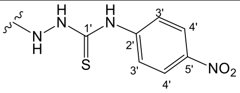	2	27.27	2226	1167	52.50
		20	51.95	2261	400	100.00
22	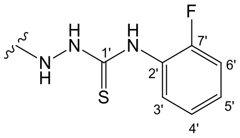	2	1.03	2319	1000	1.41
		20	79.33	2255	1060	109.00
23	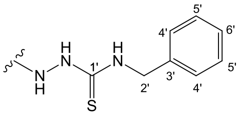	2	1.71	2260	1120	3.30
		20	57.14	2199	1200	110.0
24		2	14.29	2449	493	27.50

	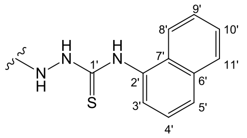	20	33.46	2090	576	64.40

Table S1: Continuation

Compound	R	Conc. (µM)	FAR[a]	FSC[b]	SSC[c]	FL-1[d]
25	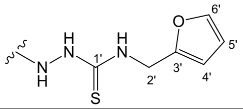	2	1.27	2164	1397	2.44
		20	31.12	2099	1300	59.90
Verapamil	-	20	5.14	2349	1222	9.90
DMSO	-	2%	0.96	2113	1230	1.85

[a]FAR (fluorescence activity ratio) values were determined by using the equation shown in section 4.5. Verapamil at 2 and 20 µM was used as positive control. DMSO 2% was used as negative control; [b]FSC: Forward scatter count of cells in the sample; [c]SSC: Side scatter count of cells in the sample; [d]FL-1: Mean fluorescence intensity of the cells.

4. Flow cytometry data

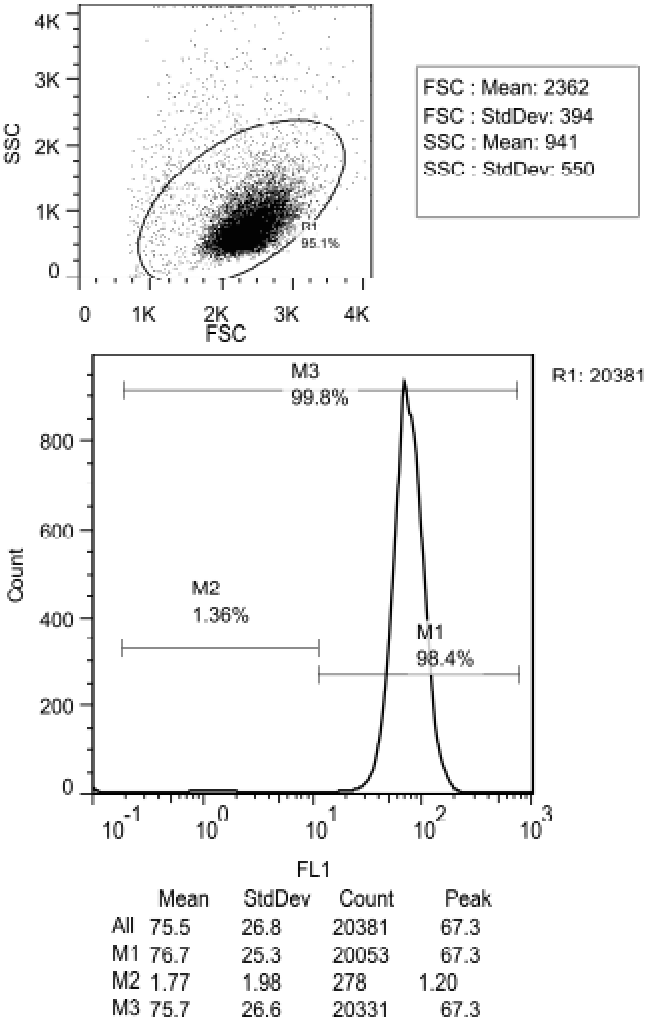

Figure S23: Flow cytometry data for sensitive L5178Y mouse T-lymphoma cells (PAR)

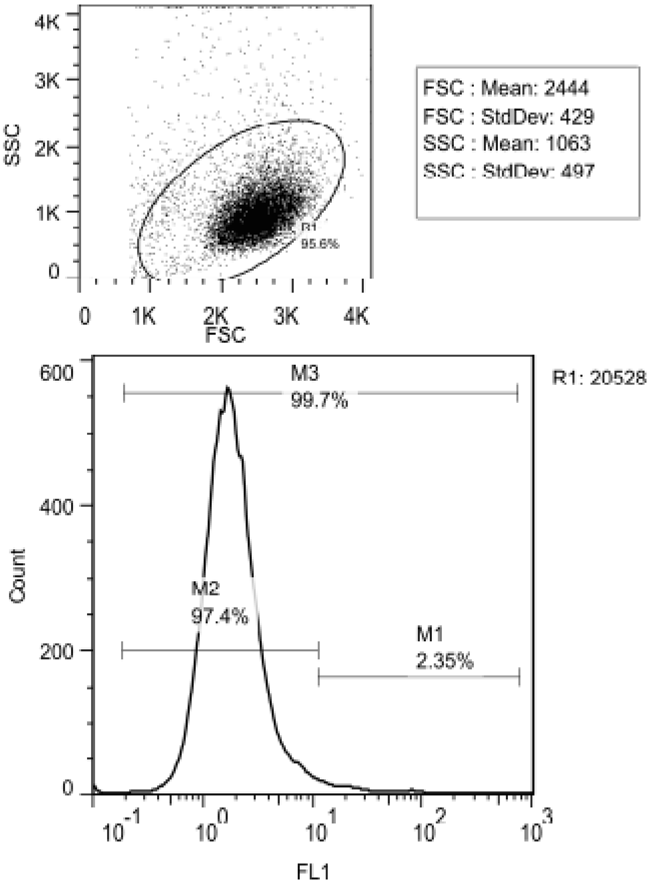

	Mean	StdDev	Count	Peak
All	2.85	6.51	20528	12.9
M1	33.4	27.6	482	12.9
M2	2.13	1.46	19989	1.60
M3	2.86	6.52	20471	1.60

Figure S24: Flow cytometry data for resistant human *ABCB1*-gene transfected L5178Y subline (MDR)

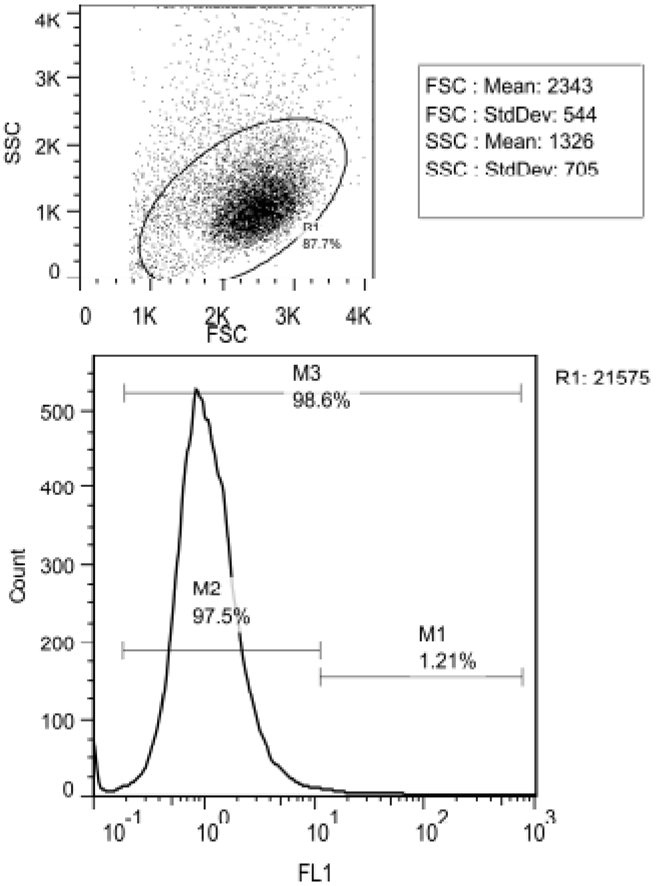

	Mean	StdDev	Count	Peak
All	1.76	5.75	21575	11.5
M1	38.5	35.8	260	11.5
M2	1.32	1.12	21033	0.965
M3	1.78	5.79	21280	0.965

Figure S25: Flow cytometry data for compound **1** tested at 2 µM in resistant human *ABCB1*-gene transfected L5178Y subline (MDR)

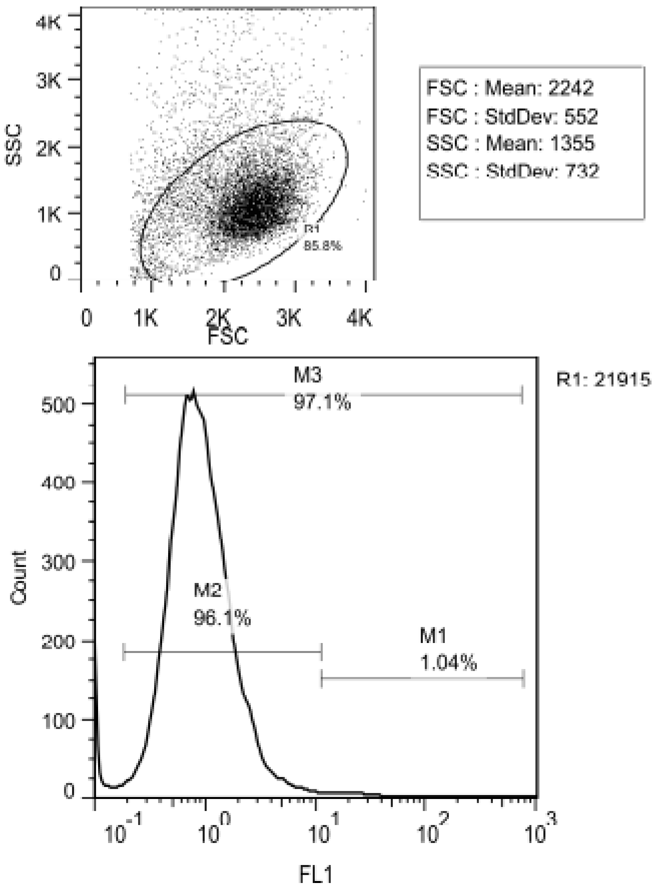

	Mean	StdDev	Count	Peak
All	1.47	5.13	21915	23.7
M1	37.4	33.5	229	23.7
M2	1.12	0.993	21061	0.777
M3	1.52	5.20	21272	0.777

Figure S26: Flow cytometry data for compound **1** tested at 20 µM in resistant human *ABCB1*-gene transfected L5178Y subline (MDR)

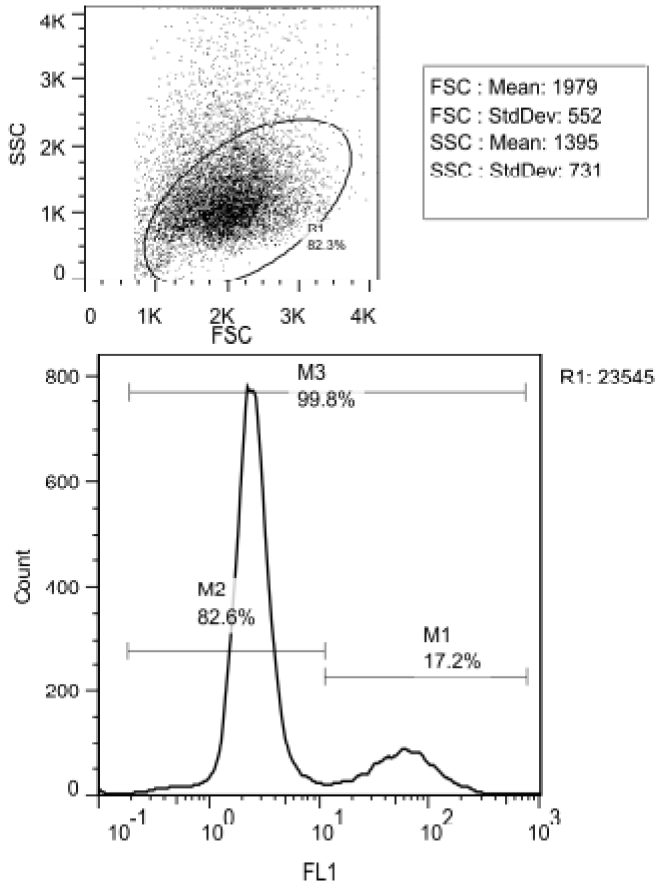

	Mean	StdDev	Count	Peak
All	14.3	34.0	23545	52.3
M1	70.2	54.1	4053	52.3
M2	2.68	1.30	19448	2.21
M3	14.3	34.0	23500	2.21

Figure S27: Flow cytometry data for compound **2** tested at 2 µM in resistant human *ABCB1*-gene transfected L5178Y subline (MDR)

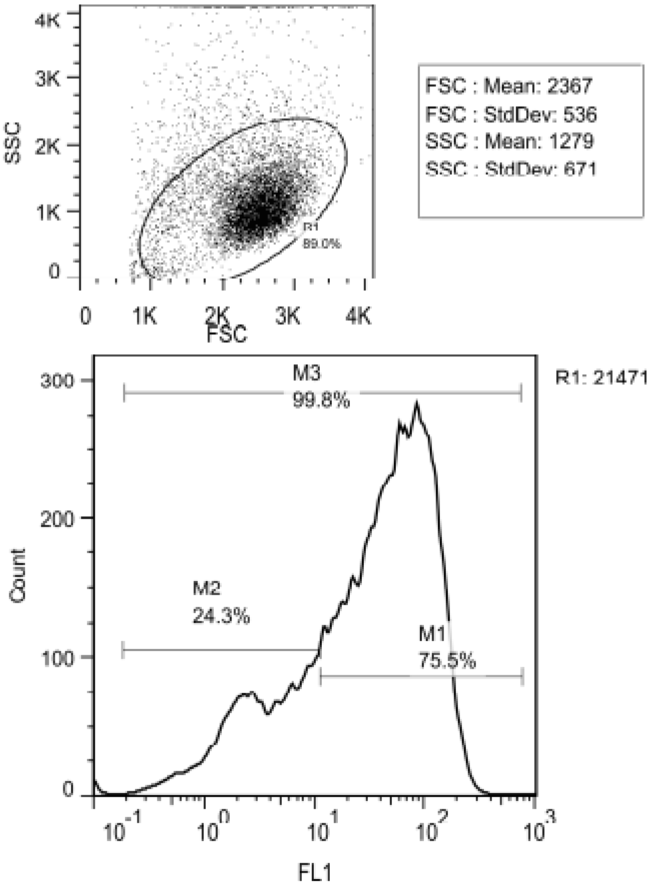

	Mean	StdDev	Count	Peak
All	53.0	50.6	21471	83.5
M1	68.8	48.6	16207	83.5
M2	4.38	3.06	5214	8.66
M3	53.1	50.5	21419	83.5

Figure S28: Flow cytometry data for compound **2** tested at 20 µM in resistant human *ABCB1*-gene transfected L5178Y subline (MDR)

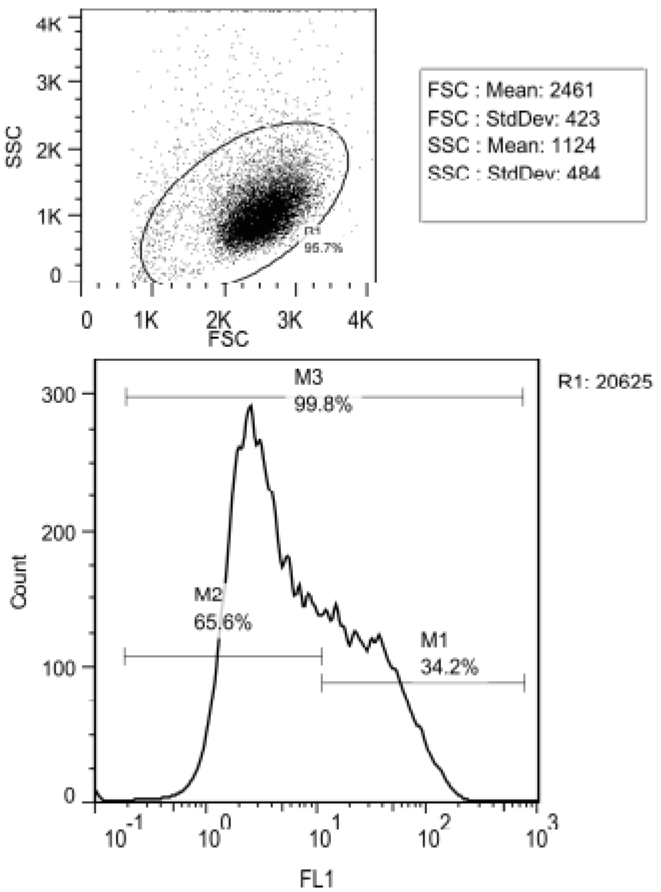

	Mean	StdDev	Count	Peak
All	16.0	25.2	20625	14.9
M1	39.3	31.9	7052	14.9
M2	3.89	2.54	13540	2.55
M3	16.0	25.2	20591	2.55

Figure S29: Flow cytometry data for compound **3** tested at 0.2 µM in resistant human *ABCB1*-gene transfected L5178Y subline (MDR)

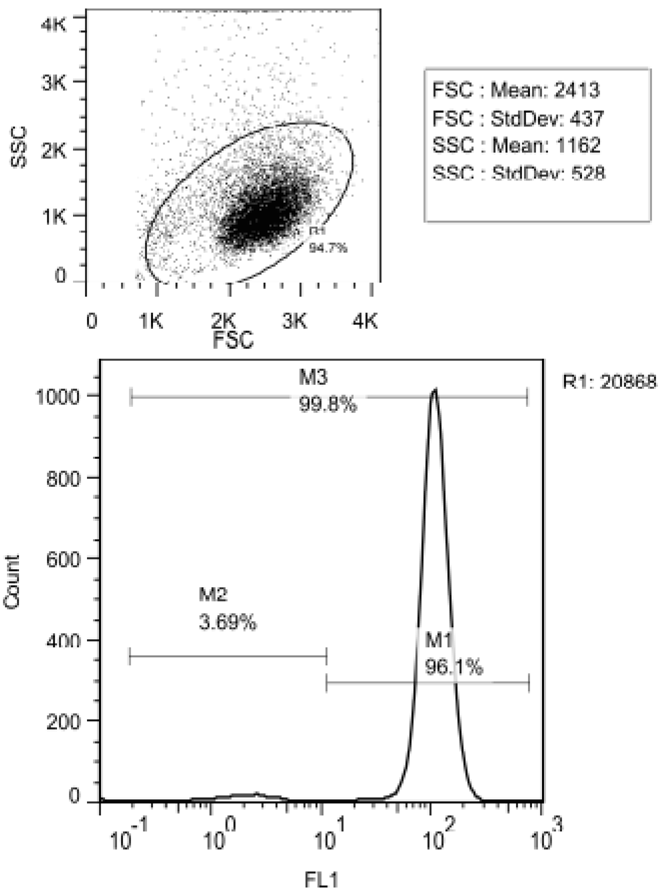

	Mean	StdDev	Count	Peak
All	106	38.1	20868	107
M1	110	32.6	20060	107
M2	2.30	1.70	769	2.74
M3	106	37.9	20829	107

Figure S30: Flow cytometry data for compound **3** tested at 2 µM in resistant human *ABCB1*-gene transfected L5178Y subline (MDR)

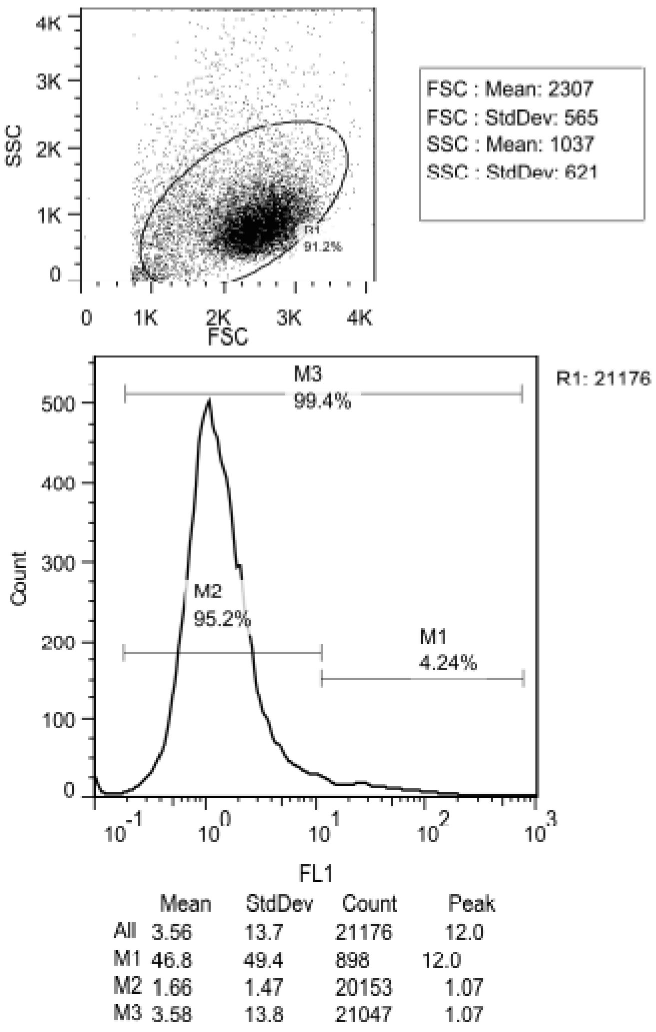

Figure S31: Flow cytometry data for compound **4** tested at 2 µM in resistant human *ABCB1*-gene transfected L5178Y subline (MDR)

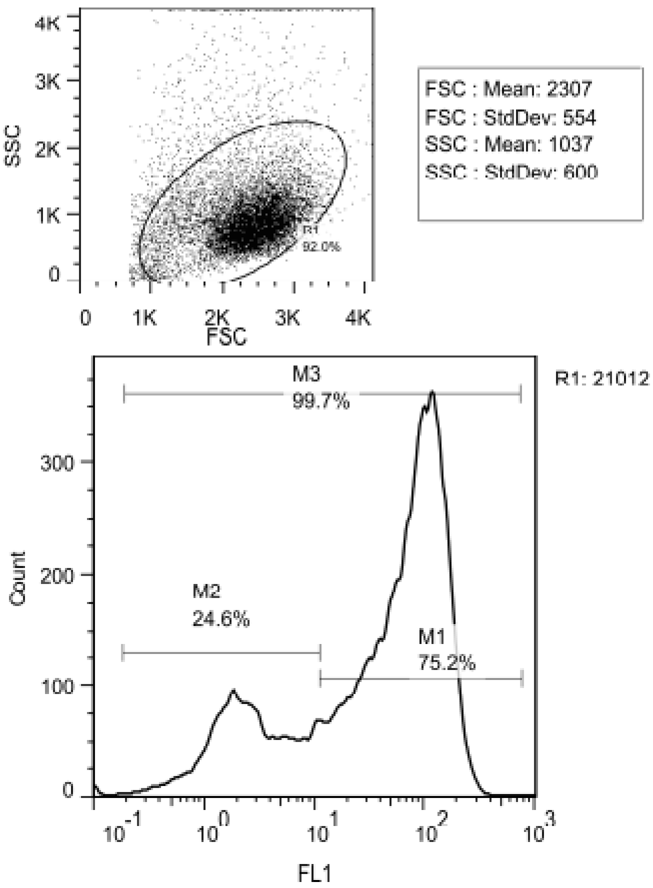

	Mean	StdDev	Count	Peak
All	68.0	60.5	21012	120
M1	89.3	55.1	15793	120
M2	3.50	2.81	5168	1.84
M3	68.1	60.4	20958	120

Figure S32: Flow cytometry data for compound **4** tested at 20 µM in resistant human *ABCB1*-gene transfected L5178Y subline (MDR)

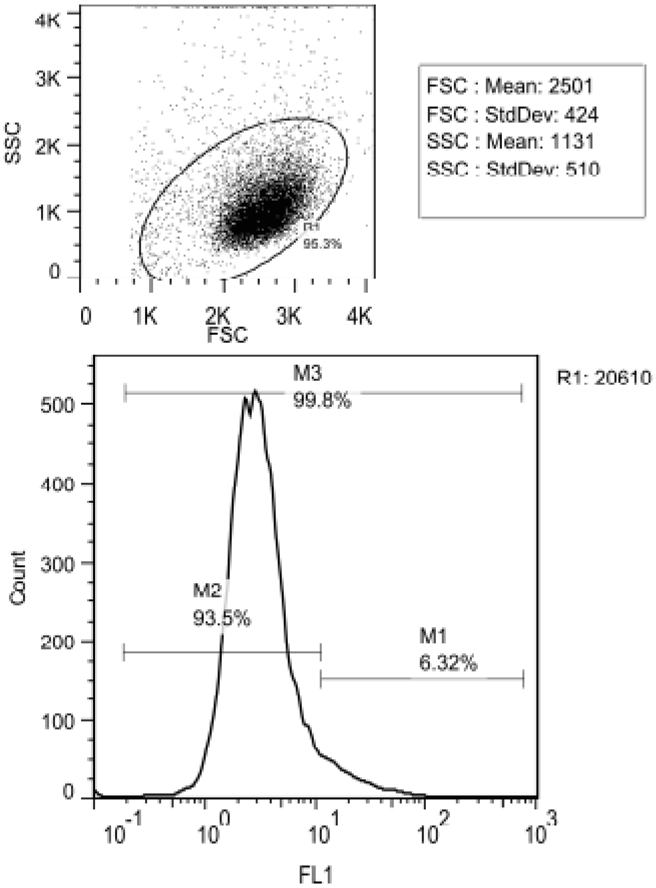

	Mean	StdDev	Count	Peak
All	4.84	9.19	20610	12.0
M1	27.8	26.9	1302	12.0
M2	3.30	1.87	19269	2.29
M3	4.85	9.20	20570	2.29

Figure S33: Flow cytometry data for compound **5** tested at 0.2 µM in resistant human *ABCB1*-gene transfected L5178Y subline (MDR)

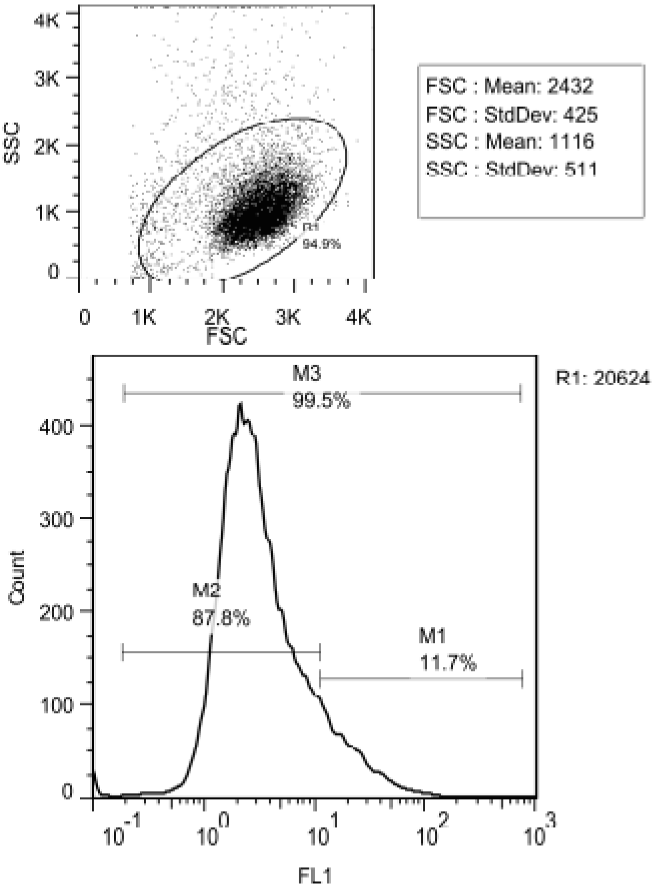

	Mean	StdDev	Count	Peak
All	5.95	10.5	20624	12.9
M1	26.3	21.0	2404	12.9
M2	3.28	2.25	18110	2.05
M3	5.98	10.5	20514	2.05

Figure S34: Flow cytometry data for compound **5** tested at 2 µM in resistant human *ABCB1*-gene transfected L5178Y subline (MDR)

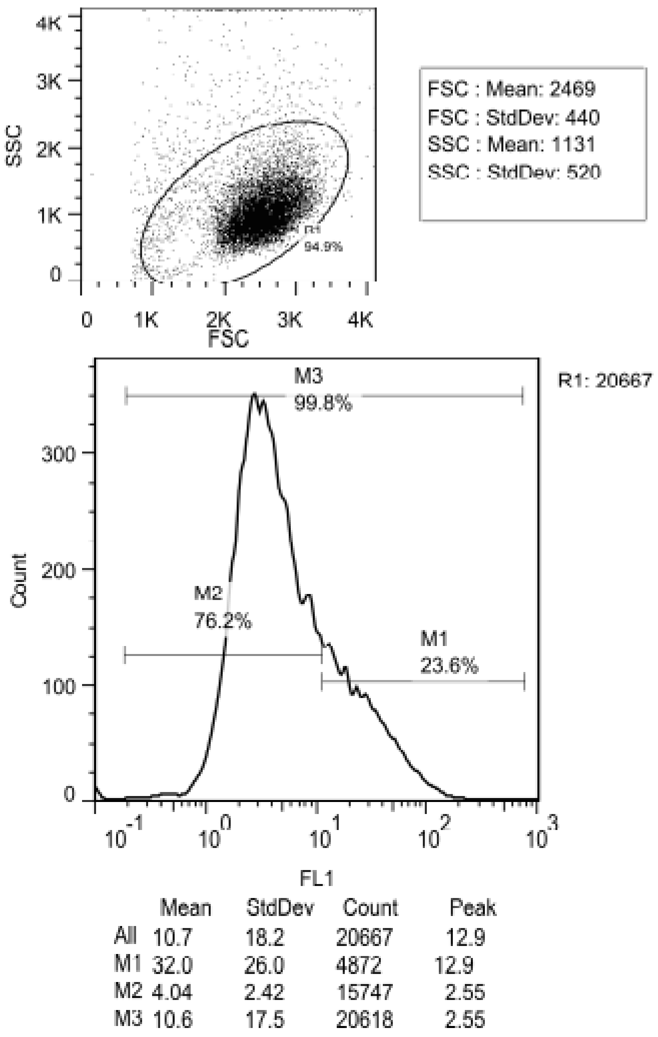

Figure S35: Flow cytometry data for compound **6** tested at 0.2 µM in resistant human *ABCB1*-gene transfected L5178Y subline (MDR)

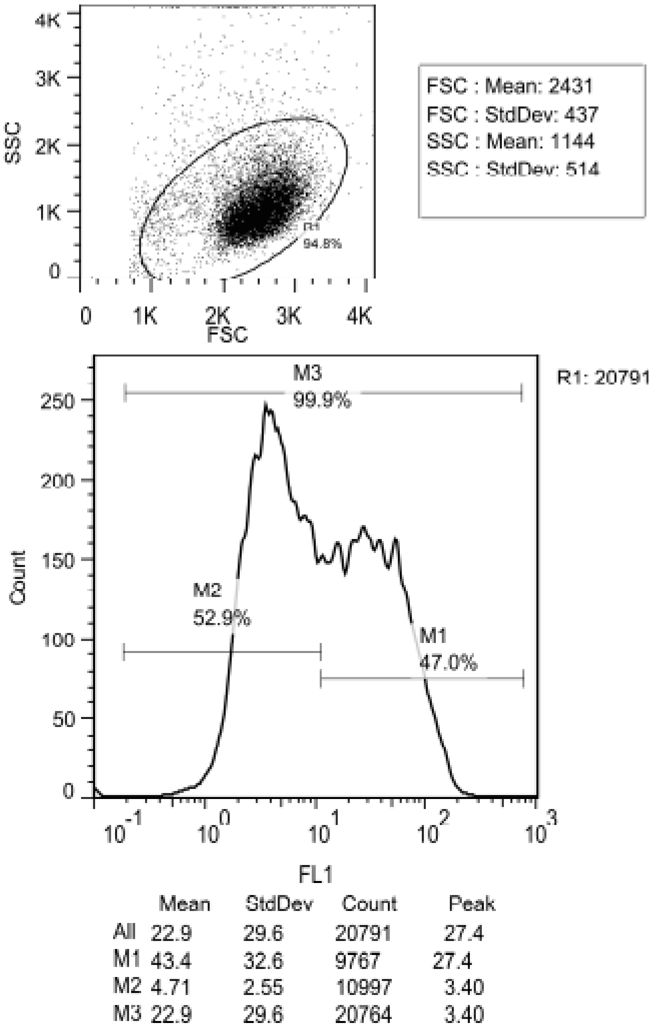

Figure S36: Flow cytometry data for compound **6** tested at 2 µM in resistant human *ABCB1*-gene transfected L5178Y subline (MDR)

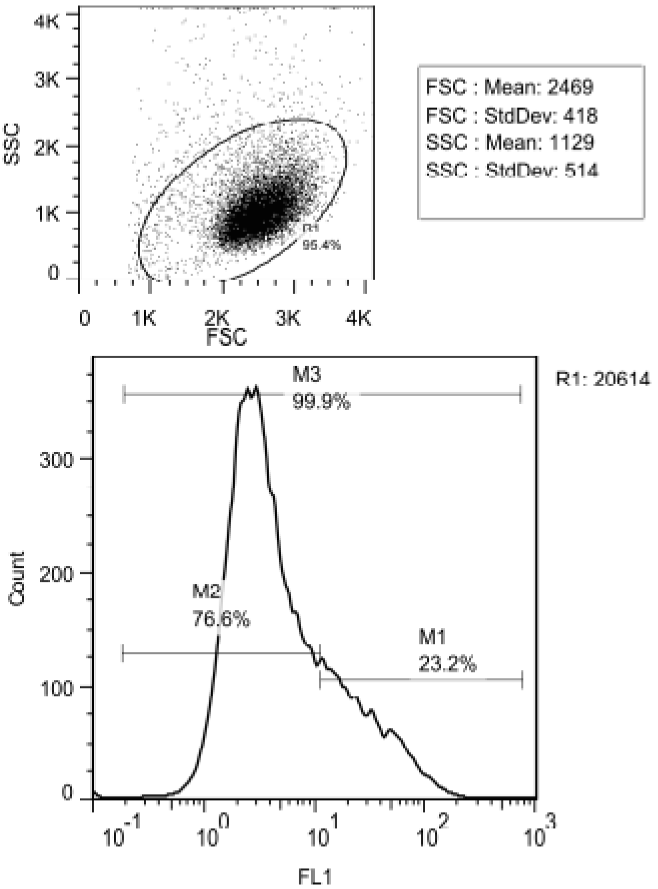

	Mean	StdDev	Count	Peak
All	11.1	20.0	20614	11.5
M1	35.7	30.4	4790	11.5
M2	3.68	2.33	15797	2.46
M3	11.1	20.1	20586	2.46

Figure S37: Flow cytometry data for compound **7** tested at 0.2 µM in resistant human *ABCB1*-gene transfected L5178Y subline (MDR)

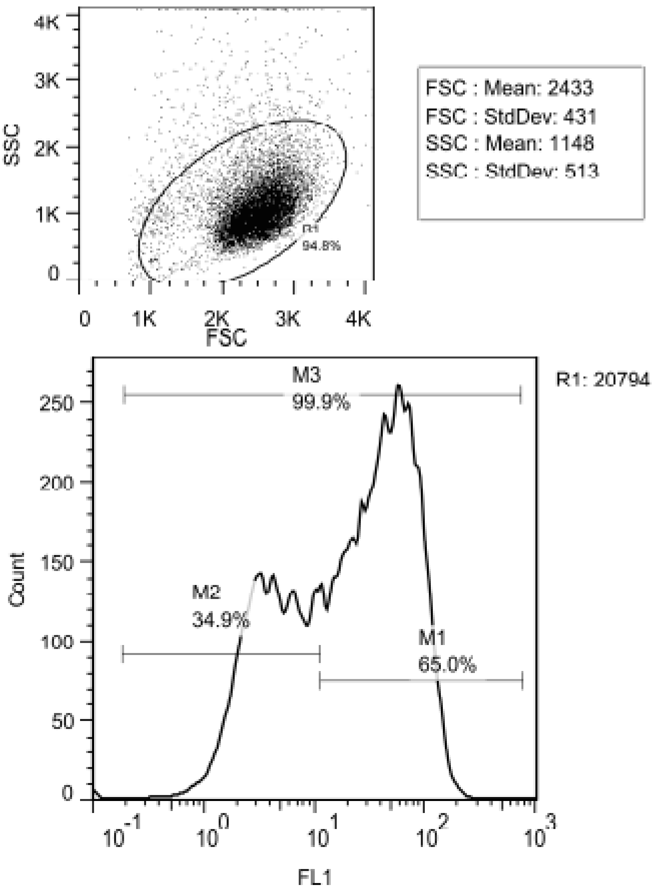

	Mean	StdDev	Count	Peak
All	35.3	35.6	20794	54.2
M1	51.9	34.1	13506	54.2
M2	4.75	2.73	7263	3.28
M3	35.4	35.6	20769	54.2

Figure S38: Flow cytometry data for compound **7** tested at 2 µM in resistant human *ABCB1*-gene transfected L5178Y subline (MDR)

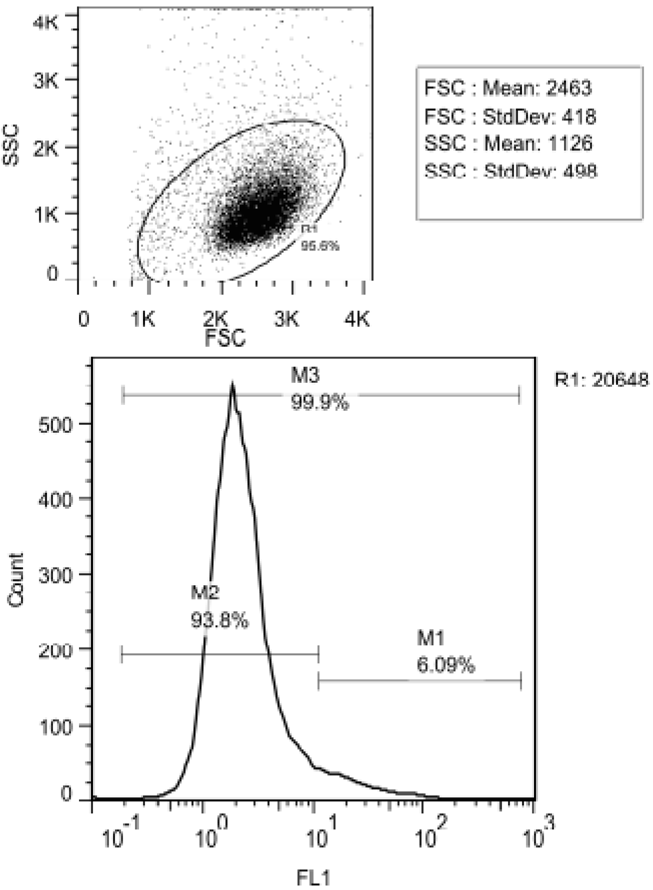

	Mean	StdDev	Count	Peak
All	4.34	10.3	20648	12.0
M1	32.1	29.9	1257	12.0
M2	2.54	1.72	19369	1.84
M3	4.34	10.4	20625	1.84

Figure S39: Flow cytometry data for compound **8** tested at 0.2 µM in resistant human *ABCB1*-gene transfected L5178Y subline (MDR)

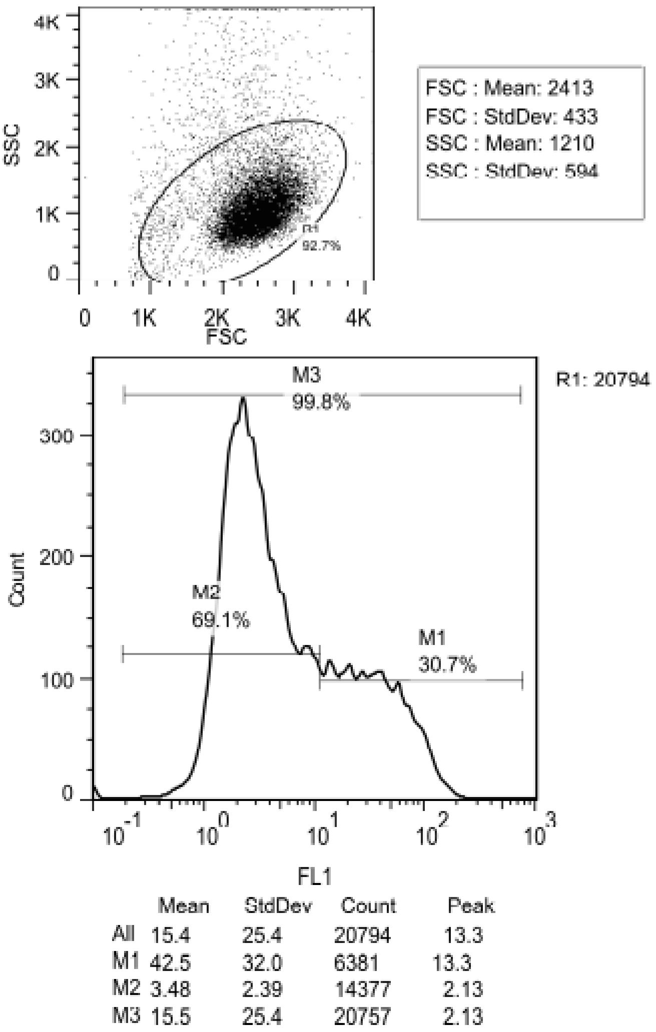

Figure S40: Flow cytometry data for compound **8** tested at 2 µM in resistant human *ABCB1*-gene transfected L5178Y subline (MDR)

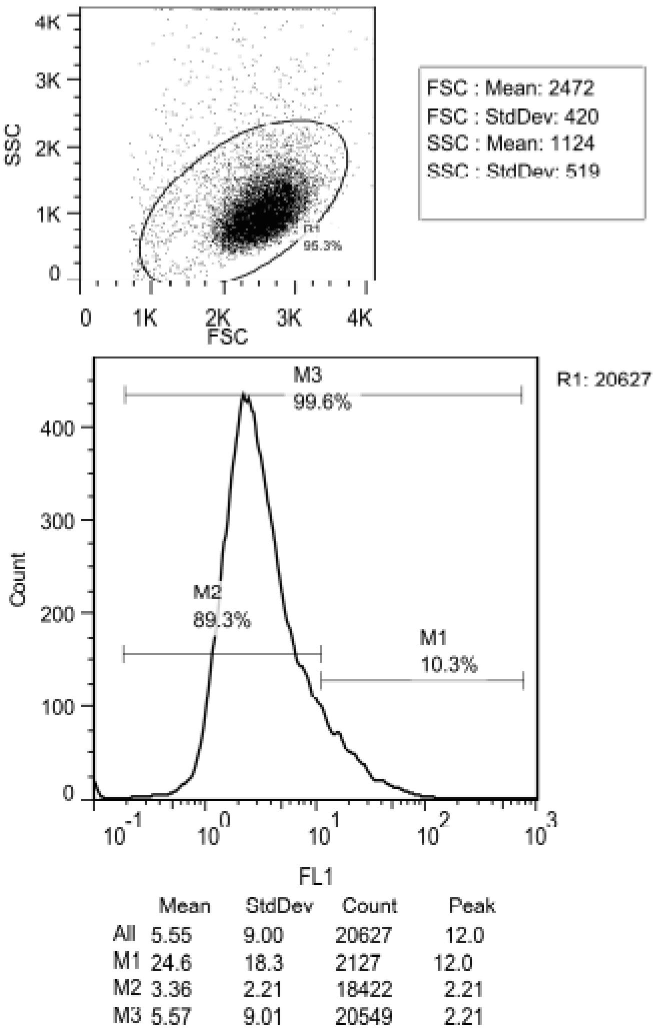

Figure S41: Flow cytometry data for compound **9** tested at 0.2 µM in resistant human *ABCB1*-gene transfected L5178Y subline (MDR)

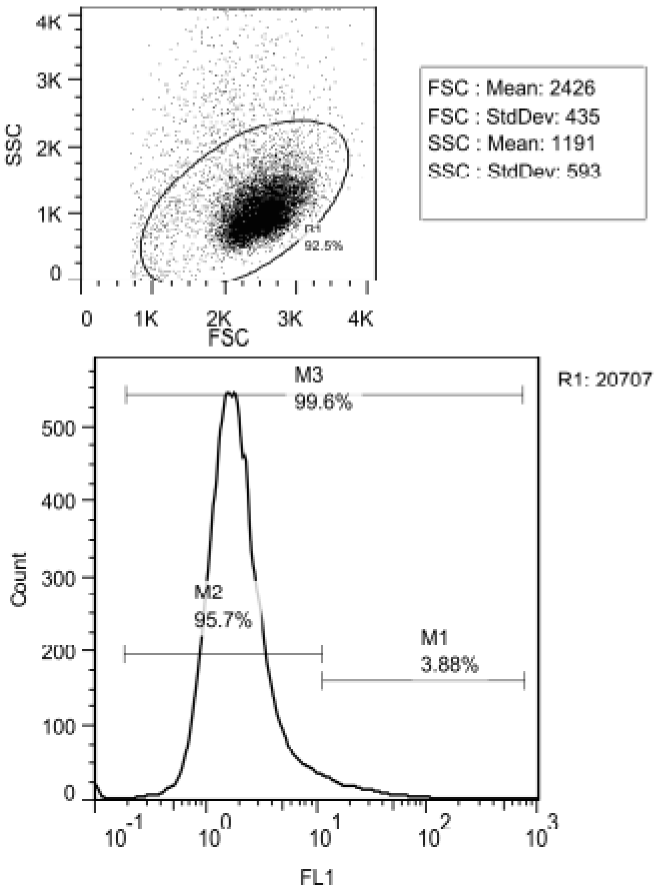

	Mean	StdDev	Count	Peak
All	3.26	7.48	20707	12.9
M1	29.5	25.9	803	12.9
M2	2.21	1.58	19824	1.49
M3	3.27	7.50	20625	1.49

Figure S42: Flow cytometry data for compound **9** tested at 2 µM in resistant human *ABCB1*-gene transfected L5178Y subline (MDR)

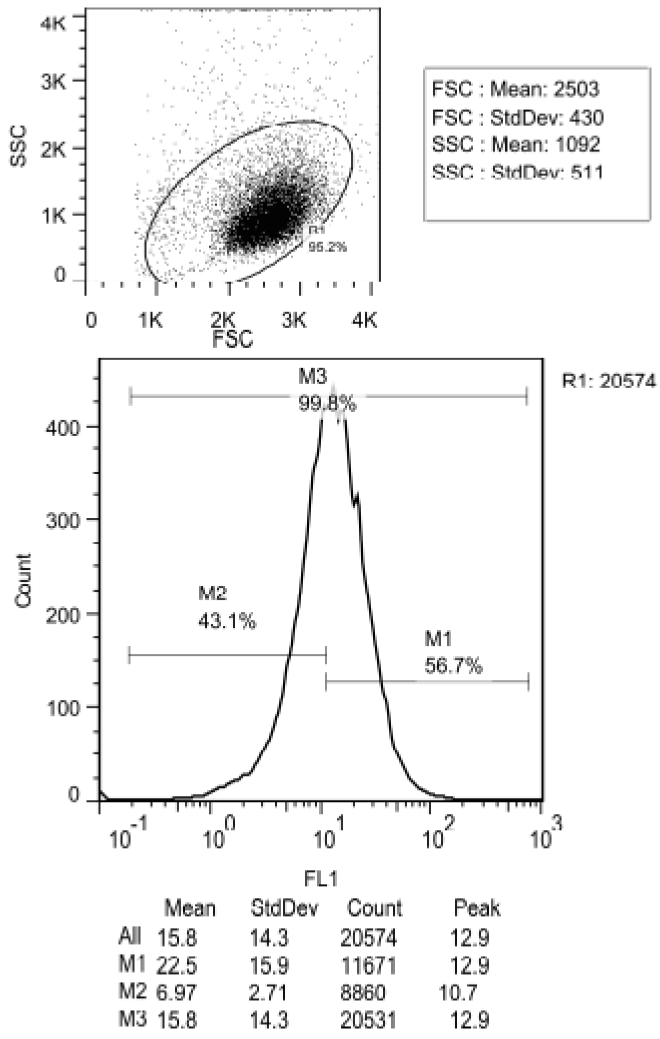

Figure S43: Flow cytometry data for compound **10** tested at 0.2 µM in resistant human *ABCB1*-gene transfected L5178Y subline (MDR)

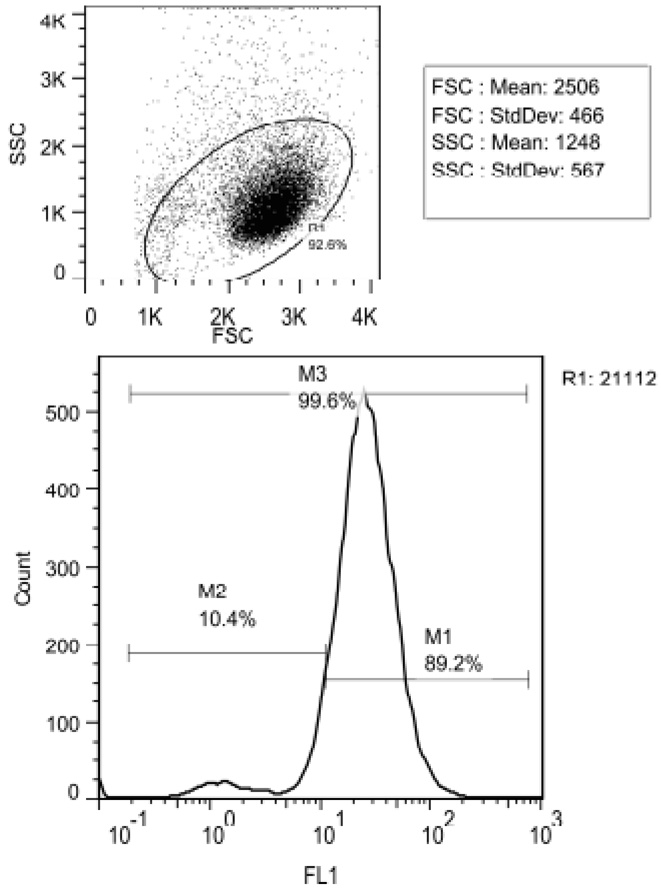

	Mean	StdDev	Count	Peak
All	29.0	20.1	21112	24.6
M1	31.8	19.5	18837	24.6
M2	6.16	3.88	2194	10.7
M3	29.2	20.1	21030	24.6

Figure S44: Flow cytometry data for compound **10** tested at 2 µM in resistant human *ABCB1*-gene transfected L5178Y subline (MDR)

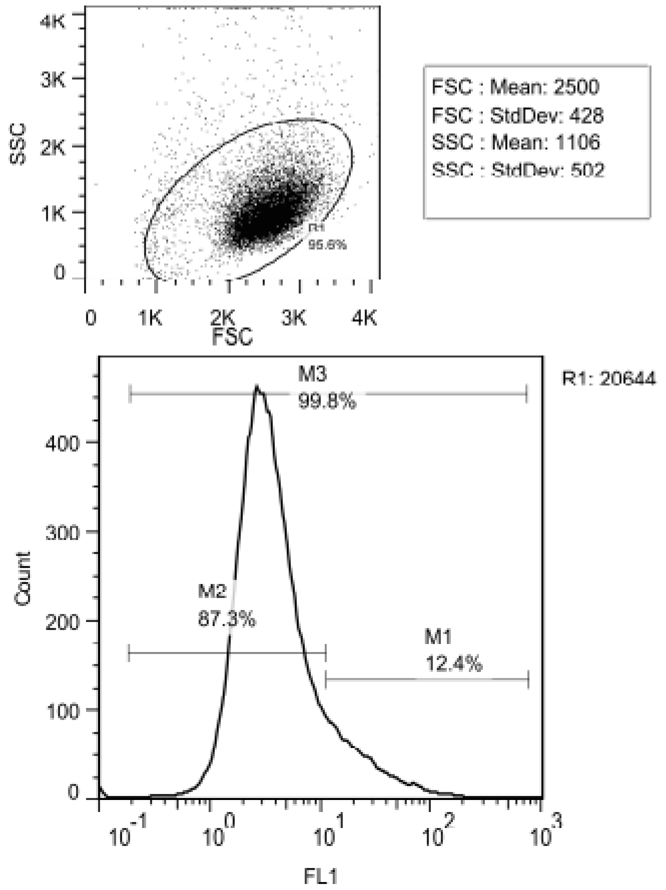

	Mean	StdDev	Count	Peak
All	7.13	14.3	20644	11.5
M1	31.5	30.3	2569	11.5
M2	3.67	2.13	18027	2.64
M3	7.15	14.3	20595	2.64

Figure S45: Flow cytometry data for compound **11** tested at 0.2 µM in resistant human *ABCB1*-gene transfected L5178Y subline (MDR)

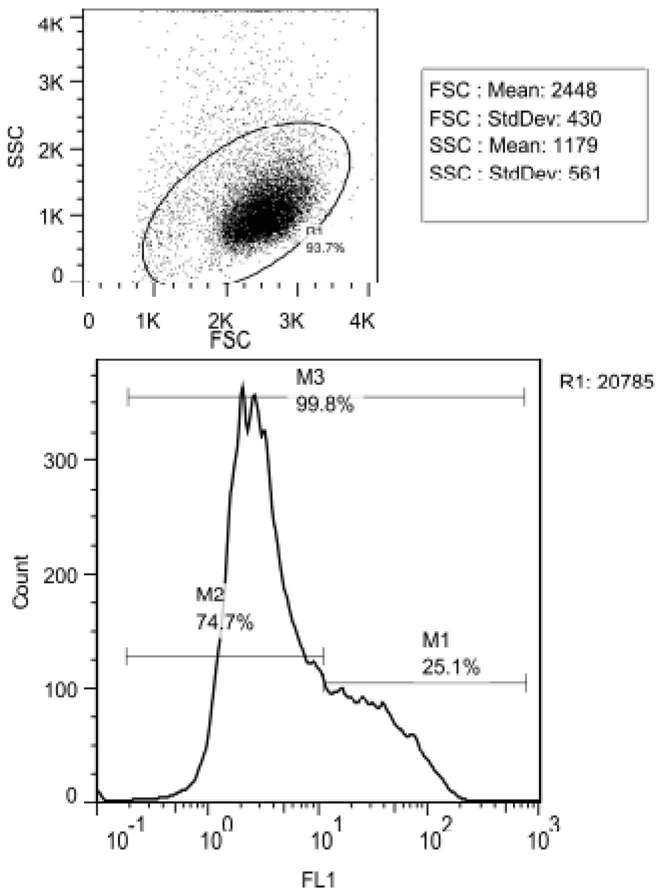

	Mean	StdDev	Count	Peak
All	12.9	23.3	20785	16.0
M1	40.7	33.2	5224	16.0
M2	3.52	2.32	15521	2.05
M3	12.9	23.3	20744	2.05

Figure S46: Flow cytometry data for compound **11** tested at 2 µM in resistant human *ABCB1*-gene transfected L5178Y subline (MDR)

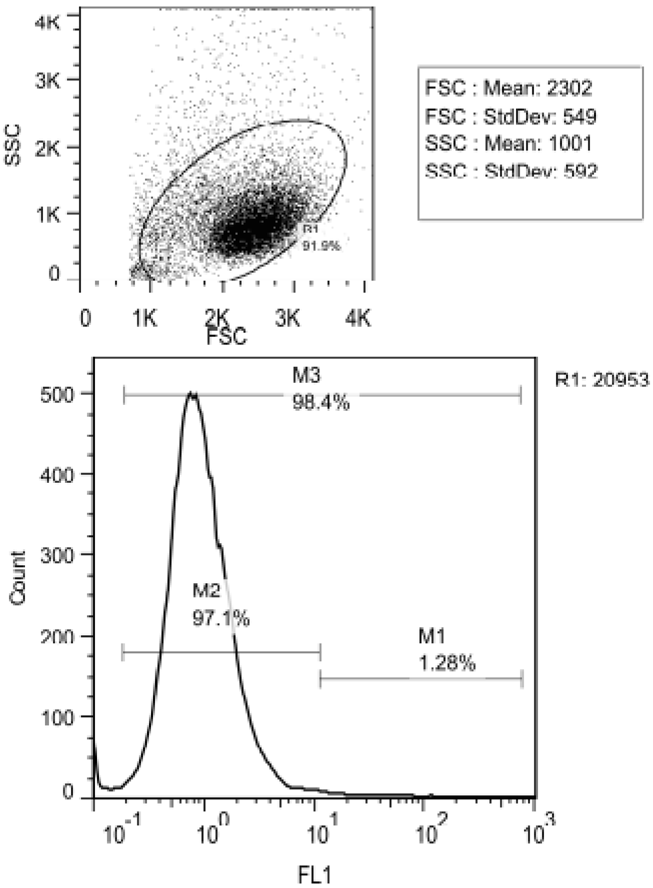

	Mean	StdDev	Count	Peak
All	1.78	8.58	20953	11.5
M1	51.4	56.3	269	11.5
M2	1.16	1.03	20348	0.835
M3	1.81	8.65	20608	0.835

Figure S47: Flow cytometry data for compound **12** tested at 2 µM in resistant human *ABCB1*-gene transfected L5178Y subline (MDR)

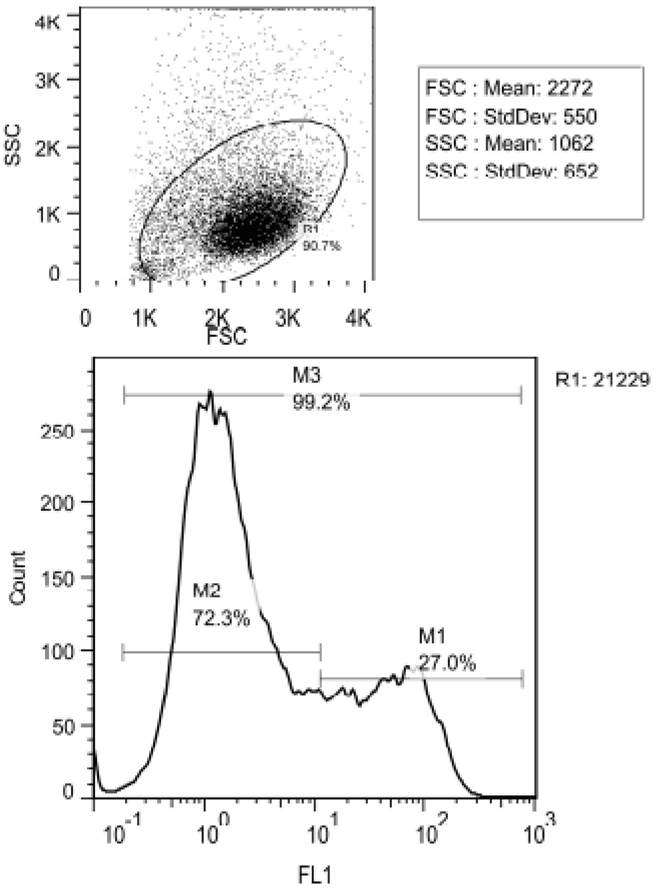

	Mean	StdDev	Count	Peak
All	17.7	35.0	21229	64.9
M1	59.4	46.3	5723	64.9
M2	2.28	2.24	15346	1.38
M3	17.8	35.1	21062	1.38

Figure S48: Flow cytometry data for compound **12** tested at 20 µM in resistant human *ABCB1*-gene transfected L5178Y subline (MDR)

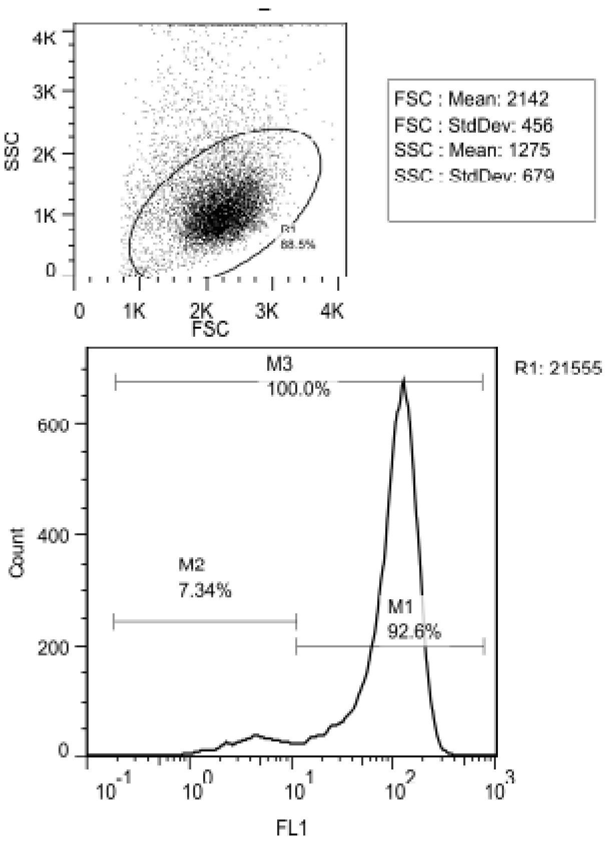

	Mean	StdDev	Count	Peak
All	104	56.8	21555	124
M1	112	51.4	19962	124
M2	4.71	2.67	1583	4.22
M3	104	56.8	21545	124

Figure S49: Flow cytometry data for compound **13** tested at 2 µM in resistant human *ABCB1*-gene transfected L5178Y subline (MDR)

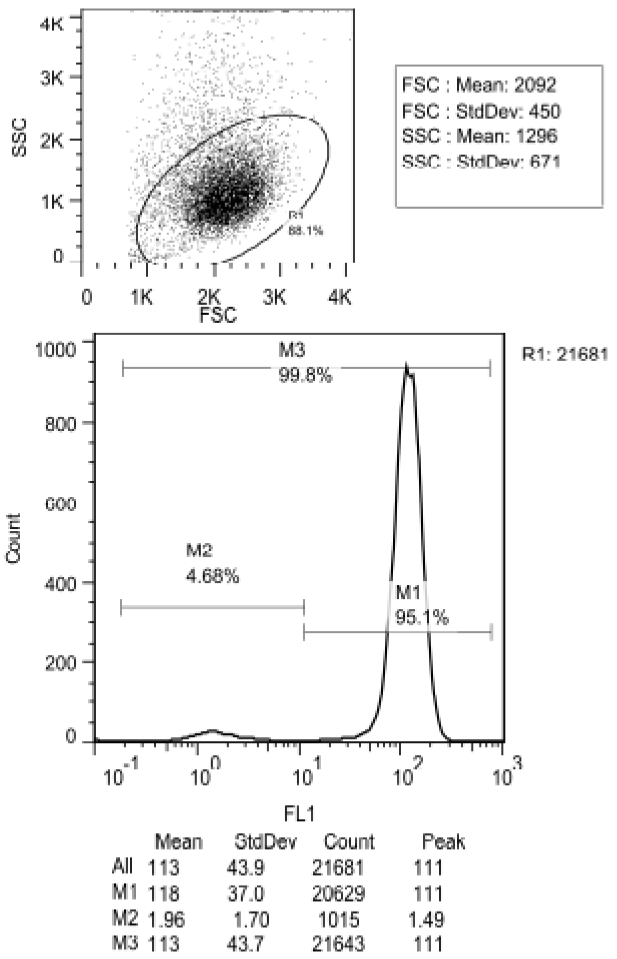

Figure S50: Flow cytometry data for compound **13** tested at 20 µM in resistant human *ABCB1*-gene transfected L5178Y subline (MDR)

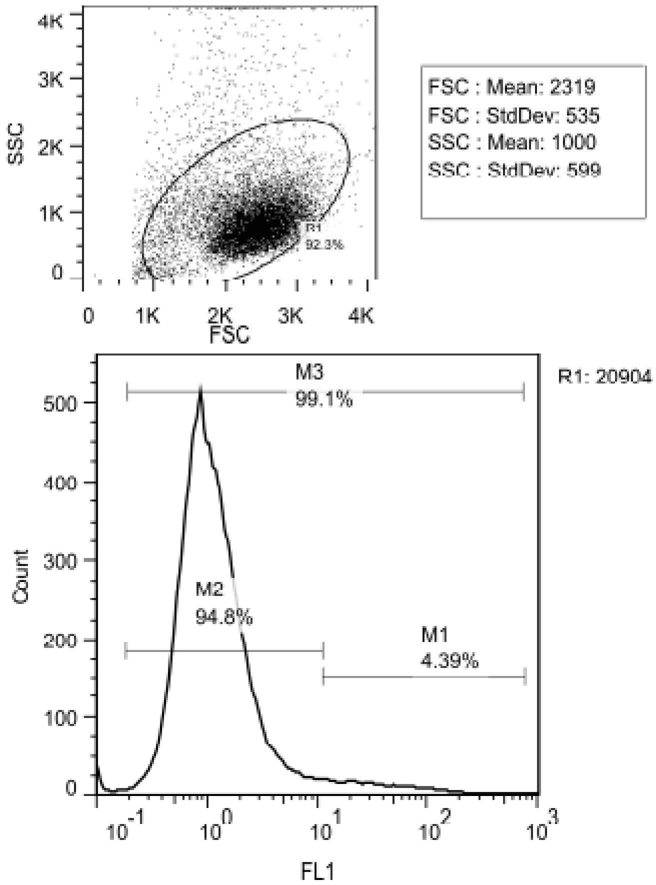

	Mean	StdDev	Count	Peak
All	3.48	13.8	20904	13.8
M1	48.9	46.3	917	13.8
M2	1.41	1.31	19815	0.866
M3	3.51	13.9	20724	0.866

Figure S51: Flow cytometry data for compound **14** tested at 2 µM in resistant human *ABCB1*-gene transfected L5178Y subline (MDR)

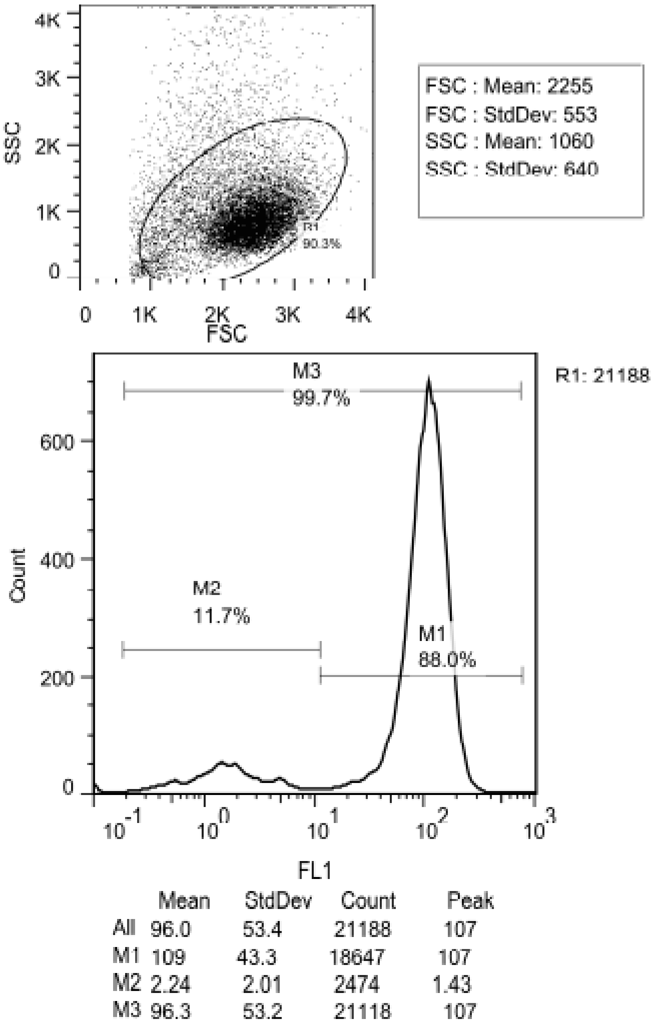

Figure S52: Flow cytometry data for compound **14** tested at 20 µM in resistant human *ABCB1*-gene transfected L5178Y subline (MDR)

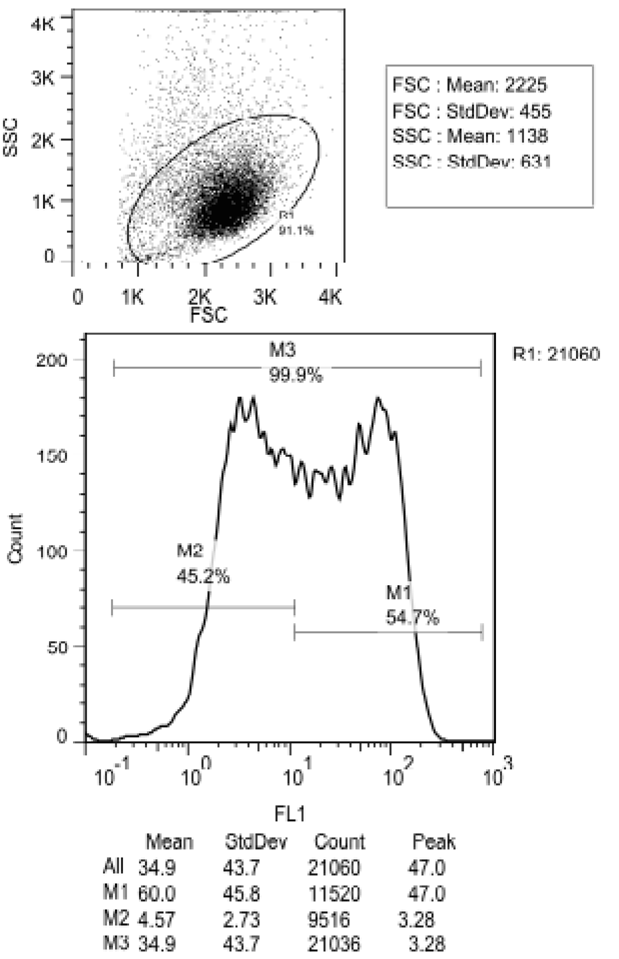

Figure S53: Flow cytometry data for compound **15** tested at 2 µM in resistant human *ABCB1*-gene transfected L5178Y subline (MDR)

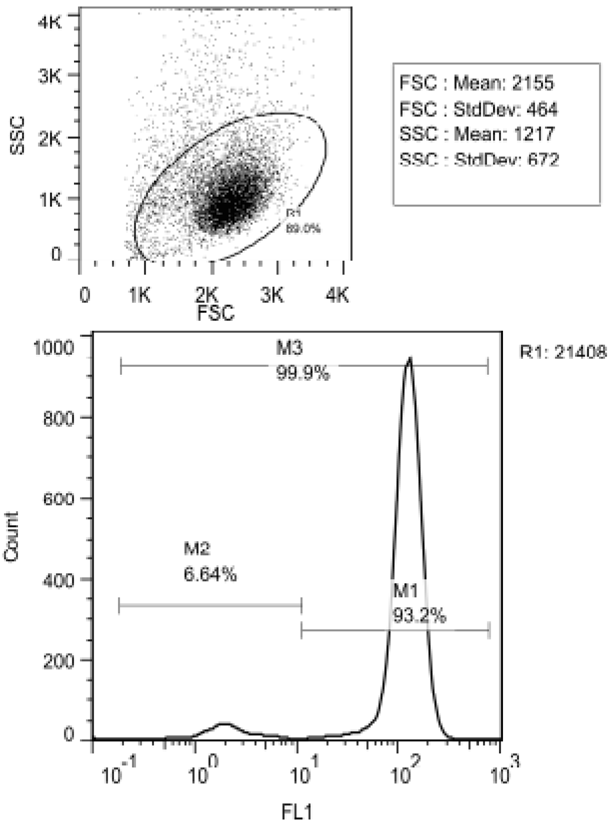

	Mean	StdDev	Count	Peak
All	117	49.3	21408	129
M1	125	39.9	19959	129
M2	2.52	1.85	1422	1.78
M3	117	49.2	21381	129

Figure S54: Flow cytometry data for compound **15** tested at 20 µM in resistant human *ABCB1*-gene transfected L5178Y subline (MDR)

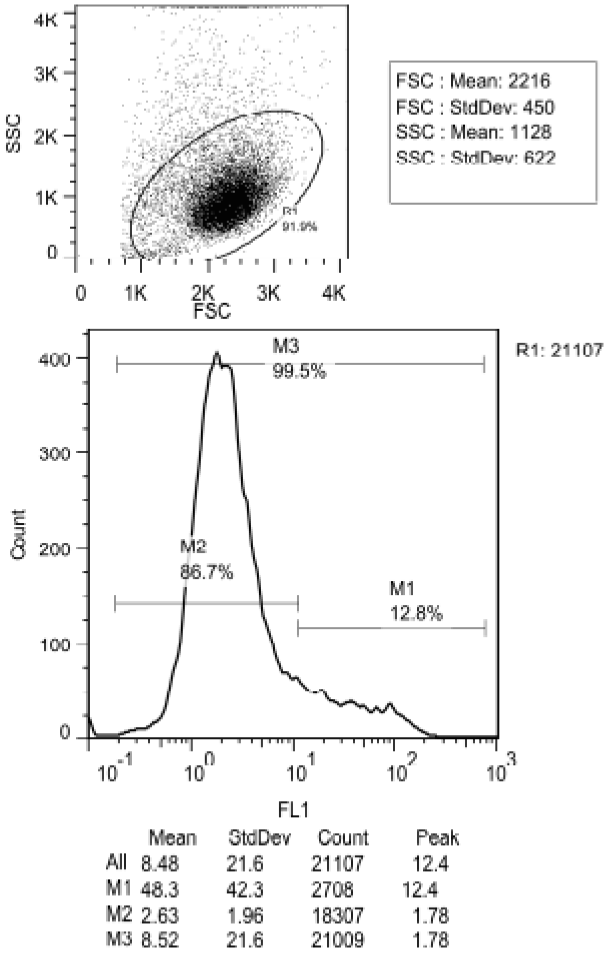

Figure S55: Flow cytometry data for compound **16** tested at 2 µM in resistant human *ABCB1*-gene transfected L5178Y subline (MDR)

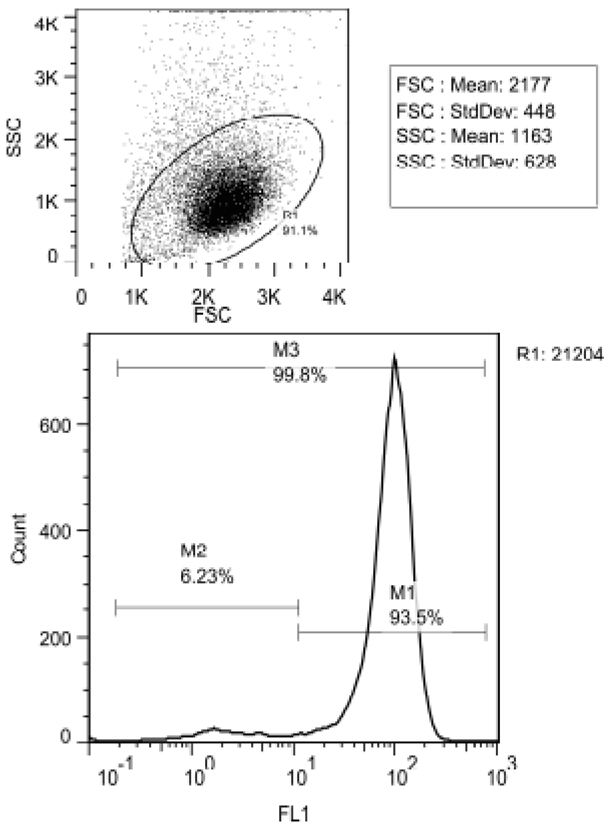

	Mean	StdDev	Count	Peak
All	90.7	45.8	21204	96.5
M1	96.7	41.0	19836	96.5
M2	3.18	2.71	1322	1.60
M3	90.9	45.7	21156	96.5

Figure S56: Flow cytometry data for compound **16** tested at 20 µM in resistant human *ABCB1*-gene transfected L5178Y subline (MDR)

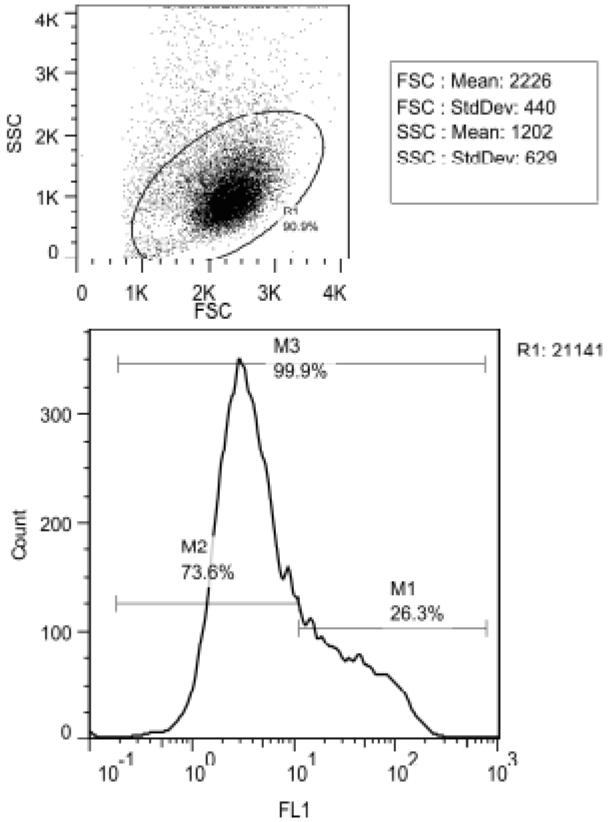

	Mean	StdDev	Count	Peak
All	15.2	28.3	21141	13.8
M1	46.9	40.8	5551	13.8
M2	3.93	2.38	15564	3.28
M3	15.2	28.3	21114	3.28

Figure S57: Flow cytometry data for compound **17** tested at 2 µM in resistant human *ABCB1*-gene transfected L5178Y subline (MDR)

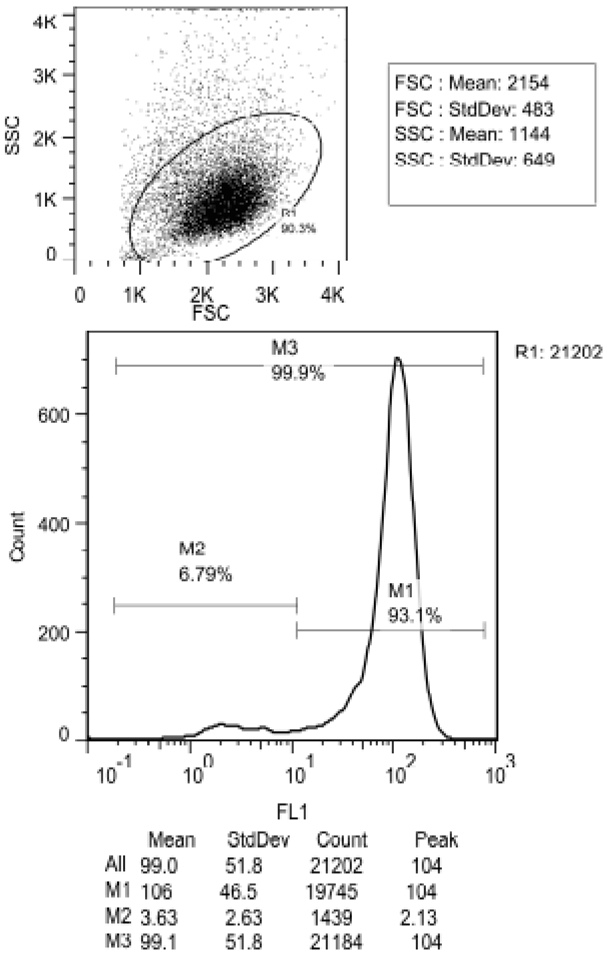

Figure S58: Flow cytometry data for compound **17** tested at 20 µM in resistant human *ABCB1*-gene transfected L5178Y subline (MDR)

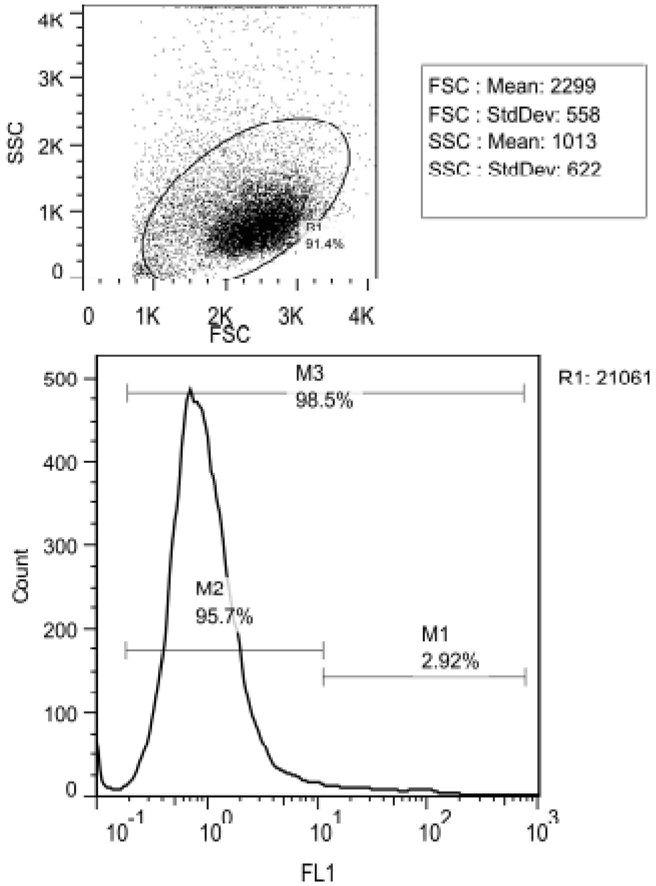

	Mean	StdDev	Count	Peak
All	2.80	14.1	21061	13.8
M1	55.2	55.1	614	13.8
M2	1.21	1.20	20154	0.698
M3	2.80	13.2	20752	0.698

Figure S59: Flow cytometry data for compound **18** tested at 2 μM in resistant human *ABCB1*-gene transfected L5178Y subline (MDR)

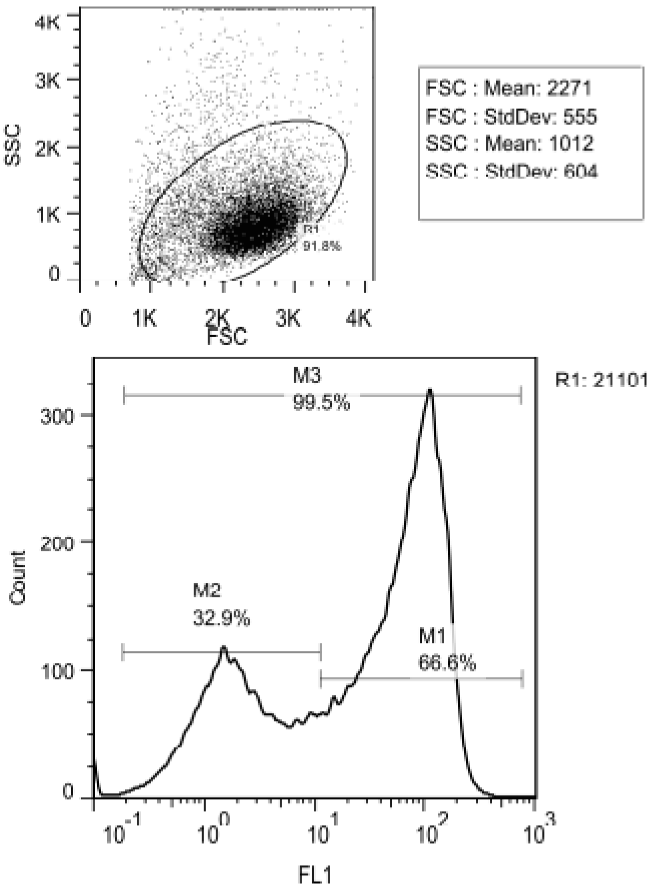

	Mean	StdDev	Count	Peak
All	57.2	57.8	21101	111
M1	84.4	52.9	14047	111
M2	3.02	2.70	6947	1.54
M3	57.5	57.8	20993	111

Figure S60: Flow cytometry data for compound **18** tested at 20 µM in resistant human *ABCB1*-gene transfected L5178Y subline (MDR)

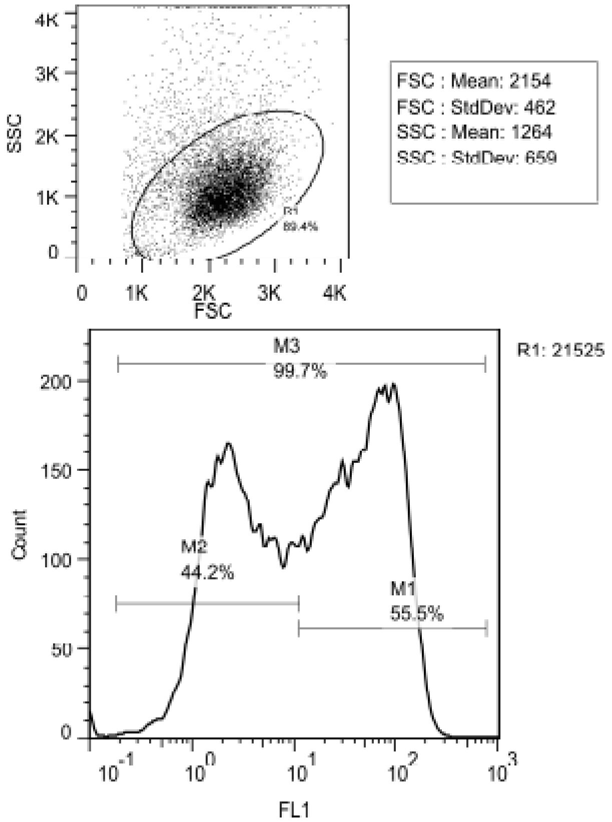

	Mean	StdDev	Count	Peak
All	36.2	44.2	21525	77.7
M1	62.3	44.5	11956	77.7
M2	3.67	2.71	9513	2.21
M3	36.3	44.2	21466	77.7

Figure S61: Flow cytometry data for compound **19** tested at 2 µM in resistant human *ABCB1*-gene transfected L5178Y subline (MDR)

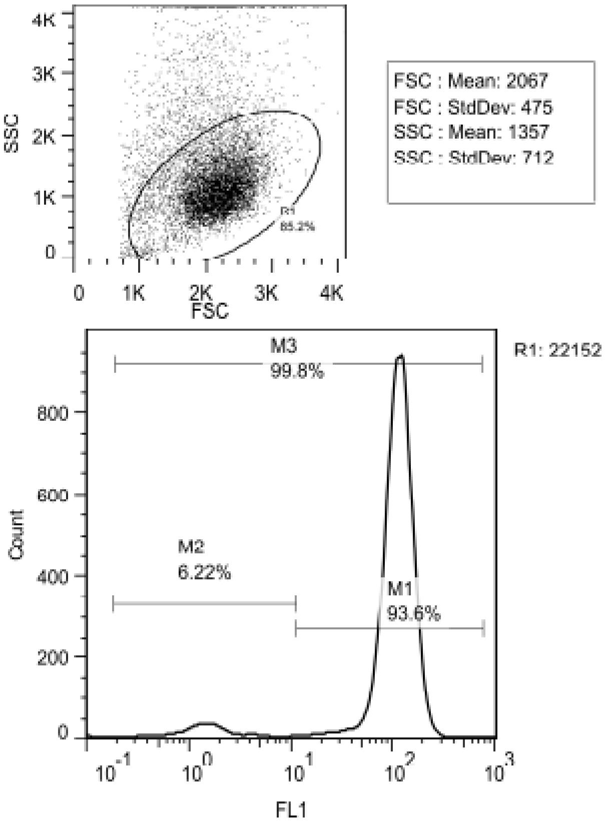

	Mean	StdDev	Count	Peak
All	108	46.1	22152	120
M1	116	38.0	20743	120
M2	1.99	1.85	1377	1.38
M3	109	45.9	22117	120

Figure S62: Flow cytometry data for compound **19** tested at 20 µM in resistant human *ABCB1*-gene transfected L5178Y subline (MDR)

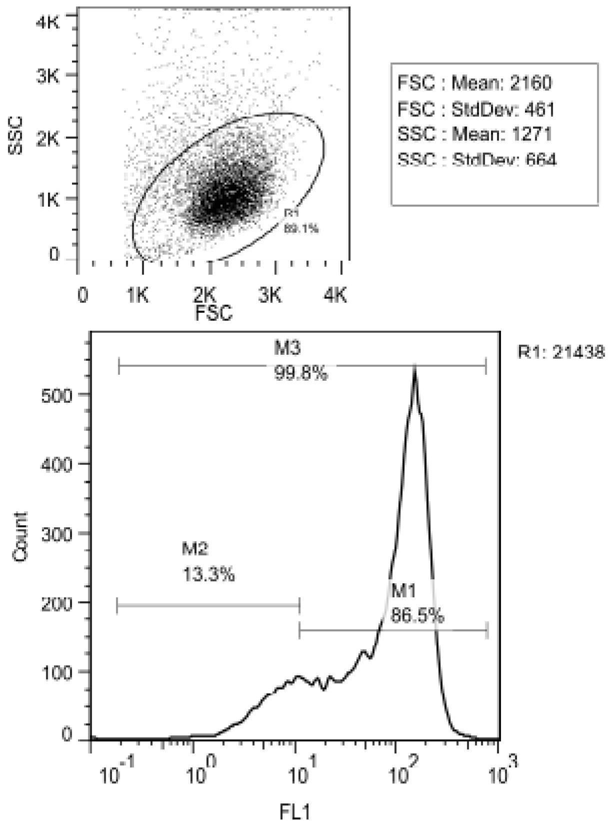

	Mean	StdDev	Count	Peak
All	104	79.8	21438	149
M1	118	72.4	18547	149
M2	6.11	2.81	2859	10.0
M3	103	77.4	21404	149

Figure S63: Flow cytometry data for compound **20** tested at 2 µM in resistant human *ABCB1*-gene transfected L5178Y subline (MDR)

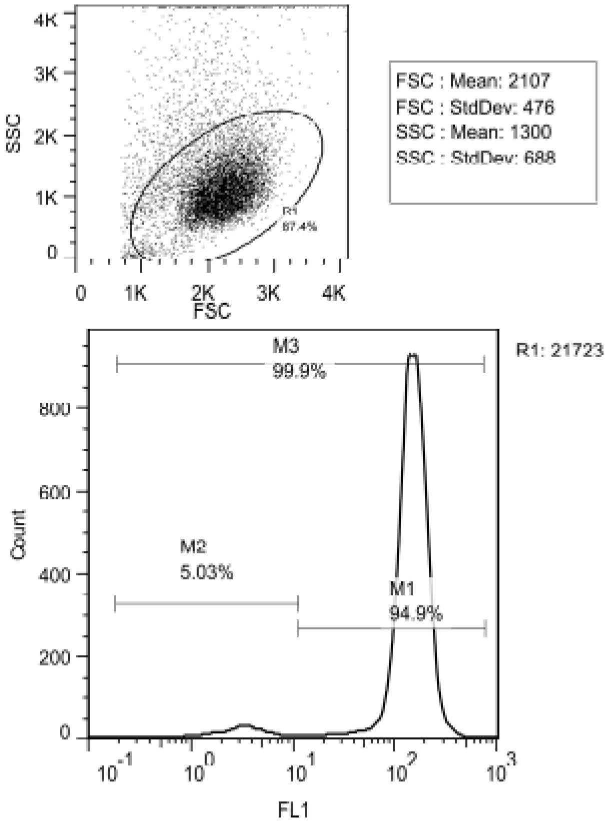

	Mean	StdDev	Count	Peak
All	146	61.4	21723	138
M1	153	52.5	20616	138
M2	3.61	2.11	1092	3.65
M3	146	60.7	21708	138

Figure S64: Flow cytometry data for compound **20** tested at 2 µM in resistant human *ABCB1*-gene transfected L5178Y subline (MDR)

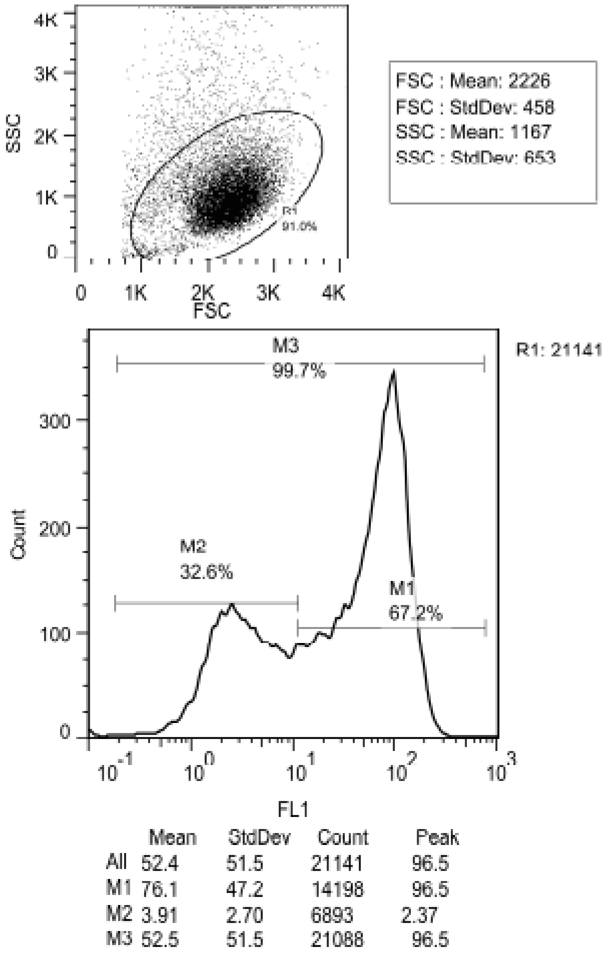

Figure S65: Flow cytometry data for compound **21** tested at 2 µM in resistant human *ABCB1*-gene transfected L5178Y subline (MDR)

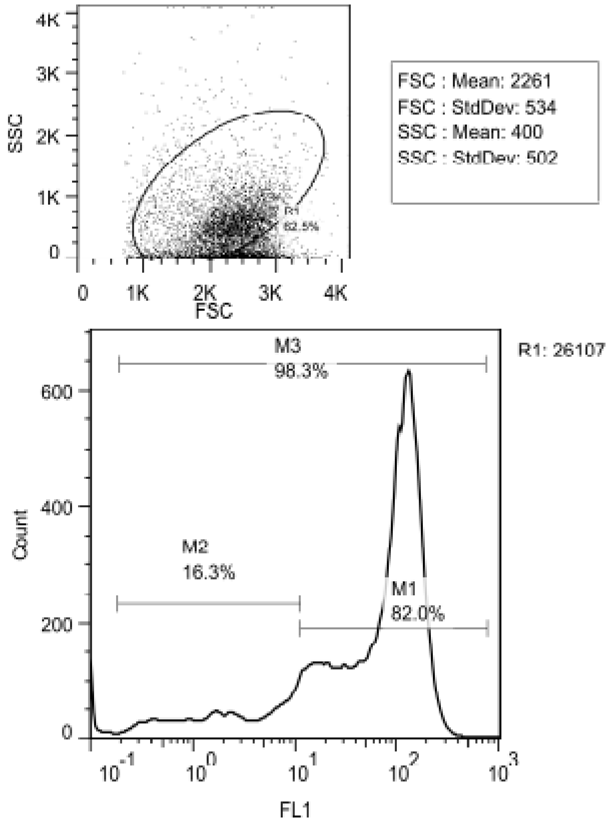

	Mean	StdDev	Count	Peak
All	82.6	65.6	26107	133
M1	100	59.7	21406	133
M2	3.75	3.40	4258	10.4
M3	84.1	65.3	25657	133

Figure S66: Flow cytometry data for compound **21** tested at 20 µM in resistant human *ABCB1*-gene transfected L5178Y subline (MDR)

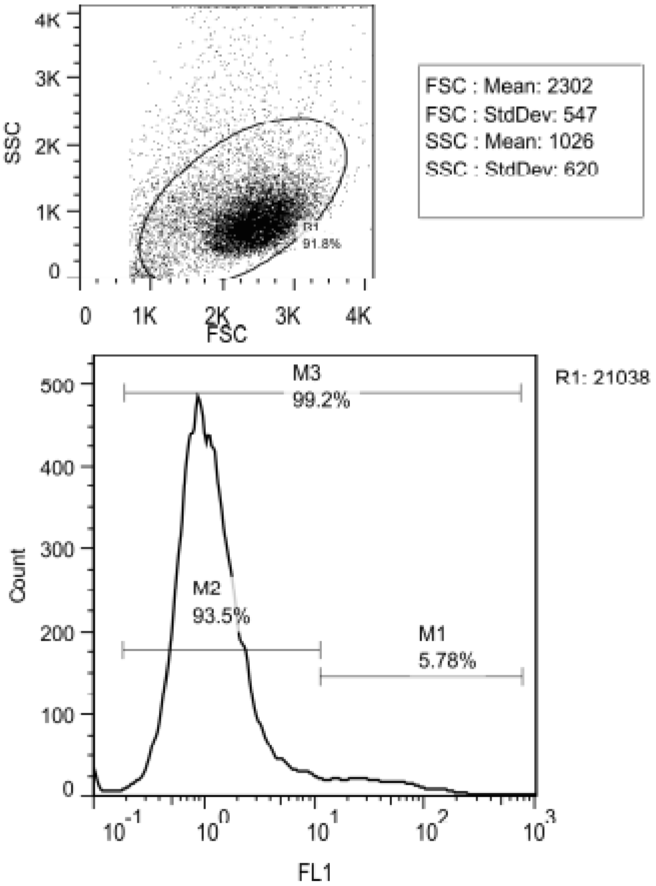

	Mean	StdDev	Count	Peak
All	4.43	17.1	21038	15.4
M1	52.9	50.6	1217	15.4
M2	1.46	1.42	19661	0.866
M3	4.47	17.2	20874	0.866

Figure S67: Flow cytometry data for compound **22** tested at 2 µM in resistant human *ABCB1*-gene transfected L5178Y subline (MDR)

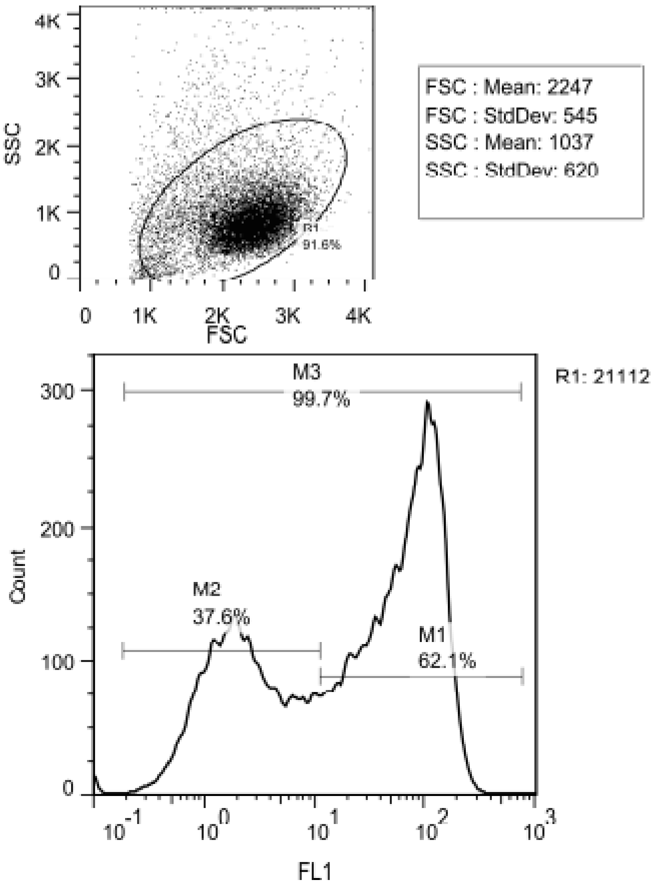

	Mean	StdDev	Count	Peak
All	50.9	55.1	21112	107
M1	80.0	51.5	13110	107
M2	3.13	2.66	7948	1.84
M3	51.0	55.1	21058	107

Figure S68: Flow cytometry data for compound **22** tested at 20 µM in resistant human *ABCB1*-gene transfected L5178Y subline (MDR)

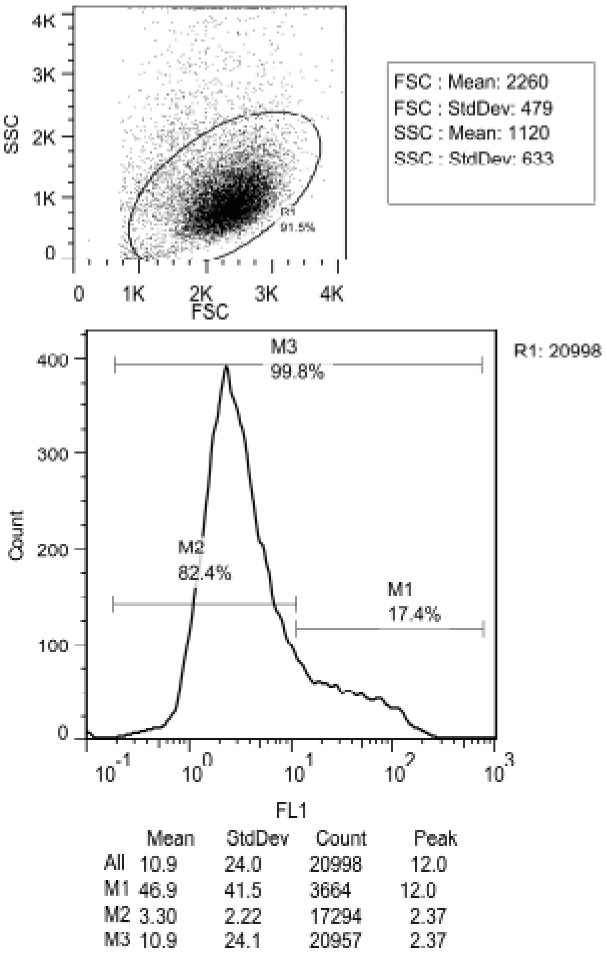

Figure S69: Flow cytometry data for compound **23** tested at 2 µM in resistant human *ABCB1*-gene transfected L5178Y subline (MDR)

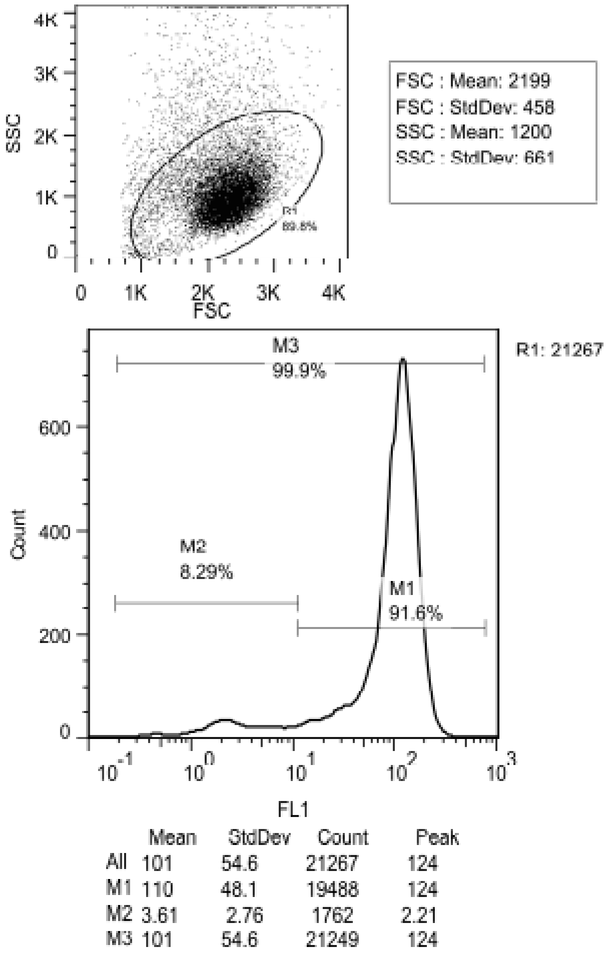

Figure S70: Flow cytometry data for compound **23** tested at 20 µM in resistant human *ABCB1*-gene transfected L5178Y subline (MDR)

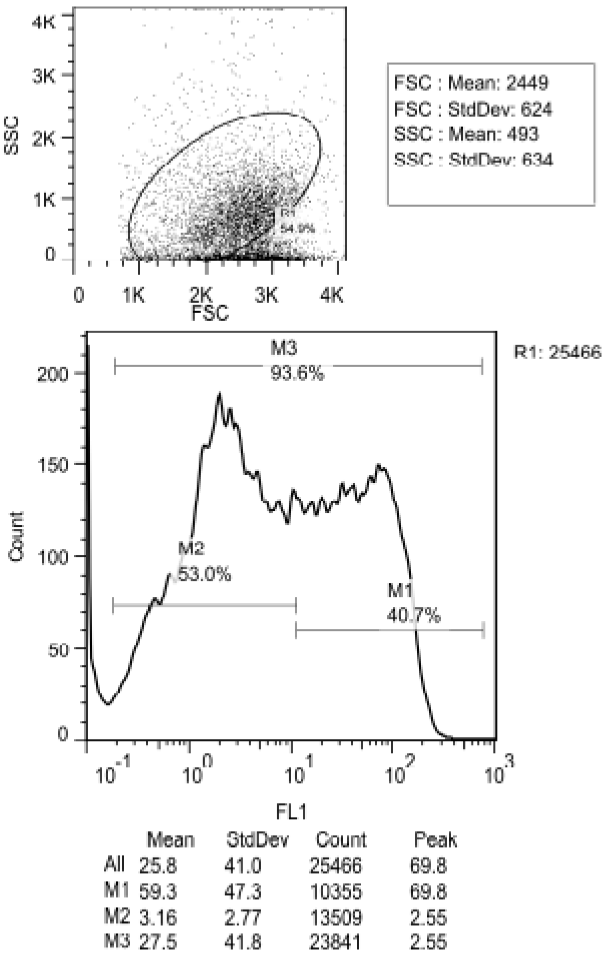

Figure S71: Flow cytometry data for compound **24** tested at 2 µM in resistant human *ABCB1*-gene transfected L5178Y subline (MDR)

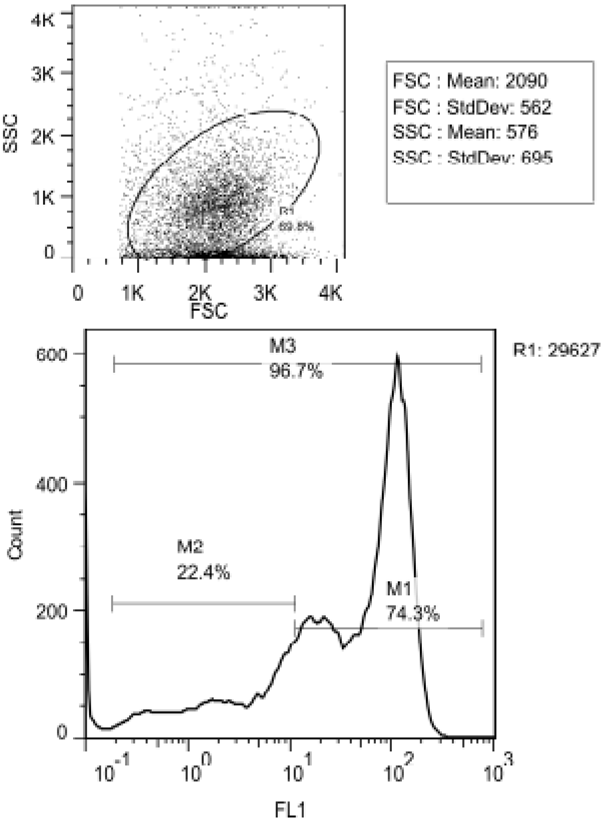

	Mean	StdDev	Count	Peak
All	62.3	56.4	29627	111
M1	82.5	51.7	22024	111
M2	4.10	3.46	6632	10.7
M3	64.4	56.1	28637	111

Figure S72: Flow cytometry data for compound **24** tested at 20 µM in resistant human *ABCB1*-gene transfected L5178Y subline (MDR)

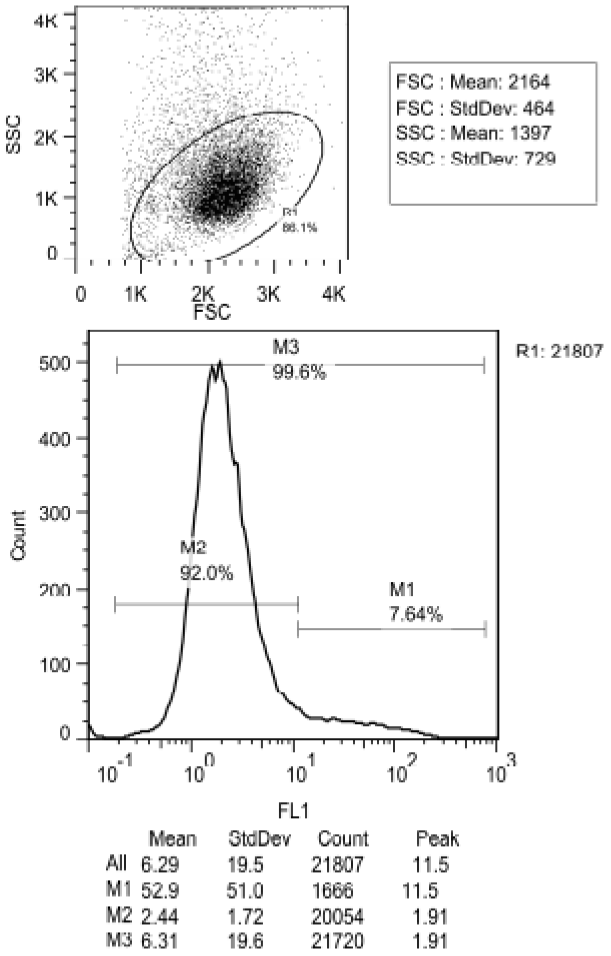

Figure S73: Flow cytometry data for compound **25** tested at 2 µM in resistant human *ABCB1*-gene transfected L5178Y subline (MDR)

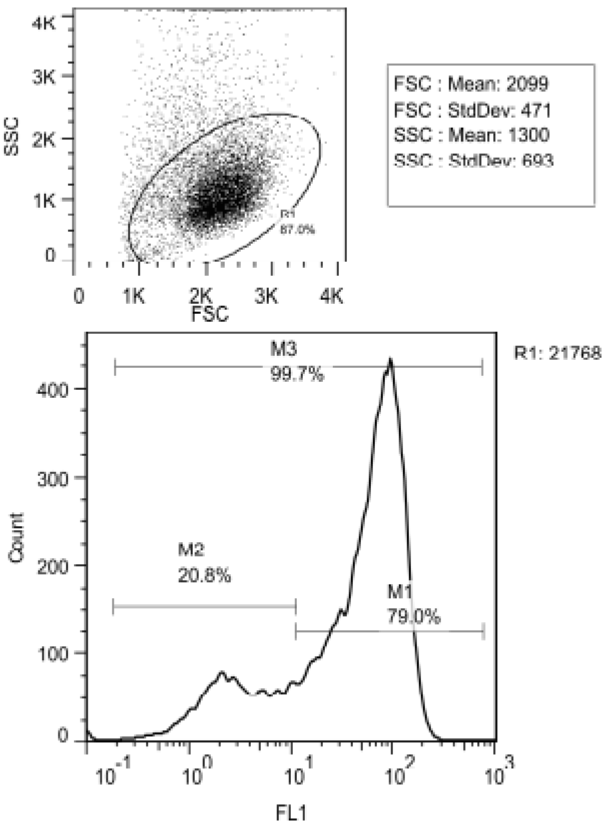

	Mean	StdDev	Count	Peak
All	59.8	47.2	21768	93.1
M1	74.7	42.1	17191	93.1
M2	3.86	2.87	4523	2.13
M3	59.9	47.2	21712	93.1

Figure S74: Flow cytometry data for compound **25** tested at 20 µM in resistant human *ABCB1*-gene transfected L5178Y subline (MDR)

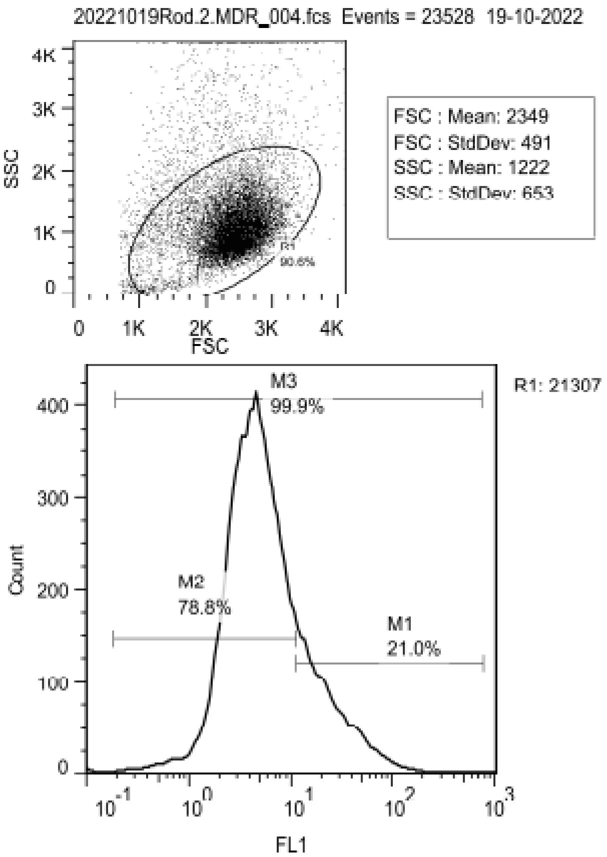

	Mean	StdDev	Count	Peak
All	9.88	16.1	21307	12.0
M1	29.6	26.9	4485	12.0
M2	4.63	2.42	16792	4.53
M3	9.90	16.2	21276	4.53

Figure S75: Flow cytometry data for verapamil tested at 20 µM in resistant human *ABCB1*-gene transfected L5178Y subline (MDR)

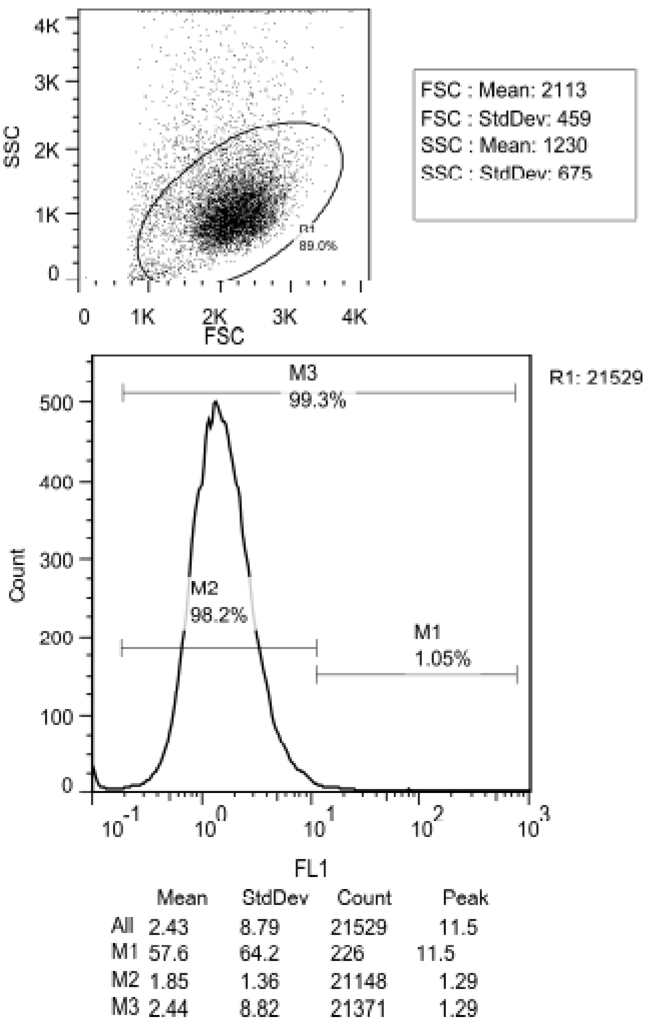

Figure S75: Flow cytometry data for dimethylsulfoxide tested at 2 % in resistant human *ABCB1*-gene transfected L5178Y subline (MDR)

5. Combination chemotherapy results

Table S2: Effect of compounds **1-11** in combination with doxorubicin on resistant human *ABCB1*-gene transfected L5178Y subline (MDR)

Compound	Starting Conc. (μM)	Ratio*	R	CI at ED_{50}	SD	Type of interaction
1	30	1:6.96	0.997	0.98	0.15	Nearly additive
2	38	1:71.41	1.0	1.60	0.21	Antagonism
3	24	1:44.56	1.0	0.21	0.12	Strong synergism
4	47	1:10.9	0.976	0.37	0.05	Synergism
5	11	1:2.55	0.998	0.71	0.11	Moderate synergism
6	18	1:16.72	0.995	0.55	0.11	Synergism
7	30	1:14.14	0.995	0.52	0.05	Synergism
8	24	1:5.45	0.993	0.56	0.80	Synergism
9	24	1:21.8	0.994	0.60	0.06	Synergism
10	30	1:14.14	0.997	0.60	0.04	Synergism
11	30	1:27.84	1.0	0.75	0.08	Moderate synergism

*Ratio: the applied combination and concentration of amine derivatives and doxorubicin (the best combination ratio between compound and doxorubicin). R: Linear Correlation Coefficient. CI at ED_{50}: combination index (CI) at the 50 % growth inhibition dose; CI < 0.1: very strong synergism; 0.1 < CI < 0.3: strong synergism; 0.3 < CI < 0.7: synergism; 0.7 < CI < 0.9: moderate to slight synergism; 0.9 < CI < 1.1: nearly additive; 1.10 < CI < 1.45: moderate antagonism; 1.45 < CI < 3.30: antagonism.

6. Physicochemical properties

Table S3 - *In silico* molecular properties of compounds **1-25** and verapamil using the SwissADME predictive database.[a.]

Compound	Descriptors				
	**Molecular weight (g/mol)**	**LogP**	**HBD**	**HBA**	**TPSA**
1	350.45	2.33	3	5	86.99
2	476.56	3.53	0	8	105.2
3	604.53	4.64	2	8	115.85
4	612.71	4.35	2	10	138.49
5	567.67	3.57	3	8	146.05
6	587.10	4.59	2	8	120.03
7	620.66	5.20	2	11	120.03
8	620.66	4.92	2	11	120.03
9	556.65	3.22	2	9	132.92
10	542.62	3.61	2	9	133.17
11	558.69	4.28	2	8	148.27
12	521.67	3.25	3	7	147.08
13	535.70	3.45	2	7	138.29
14	563.75	4.25	3	7	147.08
15	589.79	4.47	3	7	147.08
16	620.80	3.28	3	9	159.55

Table S3: Continuation

Compound	Descriptors				
	Molecular weight (g/mol)	LogP	HBD	HBA	TPSA
17	583.74	4.41	3	7	147.08
18	597.77	4.69	3	7	147.08
19	597.77	4.79	3	7	147.08
20	611.79	5.10	3	7	147.08
21	628.74	3.78	9	3	192.90
22	601.73	4.87	3	8	147.08
23	597.77	4.60	3	7	147.08
24	633.80	5.38	3	7	147.08
25	687.73	3.91	3	8	160.22
Verapamil	440.58	4.29	3	9	72.74

Octanol-water partition coefficient *(*LogP); number of hydrogen bond acceptors (HBA); number of hydrogen bond donors (HBD); topological polar surface area (TPSA); Lipinski's rule of five: molecular weight < 500 Da; Log P < 5; HBA< 5; HBD < 10. A maximum of 1 violation is permitted.

Table S4: *In silico* pharmacokinetic (absorption, distribution, metabolism, and excretion) and toxicity parameters of compounds **1-25** and verapamil using pkCSM and SwissADME predictive databases.

Compound	Log S (mol/L)	Caco-2 Permeability (log Papp in 10^{-6} cm/S	Intestinal Absorption (%)	Fractional unbound (fu)	CNS permeability (logPS)	CYP3A4 inhibitor (Yes/No)	Hepato toxicity	AMES toxicity
1	-3.10	1.35	95.36	0.20	-2.47	No	No	No
2	-5.03	0.93	90.04	0.09	-2.59	Yes	No	No
3	-5.15	0.66	86.66	0.05	-2.74	Yes	Yes	No
4	-5.85	0.35	82.28	0	-3.03	Yes	Yes	No
5	-4.92	0.02	76.54	0.13	-2.84	Yes	Yes	No
6	-5.62	0.80	84.80	0	-2.65	Yes	No	No
7	-5.71	0.81	85.94	0	-2.64	Yes	Yes	No
8	-5.71	0.81	85.91	0	-2.63	Yes	Yes	No
9	-5.05	0.75	79.14	0.05	-2.95	Yes	Yes	No
10	-4.73	0.68	82.88	0.11	-2.90	Yes	Yes	No
11	-5.24	0.88	83.97	0	-2.76	Yes	Yes	No
12	-4.43	0.78	70.34	0.20	-3.07	No	No	No
13	-4.59	0.83	71.06	0.18	-3.02	No	Yes	No
14	-4.57	0.90	73.68	0.06	-2.81	No	Yes	No
15	-5.04	0.76	78.64	0.10	-2.90	No	Yes	No
16	-4.15	0.73	64.04	0.31	-3.39	No	Yes	No
17	-4.92	0.89	79.83	0.04	-2.68	No	Yes	No
18	-4.98	0.89	80.29	0.03	-2.63	No	No	No
19	-4.98	0.79	80.28	0.01	-2.63	No	Yes	No
20	-5.07	0.82	80.36	0.02	-2.64	Yes	No	No
21	-4.85	-0.27	76.62	0.06	-2.84	Yes	Yes	No
22	-4.92	0.89	80.17	0.05	-2.74	No	No	No
23	-5.32	0.87	77.41	-0.65	-2.76	No	Yes	No

Table S4: Continuation

Compound	Log S (mol/L)	Caco-2 Permeability (log Papp in 10^{-6} cm/S	Intestinal Absorption (%)	Fractional unbound (fu)	CNS permeability (logPS)	CYP3A4 inhibitor (Yes/No)	Hepatotoxicity	AMES toxicity
24	-4.61	**0.90**	86.82	0.06	-2.46	Yes	No	No
25	-4.87	0.66	76.65	0.12	-2.98	No	Yes	No
Verapamil	-5.23	1.11	97.10	0.009	-2.58	Yes	No	No

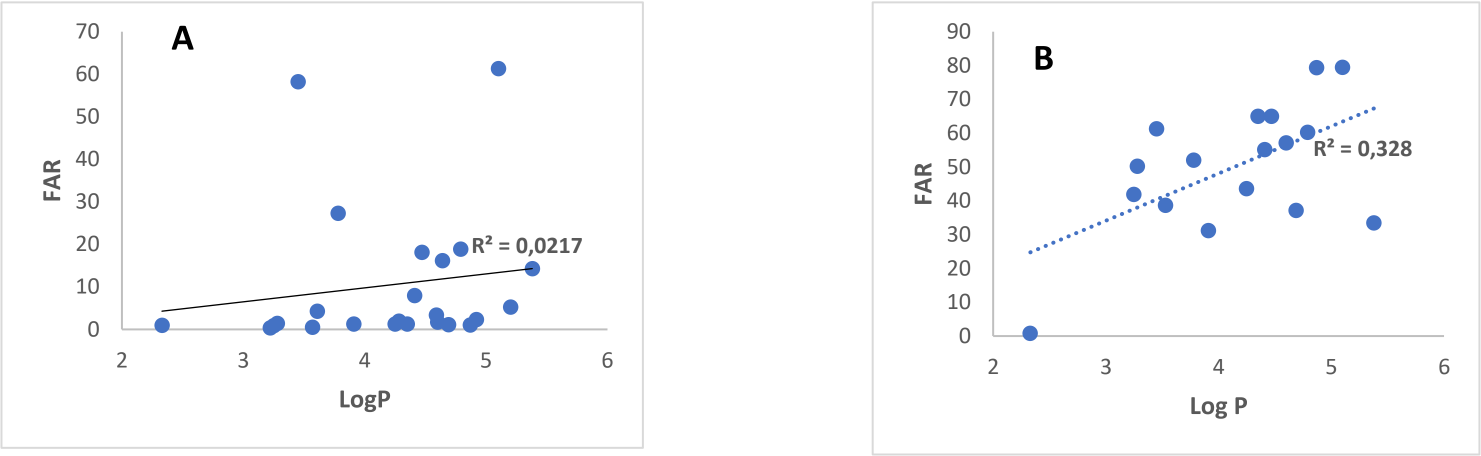

Figure S76: Representative plots of Log P versus FAR values for tested compounds in ABCB1-transfected mouse T-lymphoma cells at (A) 2 µM (compounds 1-25) and (B) 20 µM (compounds 1, 2, 4 and 12-25).

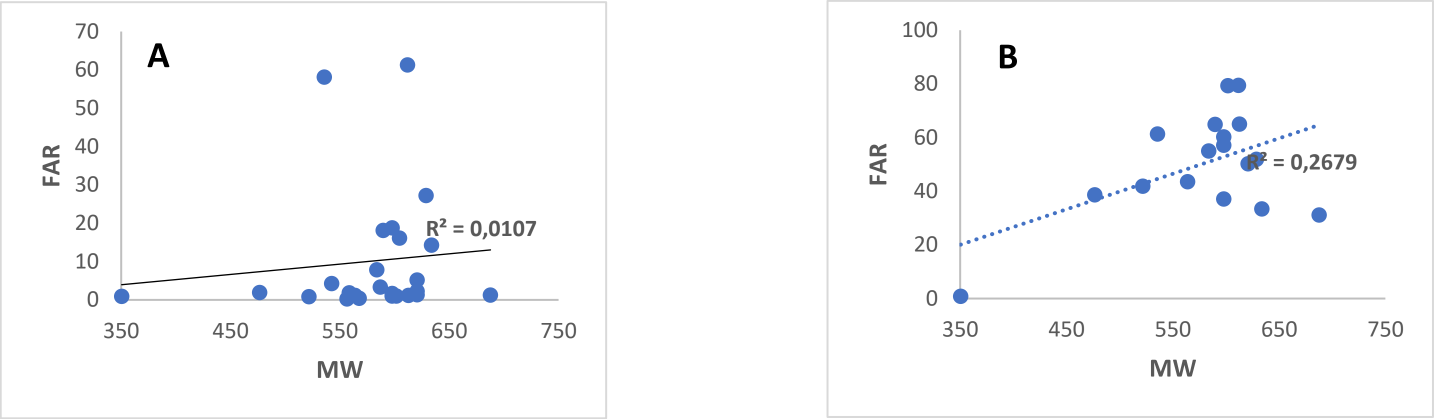

Figure S77: Representative plots of molecular weight (MW) versus FAR values for tested in ABCB1-transfected mouse T-lymphoma cells at at (A) 2 µM (compounds 1-25) and (B) 20 µM (compounds 1, 2, 4 and 12-25).

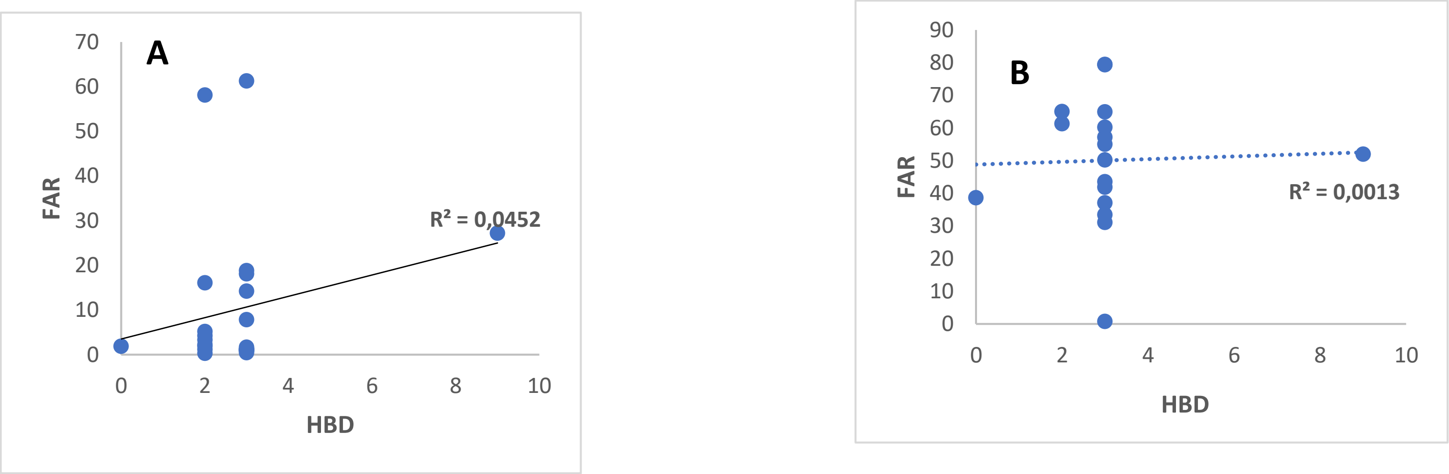

Figure S78: Representative plots of H-bond donors (HBD) versus FAR values for tested compounds in ABCB1-transfected mouse T-lymphoma cells at at (A) 2 µM (compounds 1-25) and (B) 20 µM (compounds 1, 2, 4 and 12-25).

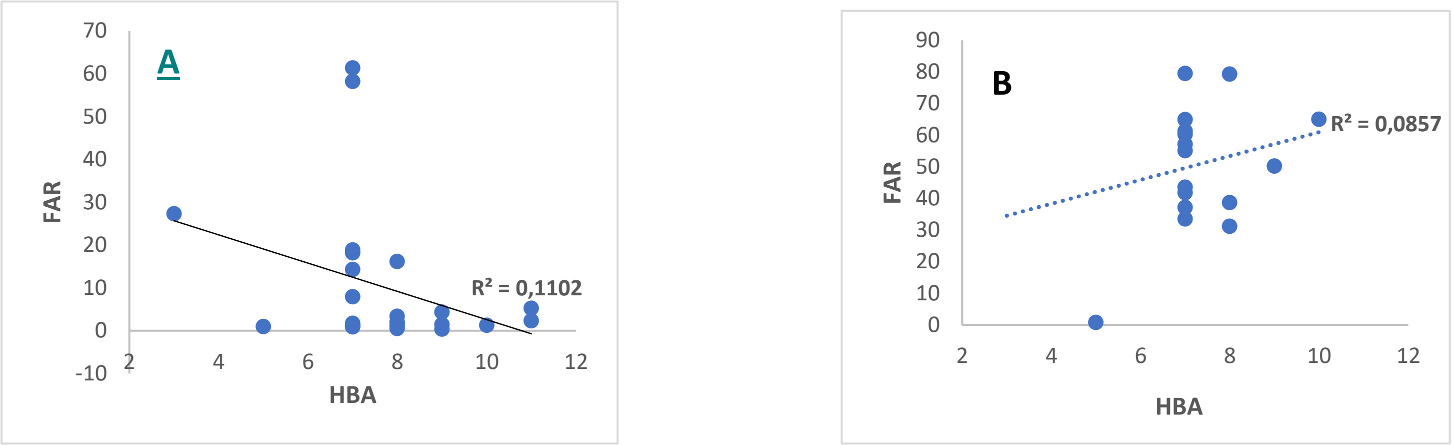

Figure S79: Representative plots of H-bond acceptors (HBA) versus FAR values tested compounds in ABCB1-transfected mouse T-lymphoma cells at at (A) 2 µM (compounds 1-25) and (B) 20 µM (compounds 1, 2, 4 and 12-25).

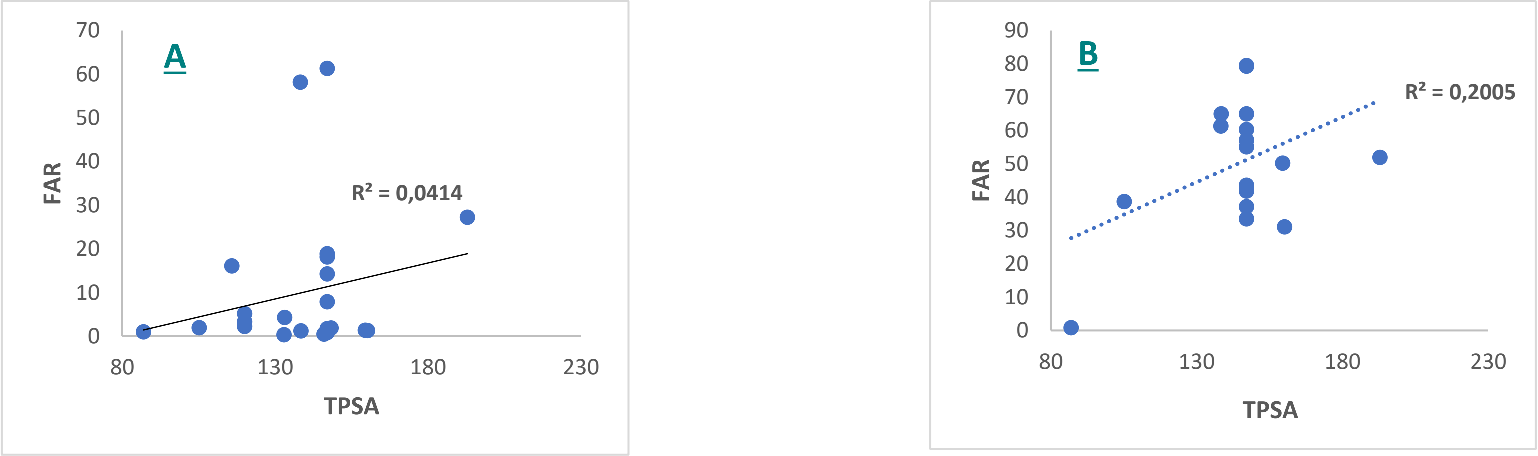

Figure S80: Representative plots of topological polar surface area (TPSA), versus FAR values tested compounds in ABCB1-transfected mouse T-lymphoma cells at at (A) 2 µM (compounds 1-25) and (B) 20 µM (compounds 1, 2, 4 and 12-25).

7. References

1 A. E. Owen, H. Louis, E. U. Ejiofor, W. Emori, T. E. Gber, I. Benjamin, C. R. Cheng, M. M. Orosun, L. Ling and A. S. Adeyinka, *Chem. Africa*, 2023, **6**, 2445–2461.

2 E. Villedieu-Percheron, V. Ferreira, J. F. Campos, E. Destandau, C. Pichon and S. Berteina-Raboin, *Foods*, 2019, **8**, 683–693.
